# Large-scale data-driven inverse EM design based on metacircuit-embedded surface

**DOI:** 10.1038/s41467-026-75411-z

**Published:** 2026-07-13

**Authors:** Ming Xu, Song Zha, Mingtuan Lin, Hanqing Liu, Jihong Zhang, Yanlin Xu, Huan Jiang, Qiang Cheng, Peiguo Liu

**Affiliations:** 1 College of Electronic Science and Technology, National University of Defense Technology, Changsha, Hunan China; 2 State Key Laboratory of Millimeter Waves, Southeast University, Nanjing, Jiangsu China

**Keywords:** Electrical and electronic engineering, Computer science

## Abstract

General-purpose electromagnetic model is central to intelligent electromagnetic applications. However, its development is limited by severe scarcity of training data, arising from the high computational cost of full-wave simulations and reliance on specialized topologies that yield single EM function. To overcome this challenge, we introduce a metacircuit-embedded surface (MCES) framework, consisting of a fixed structural topology and flexible metacircuits that can be realized through lumped components. By shifting design focus from full-wave simulation to circuit-level modeling, over six million samples can be easily generated with laptop computation resources. Subsequently, an MCES-based forward and inverse AI design method was developed, providing rich design capability in frequency, amplitude, phase and polarization domains. Finally, three kinds of classical metasurfaces were designed and fabricated, showing superior performance compared to reported advanced designs. Overall, the proposed MCES-based framework greatly raises the design efficiency and training scale, paving the way for future general-purpose electromagnetic model.

## Abstract (web summary)

General EM models face training challenges due to scarcity of training data. Here, authors introduce a metacircuit-embedded surface framework that enables rapid generation of millions of training samples for AI-driven metasurface design, providing rich design capabilities in multiple domains.

## Introduction

Electromagnetic (EM) metasurface, composed of periodic or non-periodic geometric arrays with subwavelength unit cells^1–4^, has brought significant innovation in the areas of 5G/6G communications^5–8^, imaging^9–12^, sensing^13–16^, electromagnetic cloaking^17–20^ and electromagnetic compatibility^21–23^. The rise of artificial intelligence has further accelerated metasurface design, overcoming the limitations of trial-and-error methods and enabling the rapid generation of microstructures for sophisticated electromagnetic manipulation^24–27^. Neural networks including convolutional neural networks (CNNs)^28^, recurrent neural networks (RNNs)^29^, generative adversarial networks (GANs)^30^, and deep neural networks (DNNs)^31^ have been widely employed in metasurface design. For instance, Perry Foy et al. developed an artificial neural network for inverse designing multilayer nanoparticle arrays using 50,000 training samples^32^. Subsequent efforts have further diversified the methodological landscape. Zhixiang Fan et al. developed a deep learning model using a dataset of 84,400 far-field and wireless channel samples for homeostatic neuro-metasurfaces design^33^, while Liu et al. used GAN to achieve reverse design of metasurfaces, completing it through 50,000 iterations and 6500 training data^34^. Nadell et al. combined deep learning with forward dictionary search using 18,000 training samples^35^. These methods have achieved notable performance in designing plasmonic metasurfaces, multilayer nanoparticle arrays, and anisotropic coding metasurfaces for functions including broadband absorption, beam focusing, and anomalous reflection. Despite achieving strong performance in metasurface design, existing AI-driven approaches face two fundamental limitations that hinder their scalability toward general-purpose electromagnetic model.

The first constraint is data scarcity. Building a large-scale electromagnetic model requires massive training datasets, yet current methods predominantly rely on full-wave simulations for data generation^36,37^. Since typical complex structure’s simulation takes minutes to complete, accumulating millions of data points would require years of computation, making this approach practically infeasible^38,39^. Furthermore, such limited datasets impose excessive demands on algorithmic robustness and severely restrict model generalization^40–42^.

The second limitation is the low efficiency of building general-purpose electromagnetic model. Most current AI models are trained on data from single fixed topologies. locking them to limited functional domains^43,44^. Although some researchers have applied a unified model architecture to achieve multiple designs, such as Lv Hao et al. who constructed models through parameterized scanning of various typical frequency-selective surfaces(FSS), achieving FSS designs from single-layer to multi-layer structures, this model is limited to searching and reassembling within previously observed topological features, ultimately still constructing small-scale models with single functionalities^45^. Consequently, developing a comprehensive electromagnetic model would require assembling numerous specialized small models, with each targeting different functions and topologies. This not only creates an inefficient modeling process where each unit requires separate training and cannot be repurposed, but also makes it practically difficult to select optimal topologies for target functions.

To efficiently construct electromagnetic model, this study proposes a generalizable metasurface design framework that maintains a fixed meta-atom topology, such as a linear, cross, or ring-type structure, while varying only the integrated metacircuit to achieve different functionalities. The metacircuit consists of resistive, inductive, capacitive, and diode elements, which can be realized using lumped components or micro-nano processes to minimize parasitic effects and facilitate low-cost, large-scale fabrication. With this approach, various metasurface functions can be attained simply by replacing the metacircuit, without altering the underlying structure. By mapping the design space from a high-dimensional geometric domain where discontinuities arise from switching between distinct topological families to a low-dimensional continuous circuit parameter space within a fixed topology, our method significantly reduces optimization complexity. Moreover, replacing full-wave simulations with fast circuit-based models accelerates training data generation by more than 300 times, enabling large-scale, data-driven AI design. Leveraging this efficiency, we constructed a comprehensive dataset comprising over 6 million sample pairs across multiple topologies, ensuring broad coverage of metasurface types. Combined with a classic DNN architecture and optimization algorithms, this dataset enabled the training of a model capable of efficiently designing diverse metasurfaces. This capability has been validated across hundreds of design cases, demonstrating effective modulation over responses in the frequency, amplitude, polarization, and phase domains. Furthermore, three distinct functional metasurfaces were designed, fabricated, and tested to validate the model, with all exhibiting excellent performance compared to advanced reported designs. From a broader perspective, the proposed MCES framework addresses key obstacles in electromagnetic modeling, including data scarcity, limited model reusability, and prohibitive simulation costs. It serves not only as a practical tool for device design but also as a foundational data infrastructure, paving the way for future universal and scalable EM large models.

## Results

This section presents the development and experimental validation of the MCES framework. The study begins by demonstrating the framework’s feasibility and its advantages over conventional design methods. It is further demonstrated how the framework enables generation of extremely large training datasets to overcome the data scarcity that limits conventional approaches. These data support a highly efficient and accurate machine-learning workflow that integrates forward prediction with inverse design. This unified approach enables accurate design of functional metasurfaces within seconds. Finally, experimental validation confirms the approach’s capabilities through complete design, fabrication, and measurement of three distinct metasurfaces, all achieving performance that matches or surpasses advanced designs.

### Metacircuit-embeded surface-based EM model framework

The scarcity of training data and inefficient design methodologies pose a major challenge to building general-purpose EM models. To address these issues, this paper introduces the metacircuit-embedded surface (MCES) framework, a design strategy based on a simple structure with complex metacircuits. The core metasurface unit maintains a fixed geometry, specifically a cross-shaped metallic element, and integrates various lumped components such as resistors, inductors, capacitors, and diodes into its gap. A fixed electromagnetic structure is combined with reconfigurable complex circuits to achieve multiple electromagnetic functions. By leveraging rapid circuit-level simulation within this unified architecture, our work paves the way for efficient construction of general-purpose EM models.

**Fig. 1:**
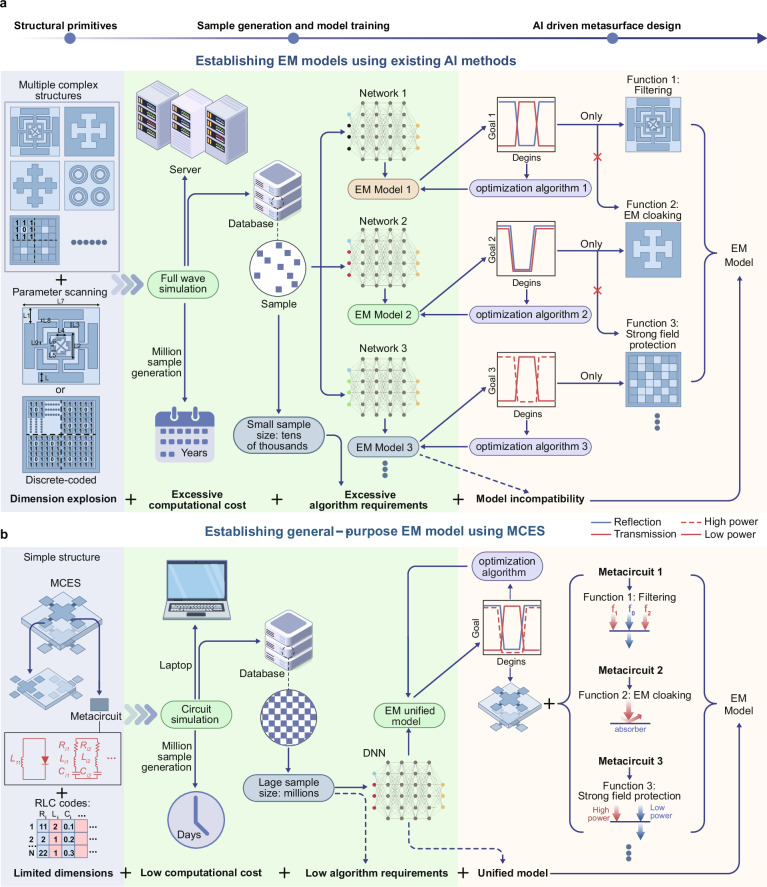
The difference between efficient MCES framework and traditional model framework. **a** Conventional AI-driven workflow. This approach relies on full-wave simulation of diverse, discretely-encoded topologies, leading to high-dimensional inputs, expensive computation, and functionally isolated small-scale models. **b** Proposed MCES-based framework. Using a fixed topology with an embedded tunable metacircuit, the method enables millisecond-level sample generation, permitting the creation of millions of samples on a consumer-grade laptop and resulting in a unified, general-purpose electromagnetic model.

The AI-based workflow for constructing an EM model primarily encompasses structural parameter extraction, training data acquisition, model training, and final model assembly. Key differences between the proposed MCES framework and conventional design methods are illustrated in Fig. 1.

As illustrated in Fig. 1a, the prevailing AI-driven approach for metasurface design typically starts with mathematically representing complex topologies via parameter scanning or discrete encoding. Since each structure is defined by multiple parameters with wide value ranges, the input dimensionality expands drastically. Furthermore, finer structural discretization and higher-dimensional encoding exacerbate this problem, leading to an explosion in input dimensions. Each encoded structure then undergoes full-wave simulation, a computationally expensive process that requires several minutes per simulation and often demands high-performance computing servers. Generating a dataset of millions of samples would take years, limiting practical workflows to only a few thousand samples per project. This constrained sample size covers only a narrow topological range and places high demands on the AI algorithms used for modeling. Moreover, because each trained model is tied to a specific topological structure and thereby to a single metasurface function, the resulting specialized models are functionally limited and mutually incompatible. Consequently, a comprehensive electromagnetic model community can only be assembled as a collection of isolated small-scale models. Issues such as high-dimensional inputs, prohibitive computational cost, stringent algorithm requirements, and model interoperability have collectively restricted current AI methods to limited-scale electromagnetic modeling.

The process of constructing an EM large model using the MCES framework is illustrated in Fig. 1b. The MCES framework employs a fixed metasurface architecture integrated with a tunable metacircuit, which serves as a complex functional block that can be realized using lumped elements or micro-nano components embedded into the metasurface structure. Due to the fixed topology, each MCES configuration can be fully described by a set of parameters. Different metasurface functions are achieved simply by altering the metacircuit design. Since the metacircuit is defined by a limited number of lumped parameters, it can be efficiently modeled using circuit-level simulation, which operates at millisecond speeds. This enables the generation of millions of training samples within days on a consumer-grade laptop. The resulting large-scale dataset reduces the demands on the AI training process. Moreover, because all designs are generated within the same unified framework, a single trained model can generalize across a wide variety of metasurface functions. Owing to its low-dimensional parameter space, minimal computational cost, reduced algorithm complexity, and unified design model, the MCES method provides a feasible and efficient pathway toward constructing general-purpose EM models.

### High-functionality of metacircuit-embeded-surface

**Fig. 2:**
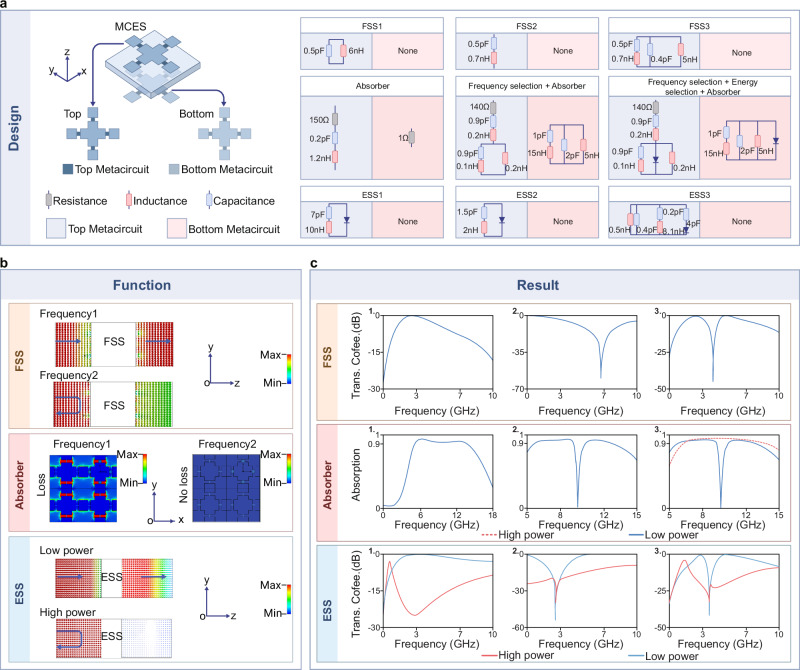
High-functionality MCES design. **a** Versatile functionality via metacircuit integration. Distinct metasurface functions are realized by integrating different circuit (top and bottom metacircuit) into the top and bottom layers of the MCES. **b** Metasurface functionality demonstration. FSS transmits at *f*_1_ and blocks *f*_2_; absorber dissipates energy at *f*_1_ while transmitting at *f*_2_; energy-selective surfaces(ESS) switches from transmission (low-power condition) to shielding (high-power condition). **c** Quantitative performance comparison. The proposed MCES demonstrate simultaneous miniaturization and bandwidth enhancement compared to established designs. (see Supplementary Information Section 2).

An efficient AI-assisted large-scale model crucially depends on a metasurface framework with a simple topology and versatile functionality. High Functionality describes the capacity of a framework to support the uniform design of diverse metasurface functions. To verify this high functionality, we performed 280 metasurface designs using the proposed framework, demonstrating precise modulation across responses in the frequency, amplitude, polarization, and phase domains (see Supplementary Information Section 2). Firstly, metasurfaces with different frequency responses were designed to achieve band-stop, band-pass, dual-band-stop, and dual-band-pass filtering. In the amplitude domain, both passive and active adaptive designs were developed to achieve diverse amplitude responses including absorption and adaptive reflection. Third, within the phase domain, gradient modulation was applied to the phase of both reflected and transmitted waves. Finally, in the polarization domain, we realized modulation of the polarization response, enabling conversion from linear to circular polarization. By modulating different responses, we can design metasurfaces with specific functions. Based on the rich modulation functions, we selected three major categories that include nine representative metasurface functions for validation, as illustrated in Fig. 2.

As shown in Fig. 2a, we demonstrate the design of diverse metasurfaces using the MCES framework. This is achieved by maintaining a fixed metallic structure while integrating different metacircuits at the top and bottom layers. Specifically, the integration of distinct top-layer metacircuits has yielded a bandpass FSS (FSS1), a bandstop FSS (FSS2), and a dual-band bandpass FSS (FSS3), confirming the high-functionality of MCES for passive metasurface design. The performance of these designs shows significant improvement over prior works, as shown in Fig. 2c, with the bandwidth of FSS1 increased by 218% compared to ref. ^46^. Furthermore, FSS2 exhibits a 59% bandwidth increase over^47^, while FSS3 maintains the same center frequency as ref. ^48^ and achieves bandwidth enhancements of 142% and 23% in its two bands, respectively.

Similarly, to verify the high-functionality of MCES in multifunctional surface design, we designed an absorber, an absorption frequency-selective reflector, and an absorption energy-selective reflector. As shown in Fig. 2c, the designed absorber more than doubles the absorption bandwidth compared to ref. ^49^. The absorption frequency-selective reflector integrates a bandstop FSS with an absorbing structure, exhibiting dual absorption bands at 6.5–9 GHz and 10.1–13.4 GHz, and a reflection band from 9.56 to 9.8 GHz. The absorption energy-selective reflector exhibits dual absorption bands (6–9.3 GHz and 10.5–12.6 GHz) and a narrow reflection band (9.6–9.8 GHz) under low-power conditions, successfully combining the functions of an absorber and a bandstop FSS. Under high-power conditions, its absorption rate exceeds 90% across 6–13.6 GHz, confirming the integration of absorber, bandstop FSS, and ESS functionalities. This result robustly demonstrates the capability of MCES for advanced multifunctional metasurface design.

Finally, to verify the high-functionality of MCES in active metasurfaces design, we designed bandpass ESS (ESS1), bandstop ESS (ESS2), and dual-band bandpass ESS (ESS3). As shown in Fig. 2c, the performance of these designs significantly surpasses that of previous works. Specifically, ESS1 achieves a threefold increase in bandwidth with an eightfold reduction in size compared to ref. ^50^. Furthermore, ESS2 attains a stopband from 1.75 to 3.6 GHz under low-power conditions, which expands to cover 0–8.5 GHz under high-power conditions. ESS3 maintains the same center frequency as ref. ^51^ with comparable bandwidth in both passbands, while achieving a more compact form factor. (see Supplementary Information Section 2)

### High-efficiency AI-assisted training based on MCES

**Fig. 3:**
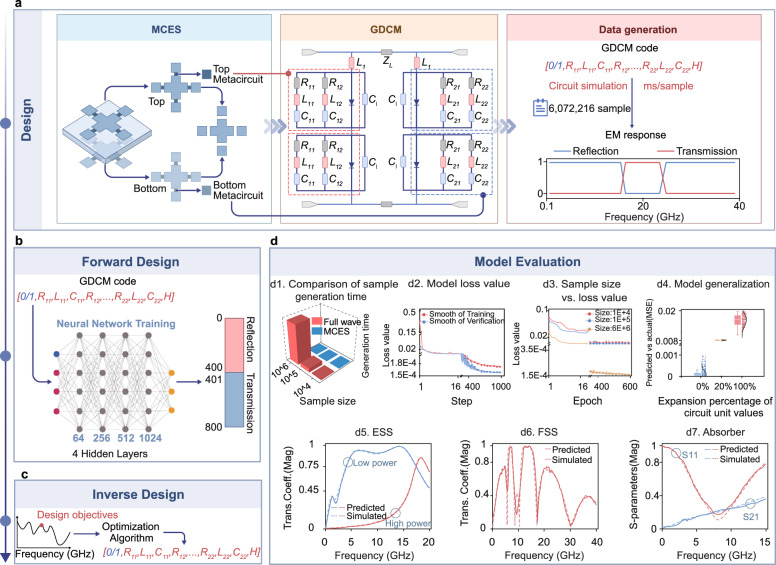
High-efficiency AI-assisted training based on MCES. The design process consists of three core stages: **a** training set acquisition: A dataset of 6 million training pairs is generated using the GDCM framework and RF circuit simulations. The GDCM is derived from the MCES equivalent circuit. We employ a double-layer cross-shaped structure as the MCES base framework. The entire structure is represented by a series inductor (*L*_*I*_) and capacitor (*C*_*I*_). Introducing circuits into the gaps is equivalent to connecting them in parallel with *C*_*I*_. Circuit elements are parameterized as follows: diode states are binary (0 for absence, 1 for presence), while resistors, capacitors, and inductors are encoded by their actual values. Thus, each GDCM configuration is uniquely represented by a code. Using Python for automated parameter stepping, we generated 6 million unique GDCM codes. **b** Forward design: A fully connected neural network is trained to establish a forward model that predicts the electromagnetic response from a given GDCM code. **c** Inverse design: An optimization algorithm is employed to rapidly identify the optimal GDCM code that produces a target electromagnetic response. **d** Performance evaluation: **d1**: Orders-of-magnitude acceleration in dataset generation. **d2**: The value of the loss function during training. **d3**: The relationship between sample size and the loss function, showing accelerated decay and lower converged values with increasing data. **d4**: Model generalization ability. We extended all circuit parameters (*R*_*i*,*j*_, *L*_*i*,*j*_, *C*_*i*,*j*_) by 20% and 100% beyond their original ranges. The corresponding MSE results demonstrate excellent generalization, with values below 8.3 × 10^−3^ and 2 × 10^−2^, respectively. This robustness is attributed to the large training dataset. **d5**–**d7**: Prediction-simulation comparisons for **d5** ESS, **d6** FSS, and **d7** absorber under empirical input codes.

The establishment of general-purpose EM models first requires efficient sample generation methods. Figure 3a shows an efficient sample generation method based on MCES. The MCES architecture features a fixed structural backbone, specifically a double-layer cross-shaped metallic frame that functions as a series inductor (*L*_*I*_) and capacitor (*C*_*I*_), integrated with reconfigurable metacircuit components including diodes, resistors (*R*_*ij*_), inductors (*L*_*ij*_), and capacitors (*C*_*ij*_) as shown in Fig. 3a. This framework generates diverse electromagnetic functions through variations in diode states (presence/absence), component values (*R*_*ij*_, *L*_*ij*_, *C*_*ij*_), and substrate thickness, constituting what is termed the generalized design circuit for metasurfaces (GDCM). This design decouples wave interaction, which is handled by the passive frame, from wave manipulation, controlled by the integrated metacircuits, thereby enabling diverse functionality through metacircuit modifications alone. To parametrize this design space, a 14-dimensional vector was defined that encodes key variables including the state of diodes, values of metacircuit components such as resistors *R*_*ij*_, inductors *L*_*ij*_, and capacitors *C*_*ij*_, as well as substrate thickness *h*. Component values were sampled from practical ranges to ensure realizability. The electromagnetic response for each parameter set was characterized by its *S*_11_ and *S*_21_ parameters across a broad frequency range (0.1–40 GHz), yielding an 800-element output vector. Notably, the diodes have two operating states, namely off-state and on-state. Therefore, the first position of the GDCM needs to be replaced by the two bits {*R*_*on*_, 0}, {0, *C*_*off*_} and {0, 0} to describe the operating state and absence of the diode. Specifically, *R*_*on*_ and *C*_*off*_ represent the equivalent resistance and capacitance in the on-state and off-state of the diode, respectively. The combination {0, 0} indicates that the metasurface unit structure has no diode. Using this framework, we automated the generation of over 6 million parameter-response pairs via circuit simulations in Advanced Design System (ADS), controlled by a Python script. This approach achieved a data generation rate of ~0.29 seconds per sample, which is two orders of magnitude faster than full-wave electromagnetic simulations. This high efficiency makes the creation of such a large-scale dataset feasible, as demonstrated in Fig. 3 d1. Notably, the current sample generation time was achieved on a system with an AMD EPYC 7402 CPU at 2.79 GHz, completing in just several days a task that would typically require years with full-wave simulation under the same configuration. Furthermore, by leveraging parallel computing, it is feasible to generate billions or even more samples within a short time frame, offering a promising pathway to meet the massive data requirements for building general-purpose EM models (see Supplementary Information Section 3).

Leveraging the large-scale training samples, we developed a deep learning model to establish a mapping between the GDCM code of the MCES and its electromagnetic response. The fully connected network architecture comprises an input layer (15 nodes), an output layer (800 nodes, representing *S*_11_ and *S*_21_ spectra from 0.1 to 40 GHz), and four hidden layers (64-256-512-1024 neurons). Trained on 4.8 million samples (80% of the dataset) using a mean squared error (MSE) loss, the model achieved a final MSE of 1.59 × 10^−4^ after 1000 epochs, confirming its accurate learning of the underlying physics (Fig. 3d2). We further validated that the model’s accuracy scales with dataset size (Fig. 3d3) and, crucially, that it generalizes effectively to circuit parameters beyond the original training range, exhibiting only modest performance degradation even when component values were doubled (Fig. 3d4).

To validate the model’s practical utility, we evaluated its performance on three canonical metasurface types: FSS, ESS, and absorbers. As shown in Fig. 3d5–d7, the predicted S-parameters for all three classes show excellent agreement with full-wave simulations. The ESS response was accurately captured across both diode on- and off-states (MSE = 2.7 × 10^−5^), while the FSS and absorber responses were predicted with high fidelity (MSE = 3.4 × 10^−3^ and 4.9 × 10^−4^, respectively). The consistently low MSE values across these functionally distinct metasurfaces confirm the model’s robust predictive power and generalization capability. This accurate forward model serves as the critical foundation for the inverse design demonstrations that follow.

### Design and experiment

A key challenge in building general-purpose EM models is that current methods produce models which lack versatility for unified design across multiple metasurface types. To demonstrate the universal design capability of our MCES-based EM model, we designed and fabricated three representative metasurfaces. First, an FSS and an ESS were implemented using a lumped-element scheme to validate the model for passive and active metasurface designs, respectively. Then, to overcome the limitations of lumped components in complex circuit integration, we designed and fabricated the absorber using micro-nano technology and experimentally verified its performance.

**Fig. 4:**
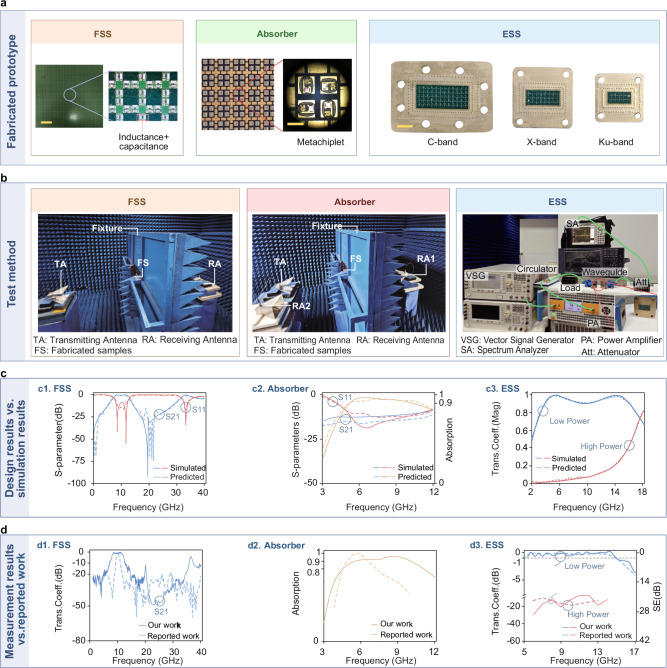
EM metasurface design and measurement results. **a** fabricated prototypes of the designed FSS, absorber, and ESS. Designs **a1, a3** use lumped components, while **a2** is based on a micro-nano process. **b** Photograph of test environment. **c** Comparison of inverse design results and full-wave simulation values. The advanced works of FSS, absorber, and ESS are used as the inverse design targets of the model, where FSS requires a 3 dB bandwidth greater than 12% and a 20 dB suppression bandwidth exceeding 13 GHz. ESS requires IL less than 1 dB and SE greater than 10 dB at 5.4–14.5 GHz. The bandwidth of the absorber with an absorption rate greater than 80% exceeds 55.6%. The resulting designs and their corresponding GDCM codes are: **c1** FSS (GDCM code: {001.10000.201.10000.25}), **c2** absorbers (GDCM code: {015010.20001000005}), and **c3** ESS (GDCM code: {10800000800004}). **d** Measured performance of inverse-designed **d1** FSS, **d2** absorber, and **d3** ESS compared to reported work. Our metasurface design matches advanced works, with comprehensive comparison data in Supplementary Information Section 4. It is worth noting that d3 represents the design and measurement results of MCES based on micro-nano technology. We implement the MCES paradigm using standard micro-nano processes, integrating sophisticated circuitry into a standalone, functional chip. This establishes a concrete physical realization of MCESs. Crucially, the external metasurface structure remains fixed; different electromagnetic functionalities are achieved solely by interchanging this programmable chiplet. Scale bars, 5 cm (FSS), 1 mm (absorber), 1 cm (ESS).

With the accurate forward model established, we next implement an inverse design strategy to discover MCES configurations that achieve user-specified electromagnetic responses. The process defines a target frequency response as the optimization objective and employs an algorithm to efficiently search the input parameter space for optimal circuit configurations. The pre-trained network enables rapid evaluation of each candidate design, making this search computationally feasible. The power of this approach is demonstrated through the design of three functionally distinct metasurfaces, including an FSS, an ESS, and an absorber, all with the design goal to surpass the performance metrics reported in the literature. For the FSS, the model produced a design with a −3 dB bandwidth increased to 54%, a substantial improvement over the reference value of 12%^52^. Similarly, the inversely designed absorber achieved an 80% absorption bandwidth of 86.9%, outperforming the reference structure^49^. Most impressively, for the ESS, the inverse design process identified a configuration requiring only a single diode and inductor (Fig. 4c), yet it achieved an ultra-wide operating band (3.7–15.1 GHz) with low insertion loss (IL < 1 dB) and shielding effectiveness (SE > 10 dB), significantly simplifying a previously complex three-layer design^53^. In all cases, the predicted responses of the final designs showed excellent agreement with full-wave simulations.

The MCES framework demonstrates decisive advantages over prior AI-driven metasurface design methods (Table 1). Unlike approaches reliant on structural parameter scanning or discrete geometric encoding, our circuit-driven method achieves a two-order-of-magnitude acceleration in data generation, facilitates million-sample datasets, and maintains high predictive accuracy. Consequently, it achieves inverse design generation times an order of magnitude faster than other methods. Most importantly, our model exhibits unprecedented capability for arbitrary amplitude manipulation across diverse metasurface functions, moving beyond the single-function or exclusive S_11_ modulation limitations of previous designs.

**Table 1:** Comparison between the proposed MCES and other reported works

Ref	Training set generation method	Sample size	MSE	Frequency coverage	Function
^38^	Topology encoding	20,000	1.7 × 10^−1^ Cross-entropy	1–12 GHz	Reflective surface
^44^	Topology encoding	2100	–	2.5–20 GHz	Absorber
^55^	Topology encoding	3000	1.1 × 10^−4^	1–12 GHz	ESS
^56^	Topology encoding	10,922	8 × 10^−3^	4–8 GHz	Reflective surface
^57^	Size adjustment	6000	8.4 × 10^−2^	2–40 GHz	FSS
^58^	Size adjustment	8000	5 × 10^−5^	1–18 GHz	Absorber
^59^	Size adjustment	11,000	1 × 10^−2^	333–750 THz	Absorber
^60^	Size adjustment	7200	7 × 10^−2^	2–15 GHz	Reflective surface
MCES	First proposed Circuit adjustment	6,072,216	1.6 × 10^−4^	0.1–40 GHz	Frequency amplitude polarization phase modulation

To demonstrate the practical viability of the proposed approach, we experimentally validated the performance of the inversely designed MCESs through prototype fabrication and testing. A 320 mm × 320 mm FSS prototype was fabricated. Measurement results confirm bandpass filtering functionality with transmission coefficient > −3 dB from 8–12.1 GHz and strong out-of-band rejection (<−10 dB from 0 to 7.1 GHz and 12.8 to 35.2 GHz). While the passband narrowed slightly compared to simulation, out-of-band suppression improved significantly. Specifically, the −10 dB bandwidth expanded from 21 GHz to 29.5 GHz, and the −20 dB bandwidth increased from 12.2 GHz to 20 GHz. These results represent a substantial improvement over the reference design^52^, with measured -3 dB bandwidth increasing to 41% (from 12%) and −20 dB stopband widening to 20 GHz (from 13 GHz), as shown in Fig. 4d1.

We designed and fabricated an absorber using standard micro-nano processes (see Supplementary Section 4). The device, shown in Fig. 4a2, incorporates a series *RLC* network (*R*_11_-*L*_11_-*C*_11_) in the top chip and a single resistor *R*_21_ in the bottom chip. Fig. 4d2 compares the design target with the measured results. The absorber achieves over 80% absorption from 4.5 to 11.6 GHz, with more than 90% absorption between 5.6 and 10.3 GHz. Compared to the design^49^, our micro-nano-based implementation achieves a significant improvement in absorption bandwidth (for >80% absorption) from 57% to 88.2%, while simultaneously reducing electrical size from 0.22*λ*_0_ to 0.1*λ*_0_ and increasing the cost-effectiveness of bandwidth (BW_CE_) from 166.78 to 713.3 (see Supplementary Information Section 4).

To cover the ultra-wideband operation from C- to Ku-band, we fabricated three ESS prototypes tailored to specific waveguide frequency ranges. Measurements confirmed excellent performance across 5.2–14.5 GHz, with IL below 1 dB in low-power conditions. Under high-power excitation, the devices exhibited strong nonlinear responses with SE reaching 27.25 dB at 6 GHz, 25.6 dB at 9 GHz, and 24.9 dB at 13 GHz. The measured *S*_21_ for both transmission and shielding states show good agreement with simulations and significantly advance beyond the performance reported in ref. ^53^, as shown in Fig. 4d3.

## Discussion

This work introduces a generalizable framework that transforms the metasurface design paradigm through a standardized language of fixed topology and configurable integrated circuits. The MCES framework delivers two key breakthroughs. First, it accelerates training data generation by two orders of magnitude via circuit-level modeling, creating previously unattainable large-scale datasets to solve the critical data scarcity problem in general-purpose EM models development. Second, it establishes a unified AI-driven design methodology that overcomes the limitations of EM model construction efficiency by supporting diverse metasurface functions within a single framework, validated through three distinct metasurface classes with performance matching or surpassing excellent designs. Crucially, we demonstrate a micro-nano-based implementation that translates the MCES concept into a practical reality, enabling direct mapping from circuit schematics to electromagnetic functionality while bypassing traditional optimization cycles. By providing a scalable framework, this work significantly shortens the development cycle for AI-driven metasurface design while delivering unprecedented functional diversity, thereby paving the way for future large-scale electromagnetic models.

## Methods

### Training data generation

The training dataset for the MCES framework was generated using a field-circuit co-simulation approach in ADS (Advanced Design System). First, the S-parameters of the fixed metasurface topology (e.g., the double-layer cross-shaped metallic structure) were extracted through a single full-wave simulation in CST Microwave Studio. These S-parameters fully capture the electromagnetic behavior of the passive frame, including its frequency-dependent transmission, reflection, and loss characteristics. Subsequently, the extracted S-parameters were imported into ADS as a frequency-dependent component and co-simulated with lumped-element circuits comprising resistors (R), inductors (L), capacitors (C), and diodes. This field-circuit co-simulation approach ensures that the generated data inherently account for the full-wave electromagnetic behavior of the fixed structure while maintaining the computational efficiency required for large-scale dataset generation.

Traditional EM modeling can indeed obtain large-scale datasets with various performance metrics via full-wave simulations of numerous structures. However, this approach requires access to high-performance computing servers and involves prohibitively long simulation times. Generating millions of samples using conventional methods would take years of computation, making such large-scale data generation practically infeasible for most research groups.

The key advantage of the MCES framework lies not in enabling large-scale data generation, but in achieving this with dramatically improved efficiency. By reducing the simulation time per sample from minutes to milliseconds, MCES can generate millions of samples in days on a consumer-grade laptop. This represents an improvement of approximately two orders of magnitude compared to conventional approaches and democratizes access to large-scale data-driven design for researchers without access to supercomputing resources.

### Micro-nano fabrication considerations

The MCES framework can be implemented through two pathways: PCB-based lumped elements for rapid prototyping and micro-nano integration (termed “metachiplet”) for high-frequency applications. Micro-nano fabrication minimizes parasitic effects through extreme miniaturization, precise dimensional control, monolithic integration, and advanced materials. A detailed analysis of these mechanisms, along with quantitative comparisons with conventional PCB processes, is provided in Supplementary Information Section 4.

### Cost comparison between micro-nano fabrication and conventional methods

A detailed cost analysis of the micro-nano fabrication process, including component-level cost breakdown, comparison with discrete alternatives, and full-workflow economic assessment, is provided in Supplementary Information Section 4.

### Inverse design optimization

For inverse design, we use Particle Swarm Optimization (PSO) to search the circuit parameter space. The objective function minimizes the mean squared error between the target response and the prediction from the trained forward network. See Supplementary Information Section 3 for implementation details, algorithm selection, and comparison with other methods.

### Design and validation workflow for component modeling

The trained model first generates ideal RLC parameters for a target response. Real components with the closest available values are then selected based on manufacturer datasheets. Finally, S-parameter models of these components are imported into a full-wave simulator to validate the design under realistic conditions. This three-stage approach ensures that designs benefit from the efficiency of ideal models during training while being rigorously validated against real component behavior.

### Samples fabrication

As a demonstration, we fabricated FSS, absorber and ESS prototypes using standard printed circuit board (PCB) technology. Fig. 4a1 presents the fabricated FSS prototypes. Each array consists of 114  × 114 unit cells with a 2.8 mm periodicity, resulting in a total prototype size of 320 mm  × 320 mm. These structures were similarly implemented on Rogers 5880 substrates. Fig. 4a2 shows the micro-nano-fabricated absorber prototype. This design comprises 10  × 10 unit cells (4 mm periodicity). Unlike the lumped-element approach, this prototype incorporates integrated circuits fabricated using micro-nano processes. These circuits, termed “metachiplet”, each contain a series R-L-C network and measure 1 mm × 1 mm. The metasurface functionality is determined by the specific metacircuit integrated within each metachiplet, whereas other functionalities require different metacircuit designs. Fig. 4a3 shows photographs of the completed ESS prototypes. To cover the operational bandwidth spanning C-, X-, and Ku-bands, we fabricated three ESS samples with dimensions corresponding to standard waveguide sizes: 34.85 mm × 15.80 mm (WR137), 22.86 mm × 10.16 mm (WR90), and 15.80 mm × 7.90 mm (WR62). Both the top copper patterns and bottom ground planes were fabricated on commercial Rogers 5880 substrates (*ϵ*_*r*_ = 2.2, $\tan \delta=0.001$).

### Measurement system

To validate the performance of the designed FSS, ESS, and absorber, we established dedicated experimental setups for each component.

FSS measurements. The FSS prototype was characterized using a free-space measurement system in a microwave anechoic chamber (Fig. 4b1). The sample was mounted within a 1 m × 1 m absorptive screen featuring a rectangular aperture matching the FSS dimensions. Two horn antennas, connected to a VNA, were aligned on opposite sides of the screen. Three antenna pairs covering distinct frequency bands were employed to span the full measurement range. The experimental setup follows established methodologies^54^.

Absorber measurements. Scattering parameter measurements were performed using a Keysight PNA (8361C) vector network analyzer with the experimental configuration shown in Fig. 4b2. Three standard-gain horn antennas were connected to the VNA ports, consisting of one transmitter (Tx) and two receivers (Rx1, Rx2). Tapered wedge absorbers surrounded the device under test (DUT) to minimize environmental reflections. The Tx antenna illuminated the absorber prototype with normally incident waves. Reflection coefficients were measured using Rx2 with the absorber backed by a metal ground plane, while transmission coefficients were acquired using Rx1 without the ground plane. These measurements enabled experimental validation of absorption performance across the operating bandwidth. The methodology follows established procedures for electromagnetic metamaterial characterization.

ESS insertion loss and shielding effectiveness characterization. IL measurements under low-intensity radiation fields (LIRF) were conducted using a vector network analyzer (VNA) connected to standard waveguide ports. The output power was maintained below −25 dBm to ensure diode-off conditions. The corresponding electric field intensity at the waveguide center was calculated as 5.2 V/m at 10 GHz based on TE_10_ mode field distributions, confirmed to be below the diode activation threshold. Measurements across three waveguide bands (C/X/Ku) are shown in Fig. 4b3. For high-intensity radiation field (HIRF) testing, we established a setup comprising a vector signal generator (VSG), power amplifier (PA), and spectrum analyzer. A circulator with terminated load protected the PA from reflected power. System losses were calibrated prior to measurements to ensure accurate power quantification. SE was calculated as SE(dB) = *P*_in_(dBm) − *P*_out_(dBm), where *P*_in_ and *P*_out_ represent input and output power, respectively. Nonlinear response characterization was performed by sweeping incident power from −10 dBm to maximum amplifier output across C/X/Ku bands, with results presented in Fig. 4d3.

## Supplementary information

Supplementary Information

Transparent Peer Review file

## Data Availability

The source code and datasets generated and analyzed during the current study have been deposited in the Zenodo repository under the accession code 10.5281/zenodo.19688825. The data will be made publicly available upon publication of the manuscript. Source data are provided with this paper.

## Code availability

The source code generated during this study has been deposited in Zenodo under the accession code 10.5281/zenodo.19688825and will be made publicly available upon publication.
