# Supplementary material for: Large-scale data-driven inverse EM design based on metacircuit-embedded surface
Source: Nat Commun. 2026 Jul 13;17:8603. doi: 10.1038/s41467-026-75411-z (PMC13486762; doi:10.1038/s41467-026-75411-z)
Supplement: Supplementary file 1 [file 41467_2026_75411_MOESM1_ESM.pdf]

Supplementary Information for

Large-Scale Data-Driven Inverse EM Design Based on Metacircuit-Embedded Surface

Ming Xu[1,†], Song Zha[1,†,*], Mingtuan Lin[1,†,*], Hanqing Liu[1] , Jihong Zhang[1] , Yanlin Xu[1] , Huan Jiang[1] , Qiang Cheng[2,*] , Peiguo Liu[1,*]

[1] *College of Electronic Science and Technology, National University of Defense Technology , 109 Deya Road , Changsha , 410073 , Hunan, China*
[2] *State Key Laboratory of Millimeter Waves, Southeast University , Nanjing, 214135 , Jiangsu, China*

[†] These authors contributed equally to this work
[*]Author to whom correspondence should be addressed. E-mail: zhasong@nut.edu.cn
[*]Author to whom correspondence should be addressed. E-mail: linmingtuan08@nudt.edu.cn
[*]Author to whom correspondence should be addressed. E-mail: qiangcheng@seu.edu.cn.
[*]Author to whom correspondence should be addressed. E-mail: pgliu@nudt.edu.cn

Contents

List of Figures **3**

List of Tables **4**

1 Supplementary Note 1. Metacircuit-embedded surface. **5**

2 Supplementary Note 2. Demonstrating MCES for diverse metasurface designs. **7**
- 2.1 High functionality of MCES designed metasurfaces. 7
  - 2.1.1 Validation of MCES for Frequency-Domain Design of Metasurfaces 7
  - 2.1.2 Validation of MCES for Amplitude-Domain Design of Metasurfaces 9
  - 2.1.3 Validation of MCES for Polarization-Domain Design of Metasurfaces 10
  - 2.1.4 Validation of MCES for Phase-Domain Design of Metasurfaces 11
- 2.2 High performance of MCES designed metasurfaces. 11
  - 2.2.1 FSS Design Based on MCES. 11
  - 2.2.2 ESS Design Based on MCES. 14
  - 2.2.3 Absorber Design Based on MCES. 16
  - 2.2.4 Multi functional metasurface based on MCES. 17

3 Supplementary Note 3. Artificial Intelligence Assisted MCES Metasurface Design **18**
- 3.1 MCES enables standardized and unified metasurface design through implementation of the GDCM. 18
- 3.2 MCES achieves two-order-of-magnitude higher data generation efficiency than conventional approaches. 22
- 3.3 Large training sample size enhances model accuracy and generalization capability. . . . 23
- 3.4 Leveraging the large-scale datasets generated by MCES, we achieve rapid design of diverse metasurfaces through classical optimization algorithms. 23
- 3.5 Optimization Algorithm for Inverse Design 25
- 3.6 Dataset Size Justification Analysis 26

4 Supplementary Note 4. Experimental verification of MCES **27**
- 4.1 Experimental verification of ESS based on MCES. 27
- 4.2 Experimental verification of FSS based on MCES. 31
- 4.3 Experimental verification of absorber based on MCES. 32
- 4.4 MCES enables standardized design of metasurfaces via micro-nano fabrication processes. 33
- 4.5 Micro-Nano Fabrication for Parasitic Effect Minimization. 35
- 4.6 Cost Comparison between Micro-Nano Fabrication and Conventional Methods 36

5 Supplementary References **37**

List of Figures

1 The difference between MCES concept and traditional metasurface design. 5
2 MCES-based design of metasurfaces in the frequency domain. 6
3 MCES-based design of metasurfaces in the amplitude domain. 8
4 MCES-based design of metasurfaces in the polarization and phase domain. 10
5 MCES-based FSS design using previously reported FSS structures as design targets. . 12
6 MCES-based ESS design using previously reported ESS structures as design targets . . 14
7 MCES-based absorber design using previously reported absorber structures as design targets. 16
8 MCES-based absorptive frequency-selective reflector design. 17
9 MCES-based absorptive energy-selective reflector design. 18
10 Generalizable design circuit for metasurfaces. 20
11 The advantage of a large training sample size. 22
12 Metasurface inverse design results. 24
13 Validation MSE versus dataset size. (a) Validation MSE on a logarithmic scale. (b) Validation MSE in linear scale. 26
14 Predicted versus simulated responses for an out-of-sample configuration using models trained with different dataset sizes. 28
15 Measurement of ESS. (a) ECM of the ESS proposed based on UDCM. (b)Experimental setup for HIRF. (c)Comparison between predicted and simulated performance of the inversely designed ESS(periodic boundary). (d)Transmission coefficients of ESS in ADS and CST. (e) Transmission coefficient measurement results of the ESS under LIRF and HIRF. (f)SE and transmission coefficient measurement results of the ESS under different injection power. Created in BioRender. XU, M. (2026) https://BioRender.com/dv6dbgo. 29
16 Measurement of FSS. 31
17 Measurement of absorber. 32
18 Design and measurement results of MCES based on micro-nano technology. 34

List of Tables

1 MCES-BASED DESIGN OF FREQUENCY-SELECTIVE SURFACES 7
2 MCES-BASED DESIGN OF ENERGY SELECTIVE SURFACES AND ABSORBERS 9
3 MCES-BASED DESIGN OF POLARIZATION CONVERTER 10
4 MCES-BASED DESIGN OF REFLECTION PHASE MODULATION 11
5 MCES-BASED DESIGN OF TRANSMISSION PHASE MODULATION 11
6 COMPARISON BETWEEN THE PROPOSED FSS AND OTHER REPORTED WORK 13
7 COMPARISON BETWEEN THE PROPOSED ESS AND OTHER REPORTED WORK 15
8 COMPARISON BETWEEN THE PROPOSED ABSORBER AND OTHER REPORTED WORK 17
9 COMPARATIVE PERFORMANCE OF GLOBAL OPTIMIZATION ALGORITHMS FOR METASURFACE INVERSE DESIGN (30 INDEPENDENT RUNS) 25
10 VALIDATION MSE FOR MODELS TRAINED WITH DIFFERENT DATASET SIZES (600 EPOCHS) 26
11 OUT-OF-SAMPLE MSE FOR MODELS TRAINED WITH DIFFERENT DATASET SIZES 27
12 COMPARISON BETWEEN THE PROPOSED ESS AND OTHER REPORTED WORK 30
13 COMPARISON BETWEEN THE PROPOSED FSS AND OTHER REPORTED WORK 31
14 COMPARISON BETWEEN THE PROPOSED ABSORBER AND OTHER REPORTED WORK 33

Supplementary Note 1. Metacircuit-embedded surface.

To overcome the challenges of data scarcity and inefficient methodology in building electromagnetic large model, we propose the "Metacircuit-Embedded Surface (MCES)". This framework adopts a "simple structure + complex circuits" strategy: the fundamental metasurface unit maintains a fixed geometry, while various lumped components—resistors (R), inductors (L), capacitors (C), and diodes—are integrated into its gaps. By embedding diverse circuit configurations, MCES can design multiple electromagnetic functions.

The theoretical foundation of MCES stems from the "equivalent circuit model", a classical approach in metasurface design. In theory, each electromagnetic metasurface can be represented as an equivalent circuit, where functional diversity arises from variations in the circuit topology. In MCES, the core electromagnetic structure remains unchanged, while the integrated circuits introduce complexity and versatility, enabling the design of various metasurfaces. Consequently, MCES provides a "unified design paradigm" that encompasses various metasurface configurations, offering a systematic and scalable methodology for advanced electromagnetic wave manipulation.

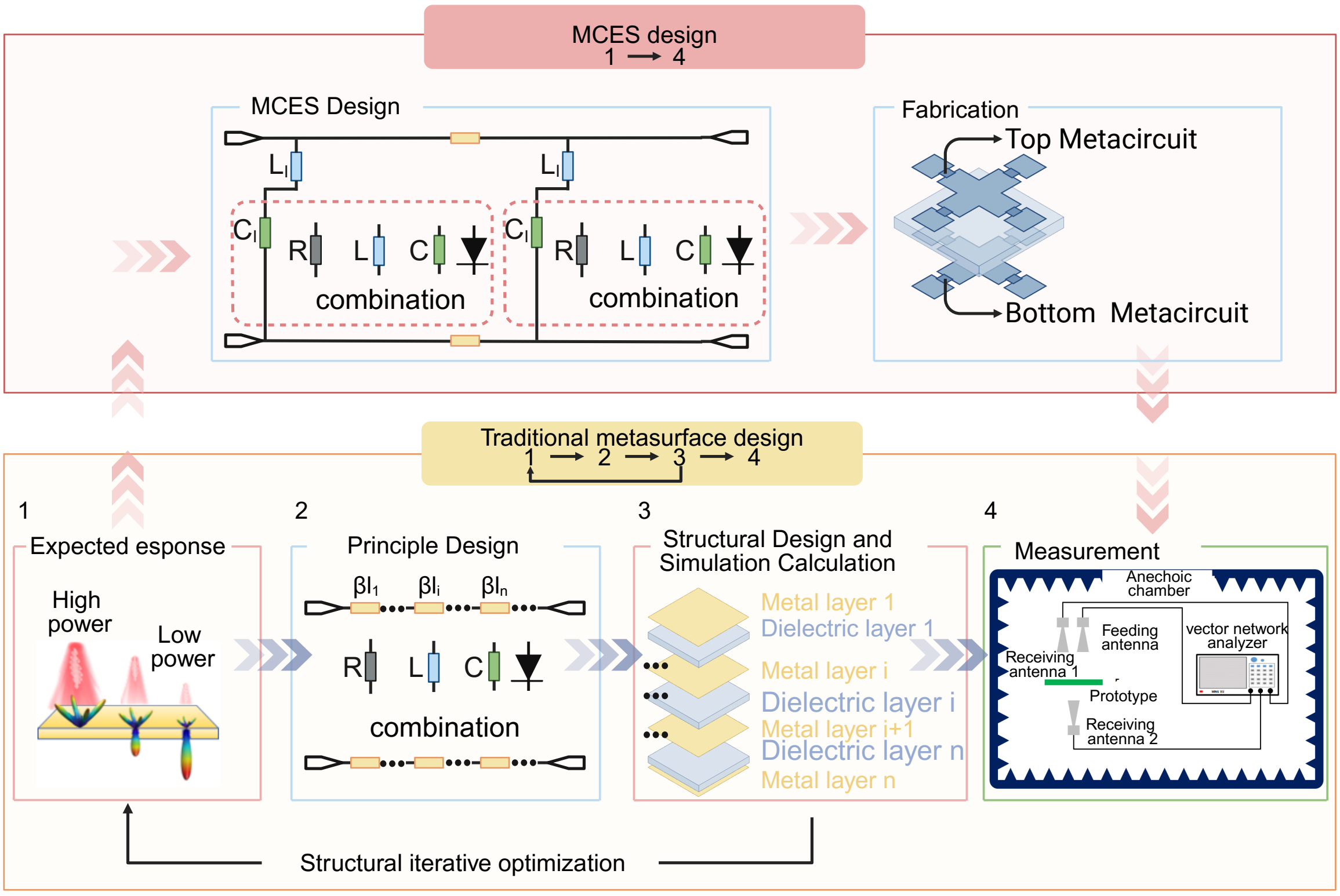

Supplementary Fig. 1: The difference between MCES concept and traditional metasurface design. Traditional metasurface design relies on principle-driven methods, where equivalent circuit models guide the development of unit cells that then require iterative structural optimization and full-wave simulations to meet theoretical targets—a process that is both computationally intensive and functionally specific. In contrast, our MCES-based metasurface inverts this workflow. It uses a simple, fixed geometric scaffold (e.g., a cross structure) whose electromagnetic response is determined by integrated circuits—such as resistors, capacitors, or diodes—designed and optimized rapidly using circuit simulators like ADS, bypassing the need for full-wave simulation loops. This approach decouples structural and functional design, enabling swift reconfiguration of performance through circuit adjustments alone. Created in BioRender. XU, M. (2026) https://BioRender.com/i55ofh2.

As shown in Supplementary Figure 1, conventional metasurface design necessitates a principle-driven approach to achieve the desired frequency or phase response, primarily relying on equivalent circuit modeling, followed by deriving an approximate unit-cell structure from the circuit. Subsequent iterative optimization of structural parameters is required to align the response with theoretical expectations, followed by experimental verification. Crucially, this traditional paradigm hinges on

ad hoc principle design—often non-generalizable across different functional targets—while structural optimization dominates the computational burden due to its iterative nature.

In stark contrast, the proposed MCES inverts this workflow: its foundational unit (e.g., a cross or other simple geometric shape) exhibits exceptional structural simplicity, enabling direct modulation of electromagnetic responses solely through integrated circuit modifications (e.g., resistors, inductors, capacitors, or diodes). These components can be rapidly prototyped and evaluated via circuit simulators like ADS without requiring full-wave simulations. This bypasses the conventional full wave simulation-optimization loop, collapsing the design pipeline from schematic to experimental verification. In contrast to conventional metasurface designs that predominantly focus on unit cell structural engineering, the proposed MCES maintains an elementary structural configuration while incorporating integrated circuitry, enabling rapid functional reconfiguration through circuit modifications and thereby introducing a new design dimension to metasurface development. To showcase the potential

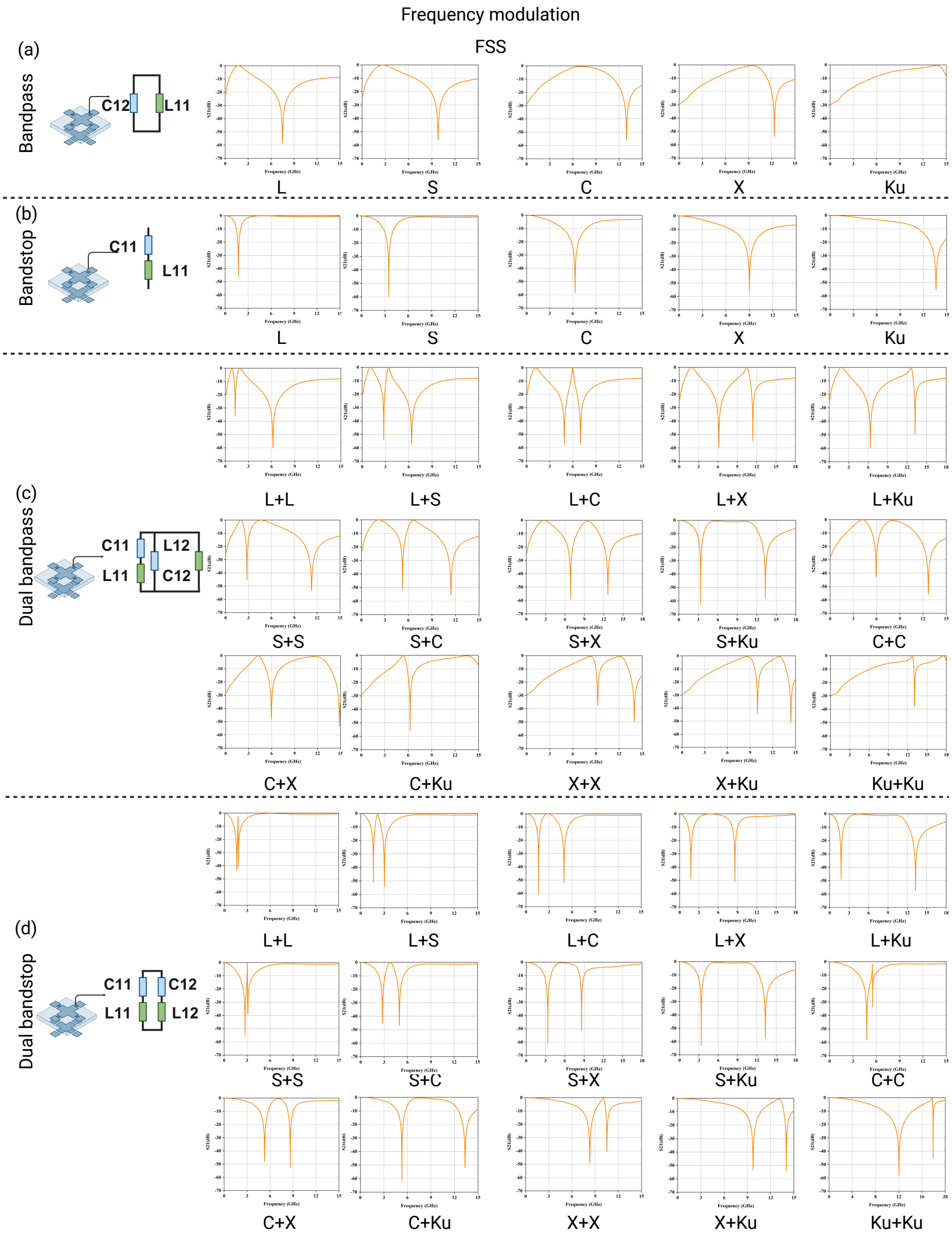

Supplementary Fig. 2: MCES-based design of metasurfaces in the frequency domain. (a) MCES-designed bandpass FSSs covering the L- to Ku-band. (b) MCES-designed band-stop FSSs covering the L- to Ku-band. (c) MCES-designed dual-band bandpass FSSs covering the L- to Ku-band. (d) MCES-designed dual-band bandstop FSSs covering the L- to Ku-band. Created in BioRender. XU, M. (2026) https://BioRender.com/yfpmjt5.

of MCES in metasurface applications, we introduced its implementation in the frequency, amplitude, phase, and polarization domains, as well as in multi-domain joint designs and multifunctional metasurfaces. This illustrates the versatility of MCES in manipulating electromagnetic waves.

Supplementary Note 2. Demonstrating MCES for diverse metasurface designs.

To validate the capability of MCES for practical metasurface design, this work presents its implementation in the frequency, amplitude, polarization, and phase domains as representative examples, highlighting the versatile functionality and high performance of MCES in metasurface design.

2.1 High functionality of MCES designed metasurfaces.

This section demonstrates the high functionality of MCES in metasurface design through an extensive set of 280 designs, encompassing the frequency, amplitude, polarization, and phase domains.

2.1.1 Validation of MCES for Frequency-Domain Design of Metasurfaces

Frequency-selective surface (FSS) represents the most widely used class of metasurfaces for frequency-dimensional modulation. They allow electromagnetic waves to pass with low insertion loss (IL) within a designated frequency band while attenuating waves outside that band. To verify the applicability of MCES to frequency-domain design, this study employed MCES to design bandpass, bandstop, dual-band bandpass, and dual-bandstop metasurfaces operating across the L- to Ku-band range, thereby covering the principal FSS design types. The corresponding designs are presented in Supplementary Figure 2.

Supplementary Table 1: MCES-BASED DESIGN OF FREQUENCY-SELECTIVE SURFACES

Type	Frequency band	Diode	L_{11} / nH	C_{11} / pF	L_{12} / nH	C_{12} / pF	h / mm
Bandpass	L	N	8	–	–	1	4
Bandpass	S	N	5	–	–	0.6	4
Bandpass	C	N	0.8	–	–	0.5	4
Bandpass	X	N	0.3	–	–	0.9	4
Bandpass	Ku	N	0.2	–	–	0.7	4
Bandstop	L	N	8	1	–	–	4
Bandstop	S	N	3	0.6	–		4
Bandstop	C	N	0.8	0.5	–	–	4
Bandstop	X	N	0.3	0.4	–	–	4
Bandstop	Ku	N	0.2	0.2	–	–	4
Dual Bandpass	L+L	N	15	1	10	1.5	4
Dual Bandpass	L+S	N	6	0.5	10	1.5	4
Dual Bandpass	L+C	N	4	0.2	10	1.5	4
Dual Bandpass	L+X	N	2	0.2	5	1	4
Dual Bandpass	L+Ku	N	1	0.3	5	1	4
Dual Bandpass	S+S	N	6	0.5	4	0.5	4
Dual Bandpass	S+C	N	4	0.2	6	0.5	4
Dual Bandpass	S+X	N	2	0.2	4	0.5	4
Dual Bandpass	S+Ku	N	1	0.2	4	0.5	4
Dual Bandpass	C+C	N	3.1	0.2	1.5	0.5	4
Dual Bandpass	C+X	N	1	0.5	1	0.5	4
Dual Bandpass	C+Ku	N	1	0.5	0.5	0.2	4
Dual Bandpass	X+X	N	2.6	0.1	0.3	0.8	4
Dual Bandpass	X+Ku	N	2.1	0.1	0.3	0.8	4
Dual Bandpass	Ku+Ku	N	1.3	0.1	0.1	1	4
Dual Bandstop	L+L	N	9	0.8	9	1	4
Dual Bandstop	L+S	N	3	0.8	9	1	4
Dual Bandstop	L+C	N	3	0.3	9	1	4
Dual Bandstop	L+X	N	3	0.1	5	1.5	4
Dual Bandstop	L+Ku	N	1	0.1	5	1.5	4
Dual Bandstop	S+S	N	3	0.8	6	0.5	4
Dual Bandstop	S+C	N	3	0.3	6	0.5	4
Dual Bandstop	S+X	N	3	0.1	4	0.5	4
Dual Bandstop	S+Ku	N	1	0.1	4	0.5	4
Dual Bandstop	C+C	N	3	0.3	4	0.2	4
Dual Bandstop	C+X	N	3	0.1	4	0.2	4
Dual Bandstop	C+Ku	N	1	0.1	4	0.2	4
Dual Bandstop	X+X	N	3	0.1	2	0.1	4
Dual Bandstop	X+Ku	N	1	0.1	2	0.1	4
Dual Bandstop	Ku+Ku	N	0.9	0.1	0.7	0.1	1

Specifically, Supplementary Figure 2(a) and (b) show, respectively, the bandpass and bandstop FSSs designed using MCES, both covering the L- to Ku-band spectrum.Supplementary Figure 2(c) and (d) depict the dual-band bandpass and dual-band bandstop FSSs realized via MCES. Their dual-band operating ranges cover the L- to Ku-bands and combinations thereof. For instance, the designed L-band dual-band bandpass FSS exhibits two passbands formed by combining the L-band with one of the L-, S-, C-, X-, or Ku-bands. Detailed design parameters for each metasurface are provided in Supplementary Table 1.

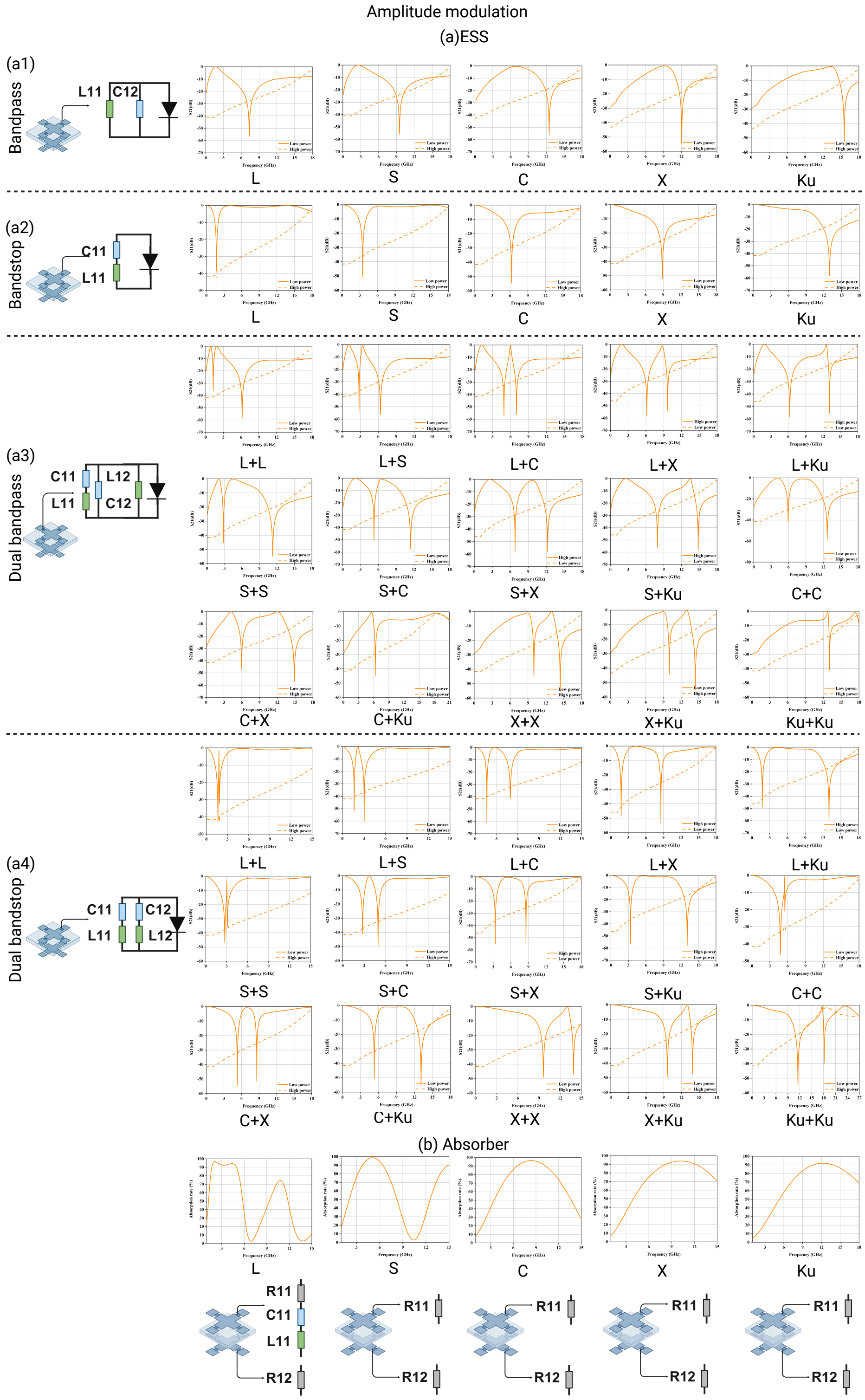

Supplementary Fig. 3: MCES-based design of metasurfaces in the amplitude domain. (a) MCES-designed ESSs covering the L- to Ku-band. (b) MCES-designed absorber covering the L- to Ku-band. Created in BioRender. XU, M. (2026) https://BioRender.com/c85flqx.

Supplementary Table 2: MCES-BASED DESIGN OF ENERGY SELECTIVE SURFACES AND ABSORBERS

**Type**	**Frequency band**	**Diode**	R_{11} Ω	L_{11} **nH**	C_{11} **pF**	R_{12} Ω	L_{12} **nH**	C_{12} **pF**	**h mm**
Bandpass ESS	L	Y	–	8	–	–	–	1	4
Bandpass ESS	S	Y	–	5	–	–	–	0.6	4
Bandpass ESS	C	Y	–	0.8	–	–	–	0.5	4
Bandpass ESS	X	Y	–	0.3	–	–	–	0.9	4
Bandpass ESS	Ku	Y	–	0.2	–	–	–	0.7	4
Bandstop ESS	L	Y	–	8	1	–	–	–	4
Bandstop ESS	S	Y	–	3	0.6	–	–	–	4
Bandstop ESS	C	Y	–	0.8	0.5	–	–	–	4
Bandstop ESS	X	Y	–	0.3	0.4	–	–	–	4
Bandstop ESS	Ku	Y	–	0.2	0.2	–	–	–	4
Dual Bandpass ESS	L+L	Y	–	15	1	–	10	1.5	4
Dual Bandpass ESS	L+S	Y	–	6	0.5	–	10	1.5	4
Dual Bandpass ESS	L+C	Y	–	4	0.2	–	10	1.5	4
Dual Bandpass ESS	L+X	Y	–	2	0.2	–	5	1	4
Dual Bandpass ESS	L+Ku	Y	–	1	0.3	–	5	1	4
Dual Bandpass ESS	S+S	Y	–	6	0.5	–	4	0.5	4
Dual Bandpass ESS	S+C	Y	–	4	0.2	–	6	0.5	4
Dual Bandpass ESS	S+X	Y	–	2	0.2	–	4	0.5	4
Dual Bandpass ESS	S+Ku	Y	–	1	0.2	–	4	0.5	4
Dual Bandpass ESS	C+C	Y	–	3.1	0.2	–	1.5	0.5	4
Dual Bandpass ESS	C+X	Y	–	1	0.5	–	1	0.5	4
Dual Bandpass ESS	C+Ku	Y	–	1	0.5	–	0.5	0.2	4
Dual Bandpass ESS	X+X	Y	–	2.6	0.1	–	0.3	0.8	4
Dual Bandpass ESS	X+Ku	Y	–	2.1	0.1	–	0.3	0.8	4
Dual Bandpass ESS	Ku+Ku	Y	–	1.3	0.1	–	0.1	1	4
Dual Bandstop ESS	L+L	Y	–	9	0.8	–	9	1	4
Dual Bandstop ESS	L+S	Y	–	3	0.8	–	9	1	4
Dual Bandstop ESS	L+C	Y	–	3	0.3	–	9	1	4
Dual Bandstop ESS	L+X	Y	–	3	0.1	–	5	1.5	4
Dual Bandstop ESS	L+Ku	Y	–	1	0.1	–	5	1.5	4
Dual Bandstop ESS	S+S	Y	–	3	0.8	–	6	0.5	4
Dual Bandstop ESS	S+C	Y	–	3	0.3	–	6	0.5	4
Dual Bandstop ESS	S+X	Y	–	3	0.1	–	4	0.5	4
Dual Bandstop ESS	S+Ku	Y	–	1	0.1	–	4	0.5	4
Dual Bandstop ESS	C+C	Y	–	3	0.3	–	4	0.2	4
Dual Bandstop ESS	C+X	Y	–	3	0.1	–	4	0.2	4
Dual Bandstop ESS	C+Ku	Y	–	1	0.1	–	4	0.2	4
Dual Bandstop ESS	X+X	Y	–	3	0.1	–	2	0.1	4
Dual Bandstop ESS	X+Ku	Y	–	1	0.1	–	2	0.1	4
Dual Bandstop ESS	Ku+Ku	Y	–	0.9	0.1	–	0.7	0.1	1
Absorber	L	N	110	3	0.7	1	–	–	14
Absorber	S	N	180	–	–	1	–	–	9
Absorber	C	N	180	–	–	1	–	–	5
Absorber	X	N	150	–	–	1	–	–	4
Absorber	Ku	N	180	–	–	1	–	–	3

2.1.2 Validation of MCES for Amplitude-Domain Design of Metasurfaces

To demonstrate the applicability of MCES to amplitude-modulated metasurface design, this study employed MCES to design bandpass, bandstop, dual-band bandpass, and dual-band bandstop energy-selective surface (ESS) covering the L- to Ku-band range, thereby encompassing the primary ESS design categories. Concurrently, absorbers operating across L- to Ku-band spectrum were designed. The corresponding designs are presented in Supplementary Figure 3.

Specifically, Supplementary Figure 3(a1) and (a2) depict the MCES-designed bandpass and bandstop ESS, respectively, both operating from L- to Ku- band. Supplementary Figure 3(a3) shows a dual-band bandpass ESS designed using MCES, whose two operational bands span the L- to Ku-band range and their combinations. Supplementary Figure 3(a4) presents a dual-band bandstop ESS realized via MCES, also featuring a dual-band operational range covering the L- to Ku-bands and their combinations. Finally, Supplementary Figure 3(b) illustrates an MCES-designed absorber operating across the L- to Ku-band. Detailed design parameters for each metasurface are provided in Supplementary Table 2.

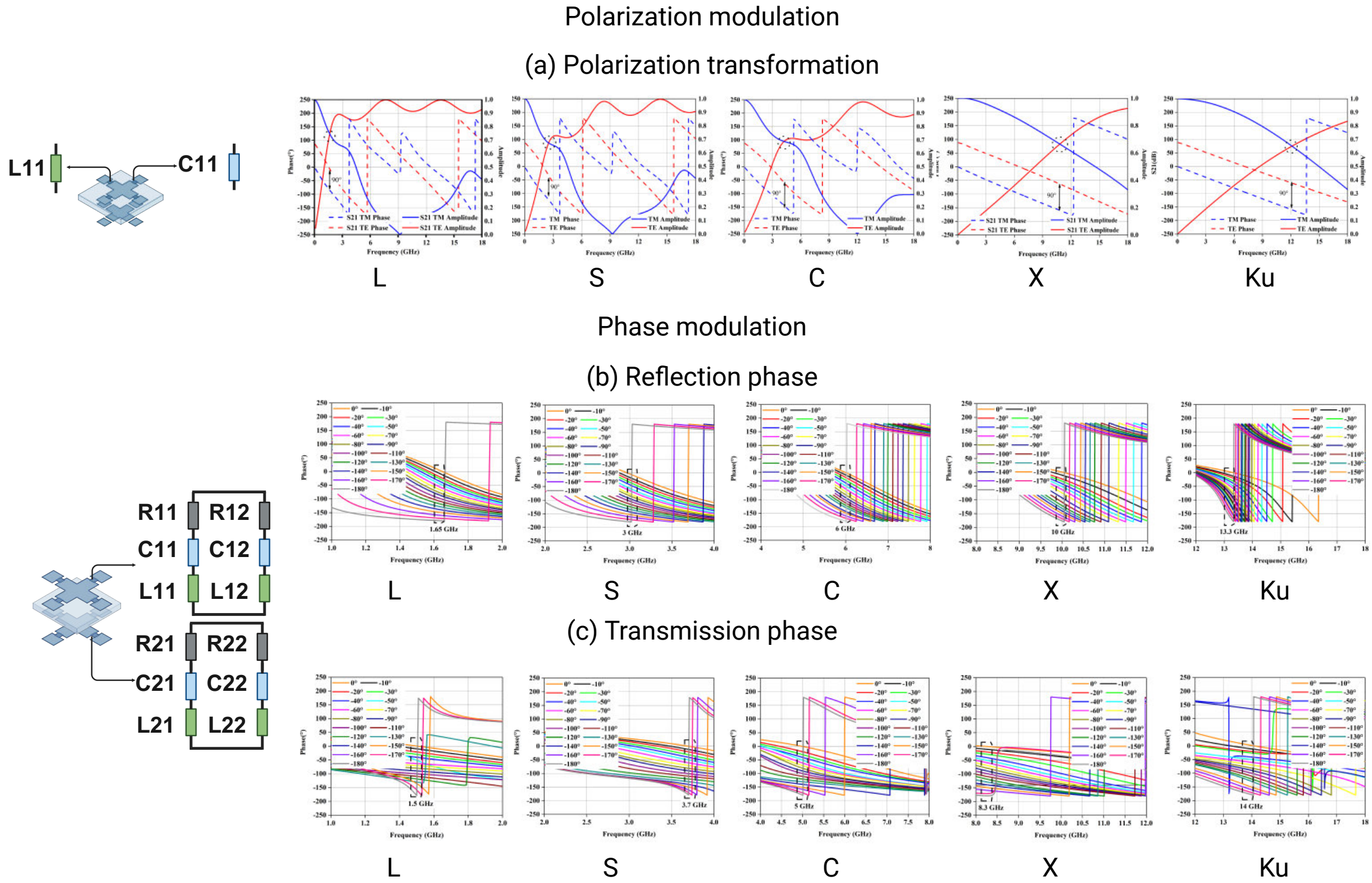

Supplementary Fig. 4: MCES-based design of metasurfaces in the polarization and phase domain. (a) MCES-designed polarization transformation surfaces covering the L- to Ku-band. (b) MCES-designed reflectors covering the L- to Ku-band, capable of controlling the reflection phase. (c) MCES-designed transmitters covering the L- to Ku-band, capable of controlling the transmission phase. Created in BioRender. XU, M. (2026) https://BioRender.com/3aunc0h.

2.1.3 Validation of MCES for Polarization-Domain Design of Metasurfaces

To validate the potential of MCES for polarization control, this study designed a linear-to-circular polarization converter operating across the L- to Ku-band using the MCES framework, as shown in Supplementary Figure 4(a). A linear-to-circular polarization converter is characterized by equal amplitudes in the two orthogonal polarization components of the transmitted wave, along with a phase difference of ±90° between them, when illuminated by a linearly polarized incident wave. The sign of the phase difference determines the handedness of the resulting circular polarization (left- or right-handed).

Supplementary Figure 4(a) shows the MCES-designed linear-to-circular polarization converter, which operates from L- to Ku-band. Representative frequency points within each band confirm that the transmitted wave exhibits equal amplitudes in the horizontal and vertical polarization components with a 90° phase difference. These results successfully demonstrate the applicability of MCES to polarization-domain metasurface design. Detailed design parameters for each metasurface are provided in Supplementary Table 3.

Supplementary Table 3: MCES-BASED DESIGN OF POLARIZATION CONVERTER

Frequency band	Frequency point / GHz	L_{11} / nH	C_{11} / pF	h / mm
L	1.6	6.6	0.7	14
S	2.5	3	0.7	14
C	4.2	1.7	0.4	9
X	10.7	0.4	0.1	1
Ku	12.1	0.5	0.1	0.1

2.1.4 Validation of MCES for Phase-Domain Design of Metasurfaces

To demonstrate the potential of MCES for phase modulation, this study designed reflective and transmissive phase modulators operating across the L- to Ku-band using the MCES framework. The designs are presented in Supplementary Figure 4(b) and (c). A phase modulator enables controlled variation of the phase of reflected or transmitted waves.

As shown in Supplementary Figure 4(b), the MCES-designed phase modulator operates from L- to Ku-band. Representative frequency points within each band confirm that the phase of the reflected or transmitted wave can be precisely controlled in 10° steps over a range of 0° to -180°. These results successfully validate the application of MCES in the phase-domain design of metasurfaces. Detailed design parameters for each metasurface are provided in Supplementary Table 4 and Supplementary Table 5.

Supplementary Table 4: MCES-BASED DESIGN OF REFLECTION PHASE MODULATION

**Frequency band**	R_{11} Ω	L_{11} **nH**	C_{11} **pF**	R_{12} Ω	L_{12} **nH**	C_{12} **pF**	R_{21} Ω	L_{21} **nH**	C_{21} **pF**	R_{22} Ω	L_{22} **nH**	C_{22} **pF**	**h mm**
L	–	–	0.7 to 3.8	–	–	–	1	–	–	–	–	–	14
S	–	–	0.2 to 1.2	–	–	–	1	–	–	–	–	–	9
C	–	–	0.1 to 0.4	–	–	–	1	–	–	–	–	–	3
X	–	–	0.1 to 0.2	–	–	–	1	–	–	–	–	–	1
Ku	–	–	0.1 to 0.2	–	–	–	1	–	–	–	–	–	0.254

Supplementary Table 5: MCES-BASED DESIGN OF TRANSMISSION PHASE MODULATION

**Frequency band**	L_{11} **nH**	C_{11} **pF**	L_{12} **nH**	C_{12} **pF**	L_{21} **nH**	C_{21} **pF**	L_{22} **nH**	C_{22} **pF**	**h mm**
L	5.5 to 9.6	0.2 to 1.5	–	0.5 to 3.8	0.1 to 4.6	0.1	–	4.4	14
S	7.2 to 9.7	–	–	0.3 to 1.7	1 to 8.8	–	–	0.3 to 1.1	4
C	4.4 to 18	–	–	0.1 to 1	0.1 to 4.1	–	–	0.1 to 0.3	7
X	0.1 to 9.2	–	–	0.1 to 0.8	0.1 to 1	–	–	0.1	4
Ku	0.1 to 1	0.1 to 2	–	0.1	0.1 to 0.2	0.1 to 2	–	0.1 to 0.6	4

2.2 High performance of MCES designed metasurfaces.

On the basis of the previous work, this section employs the design of FSS, ESS, and absorbers as representative examples to demonstrate that metasurfaces designed using MCES exhibit competitive performance compared to advanced designs reported in the literature.

2.2.1 FSS Design Based on MCES.

By designing bandstop, bandpass, and dual frequency FSS, the applicability of MCES in FSS design has been fully verified, indicating that MCES can be used to design various FSS.

Supplementary Figure 5 demonstrates the application of the MCES in FSS design by presenting bandpass, bandstop, dual-band bandpass, and dual-band bandstop FSS structures. As illustrated in Supplementary Figure 5a, the bandpass FSS is realized by integrating a parallel circuit comprising an inductor (6 nH) and a capacitor (0.5 pF) on the top layer of the fundamental electromagnetic structure. Supplementary Figure 5e reveals that the designed FSS exhibits a -3 dB bandwidth of 2.8 GHz (1.5-4.3 GHz). For comparison, Supplementary Figure 5i and m display prior work by [1], achieving a -3 dB bandwidth of 0.88 GHz (1.96-2.84 GHz) with a unit cell size as compact as $0.035\lambda_0 \times 0.035\lambda_0$, where λ_0 denotes the resonant wavelength in free space. In contrast, our bandpass FSS design maintains a nearly identical resonance frequency while significantly improving performance—the -3 dB bandwidth is expanded from 0.88 GHz to 2.8 GHz, and the unit cell size is reduced from 0.035 $\lambda_0 \times 0.035\ \lambda_0$ to 0.027 $\lambda_0 \times 0.027\ \lambda_0$, underscoring the superior performance of the MCES-based FSS.

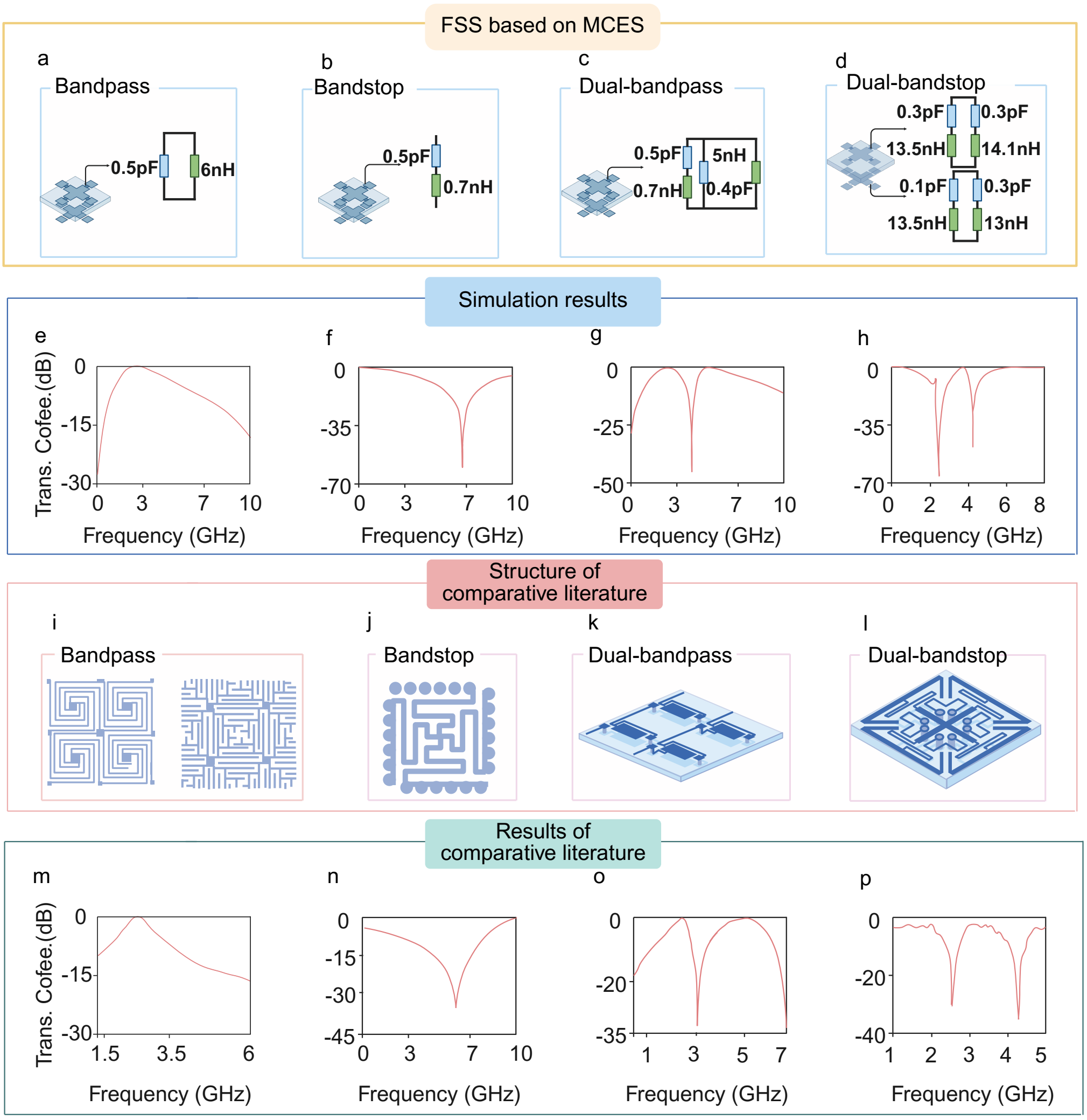

Supplementary Fig. 5: MCES-based FSS design using previously reported FSS structures as design targets. (a-d) MCES-based FSS design. (e-h) Simulation results. (i-l) Conventionally designed FSS [1–4]. (m-p) Performance of conventionally designed FSS. Comparing **e** and **m**, the proposed MCES-based bandpass FSS achieves a similar resonance frequency while significantly enhancing performance: the -3 dB bandwidth is widened by 218% and the unit cell size is reduced to $0.027\lambda_0 \times 0.027\lambda_0$(from $0.035\lambda_0 \times 0.035\lambda_0$). Comparing **f** and **n**, the proposed MCES-based band-stop FSS attains a comparable centre frequency while improving the operating bandwidth by approximately 59% and reducing the unit cell size to $0.063\lambda_0 \times 0.063\lambda_0$(from $0.07\lambda_0 \times 0.07\lambda_0$). Comparing **g** and **o**, the proposed MCES-based dual-band bandpass FSS outperforms this benchmark, expanding both bandwidths and reducing unit cell dimensions to $0.022\lambda_{d1}$ and $0.047\lambda_{d2}$(from $0.053\lambda_{d1}$ and $0.108\lambda_{d2}$). Compared to a reference design (**l** and **p**) with identical centre frequencies but -10 dB bandwidths of 0.28 GHz and 0.35 GHz and a unit cell size of $0.07\lambda_{dL}$ [4], the proposed MCES-based dual-band band-stop FSS expands the total stopband bandwidth from 0.63 GHz to 1.17 GHz and reduces the unit cell size by 66%. Created in BioRender. XU, M. (2026) https://BioRender.com/6xvg14l.

As shown in Supplementary Figure 5b, the band-stop FSS was realized by integrating a series LC circuit (0.7 nH inductance and 0.5 pF capacitance) atop the fundamental electromagnetic structure. Supplementary Figure 5f demonstrates that the MCES-based band-stop FSS achieves a -10 dB rejection bandwidth of 3.5 GHz (5-8.5 GHz). Comparative analysis with prior work (Supplementary Figure 5j, n) reveals that conventional designs [2] exhibit a -10 dB rejection bandwidth of 2.2 GHz (5.4-7.6 GHz) with unit cell dimensions of $0.07\lambda_0 \times 0.07\lambda_0$ (where λ_0 denotes the resonant wavelength in free space). Our band-pass FSS design outperforms these benchmarks by expanding the -10 dB rejection bandwidth from 2.2 GHz to 3.5 GHz while simultaneously reducing the unit cell size from $0.07\lambda_0 \times 0.07\lambda_0$ to $0.063\lambda_0 \times 0.063\lambda_0$ at the identical resonance frequency, confirming the superior performance of the MCES-engineered FSS.

As illustrated in Supplementary Figure 5c, a dual-band bandpass FSS is achieved by integrating a series circuit of an inductor (7.5 nH) and a capacitor (0.2 pF) as well as a parallel circuit of an inductor (5 nH) and a capacitor (0.4 pF) on the top layer of the fundamental electromagnetic structure. Supplementary Figure 5g demonstrates that the designed dual-band bandpass FSS exhibits resonance frequencies at 2.45 GHz (with a -3 dB bandwidth of 1.55 GHz) and 5.0 GHz (with a -3 dB bandwidth of 2 GHz). Supplementary Figure 5k and Supplementary Figure 5o present prior research results, showing resonance frequencies at 2.45 GHz (-3 dB bandwidth of 0.64 GHz) and 5.0 GHz (-3 dB bandwidth of 1.63 GHz), with unit cell sizes as compact as $0.053\lambda_{d1}$ and $0.108\lambda_{d2}$ (where λ_{d1} and λ_{d2} represent the free-space wavelengths at the first and second resonance frequencies, respectively) [3]. Compared to these previous designs, our dual-band bandpass FSS maintains identical resonance frequencies while significantly improving the -3 dB bandwidth from 0.64 GHz to 1.55 GHz and from 1.63 GHz to 2 GHz, respectively. Furthermore, the unit cell size is reduced from $0.053\lambda_{d1}$ and $0.108\lambda_{d2}$ to $0.022\lambda_{d1}$ and $0.047\lambda_{d2}$, demonstrating superior performance of the MCES-based dual-band bandpass FSS design.

Supplementary Table 6: COMPARISON BETWEEN THE PROPOSED FSS AND OTHER REPORTED WORK

Ref	Polarization	Bandwidth	Dimension
[1]	Dual	1.96 GHz-2.84 GHz<br>36.7%	$0.035\lambda_0$
Proposed<br>bandpass FSS	Dual	1.5-4.3GHz<br>96.5%	$0.027\lambda_0$
[2]	Dual	5.4-7.6 GHz<br>34%	$0.07\lambda_0$
Proposed<br>bandstop FSS	Dual	5-8.5 GHz<br>51.9%	$0.063\lambda_0$
[3]	Dual	0.64 GHz @ 2.45 GHz 26.1%<br>1.63 GHz @ 5 GHz 32.6%	$0.053\lambda_{d1}$ / $0.108\lambda_{d2}$
Proposed<br>dual-band bandpass FSS	Dual	1.55GHz @ 2.45GHz 63.2%<br>2 GHz @ 5 GHz 40%	$0.022\lambda_{d1}$ / $0.047\lambda_{d2}$
[4]	Dual	0.28 GHz @ 2.56 GHz 10.9%<br>0.35 GHz @ 4.26 GHz 8.2%	$0.07\lambda_{d1}$
Proposed<br>dual-band bandstop FSS	Dual	0.72 GHz @ 2.56 GHz 28.1%<br>0.45 GHz @ 4.26 GHz 10.6%	$0.024\lambda_{dL}$

The dual-band band-stop FSS design was realized through strategic integration of multiple LC circuits: two series configurations (13.5 nH inductor/0.3 pF capacitor and 14.1 nH inductor/0.3pF capacitor) in the top layer, complemented by two series configurations (13.5 nH inductor/0.1 pF capacitor and 13 nH inductor/0.3 pF capacitor) in the bottom layer, as illustrated in Supplementary Figure 5d. The optimized structure demonstrates dual-band rejection at 2.56 GHz (with a -10 dB bandwidth of 0.72 GHz spanning 2.33-3.05 GHz) and 4.26 GHz (exhibiting a broader -10 dB bandwidth of 0.45 GHz across 4.09-4.54 GHz), as quantified in Supplementary Figure 5h. Comparative analysis with conventional designs (Supplementary Figure 5i,p) reveals identical resonance frequencies at 2.56 GHz (0.28 GHz bandwidth) and 4.26 GHz (0.35 GHz bandwidth) in prior work [4], where the unit cell size was minimized to $0.07\lambda_{dL}$ (λ_{dL} being the free-space wavelength at the lowest resonant frequency). Remarkably, our MCES-based implementation maintains these precise resonance characteristics while achieving significant performance enhancements - expanding the composite -10 dB rejection bandwidth from 0.63 GHz to 1.17 GHz and simultaneously reducing the unit cell dimensions by 66% (from $0.07\lambda_{dL}$ to $0.024\lambda_{dL}$), thereby demonstrating equivalent operational performance with superior miniaturization.

Comparative analysis between our MCES-based FSS designs (exhibiting band-pass, band-stop, dual-band pass, and dual-band stop characteristics) and conventional FSS implementations demonstrates not only the full feasibility of the MCES approach but also its superior performance, as shown in Supplementary Table 6. Crucially, as evidenced in Supplementary Figure 5i-l, traditional FSS designs require complex metallic patterns, vias, and multi-layer architectures to achieve similar functionality — a configuration that significantly increases structural complexity and design iteration time. In stark contrast, the MCES-based FSS maintains simplified topology while achieving comparable

performance metrics, offering substantially improved design efficiency without compromising electromagnetic characteristics. This paradigm shift in FSS implementation suggests remarkable potential for practical applications where both performance and manufacturing simplicity are paramount.

2.2.2 ESS Design Based on MCES.

The applicability of the MCES approach in ESS design has been rigorously demonstrated through successful implementations of band-stop, band-pass, and dual-frequency ESS configurations, confirming its applicability to various ESS designs. However, in light of the prevailing research emphasis on

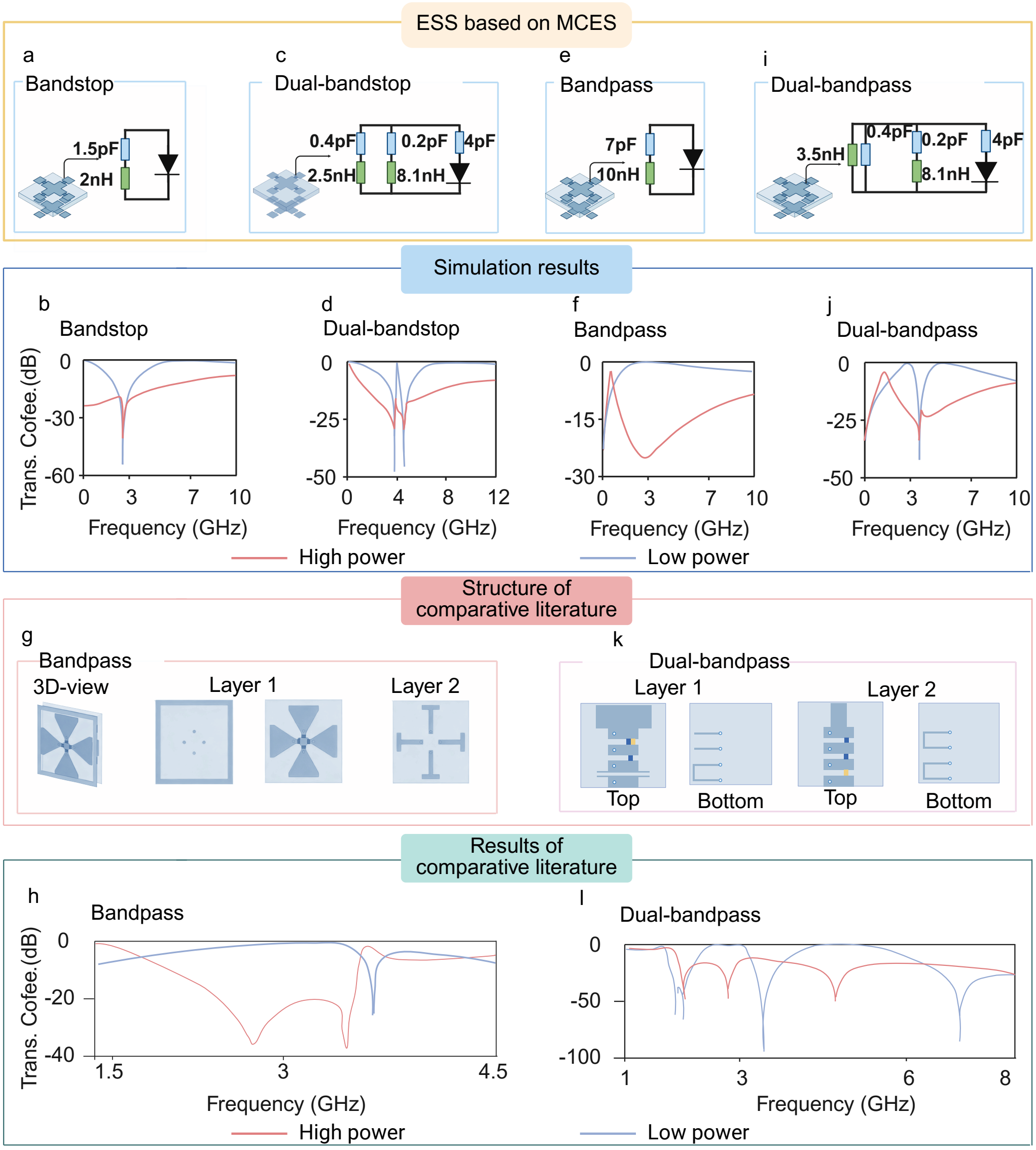

Supplementary Fig. 6: **a**, Band-stop ESS. **b**, Achieves −10 dB stopband of 1.85 GHz (1.75–3.6 GHz) under low-power conditions, expanding to 8.5 GHz (0–8.5 GHz) under high-power excitation. **c**, Dual-band band-stop ESS. **d**, Shows dual rejection bands with −10 dB bandwidths of 0.9 GHz (3.1–4.0 GHz) and 1.1 GHz (4.2–5.3 GHz) under low-power conditions, merging to 9.5 GHz (1–10.5 GHz) protection bandwidth under high power conditions. **e-f**, Band-pass ESS. **f**, Delivers −1 dB bandwidth of 3.5 GHz (1.8–5.3 GHz) in low-power conditions and protective bandwidth of 7.8 GHz (0.9–8.7 GHz) under high power conditions. Reference design (**g-h**): 1.17 GHz −1 dB bandwidth and 1.72 GHz protection bandwidth with $0.26\lambda_0$ unit cell. Proposed design achieves 3× bandwidth enhancement and 8× size reduction ($0.033\lambda_0$ unit cell). **i-j**, Dual-band band-pass ESS. **i**, Exhibits dual insertion-loss bands (< 1 dB) at 2.60–3.10 GHz and 4.7–6.0 GHz under low-power conditions, with shielding effectiveness > 19.4 dB and > 17.2 dB under high power conditions. Reference (**k**): $0.087\lambda_0$ unit cell with comparable dual-band operation. Proposed design reduces unit cell to $0.027\lambda_L$ while maintaining competitive performance. Created in BioRender. XU, M. (2026) https://BioRender.com/gzf875m.

band-pass ESS architectures in the literature, our study specifically focuses on the design and comparative analysis of band-pass ESS relative to existing solutions. For the band-stop configuration, we principally establish the design feasibility using the MCES platform.

Supplementary Figure 6 demonstrates the application of MCES in ESS design by presenting bandpass, bandstop, dual-band bandpass, and dual-band bandstop ESS implementations. As illustrated in Supplementary Figure 6a, the bandstop ESS was realized through a series integration of an inductor (2 nH) and capacitor (1.5 pF) on the top layer of the fundamental electromagnetic structure, connected in parallel with a diode (5 Ω on-state resistance, 0.15 pF off-state capacitance). Supplementary Figure 6b shows that under low-power conditions (diode in off-state), the designed ESS exhibits a -10 dB stopband bandwidth of 1.85 GHz (1.75-3.6 GHz). Under high-power conditions (diode in on-state), the protective bandwidth (S21 < -10 dB) reaches 8.5 GHz (0-8.5 GHz).

As shown in Supplementary Figure 6c, a dual-band bandstop ESS is realized by paralleling three series circuits on the top layer of the fundamental electromagnetic unit: a 2.5 nH inductor in series with a 0.4 pF capacitor, an 8.1 nH inductor in series with a 0.2 pF capacitor, and a diode in series with a 4 pF capacitor. Supplementary Figure 6d reveals that the designed ESS achieves two rejection bands under low-power conditions, with -10 dB bandwidths of 0.9 GHz (3.1-4.0 GHz) and 1.1 GHz (4.2-5.3 GHz). Under high-power conditions, the shielding bandwidth expands to 9.5 GHz (1-10.5 GHz).

Supplementary Table 7: COMPARISON BETWEEN THE PROPOSED ESS AND OTHER REPORTED WORK

Ref	Polarization	Bandwidth[1] (GHz)	Dimension
[5]	Dual	2.44 GHz-3.61 GHz<br>38.7%	$0.33\lambda_0$
[6]	Dual	2.28 GHz-3.81 GHz<br>51%	$0.05\lambda_0$
[7]	Dual	5.4 GHz-14.5 GHz<br>91%	$0.13\lambda_0$
[8]	Dual	6 GHz-10 GHz<br>50%	$0.15\lambda_0$
[9]	Dual	3.2 GHz-3.65 GHz<br>13%	$0.03\lambda_0$
Proposed<br>bandpass ESS	Dual	1.8 GHz-5.3 GHz<br>98.6%	$0.03\lambda_0$
[10]	Single	2.6-3.1 GHz 17.5%<br>4.36-5.46 GHz 22.4%	$0.095\lambda_0$
Proposed<br>dual-band bandspass ESS	Dual	2.6-3.1 GHz 17.5%<br>4.7-6.0 GHz 24.3%	$0.027\lambda_0$

- [1] insertion-loss (IL)<1 dB and shielding effectiveness (SE)>10 dB are used as comparison standards.

As shown in Supplementary Figure 6e and f, the bandpass ESS design was achieved by integrating a series inductor (10 nH) and capacitor (7 pF) in the top layer of the fundamental electromagnetic structure, connected in parallel with a diode. Supplementary Figure 6f demonstrates that the designed ESS exhibits a -1 dB bandwidth of 3.5 GHz (1.8-5.3 GHz) under low-power conditions and a protective bandwidth of 7.8 GHz (0.9-8.7 GHz) in high-power conditions. For comparison, Supplementary Figure 6g-h present prior research results, where the -1 dB bandwidth was 1.17 GHz (2.44-3.61 GHz) in low-power conditions and the protective bandwidth was 1.72 GHz (2.32-4.04 GHz) under high-power conditions, with a unit cell size as small as $0.26\lambda_0 \times 0.26\lambda_0$ (where λ_0 denotes the resonant wavelength in free space) [5]. In contrast, our bandpass ESS design significantly improves performance: the -1 dB bandwidth increases from 1.17 GHz to 3.5 GHz in low-power conditions, while the protective bandwidth expands from 1.72 GHz to 7.8 GHz under high-power conditions. Additionally, the unit cell size is reduced from $0.3\lambda_0 \times 0.3\lambda_0$ to $0.033\lambda_0 \times 0.033\lambda_0$. These results clearly demonstrate the superior performance of the MCES-based bandpass ESS design.

As illustrated in Supplementary Figure 6i,j, the dual-band bandpass ESS is realized by integrating a parallel circuit consisting of three branches: an inductor (3.5 nH) in parallel with a capacitor (0.4 pF), an inductor (8.1 nH) in series with a capacitor (0.2 pF), and a diode in series with a capacitor (4 pF), all implemented on the top layer of the fundamental electromagnetic structure. Supplementary Figure 6j demonstrates that the designed dual-band bandpass ESS exhibits two IL bands below 1 dB

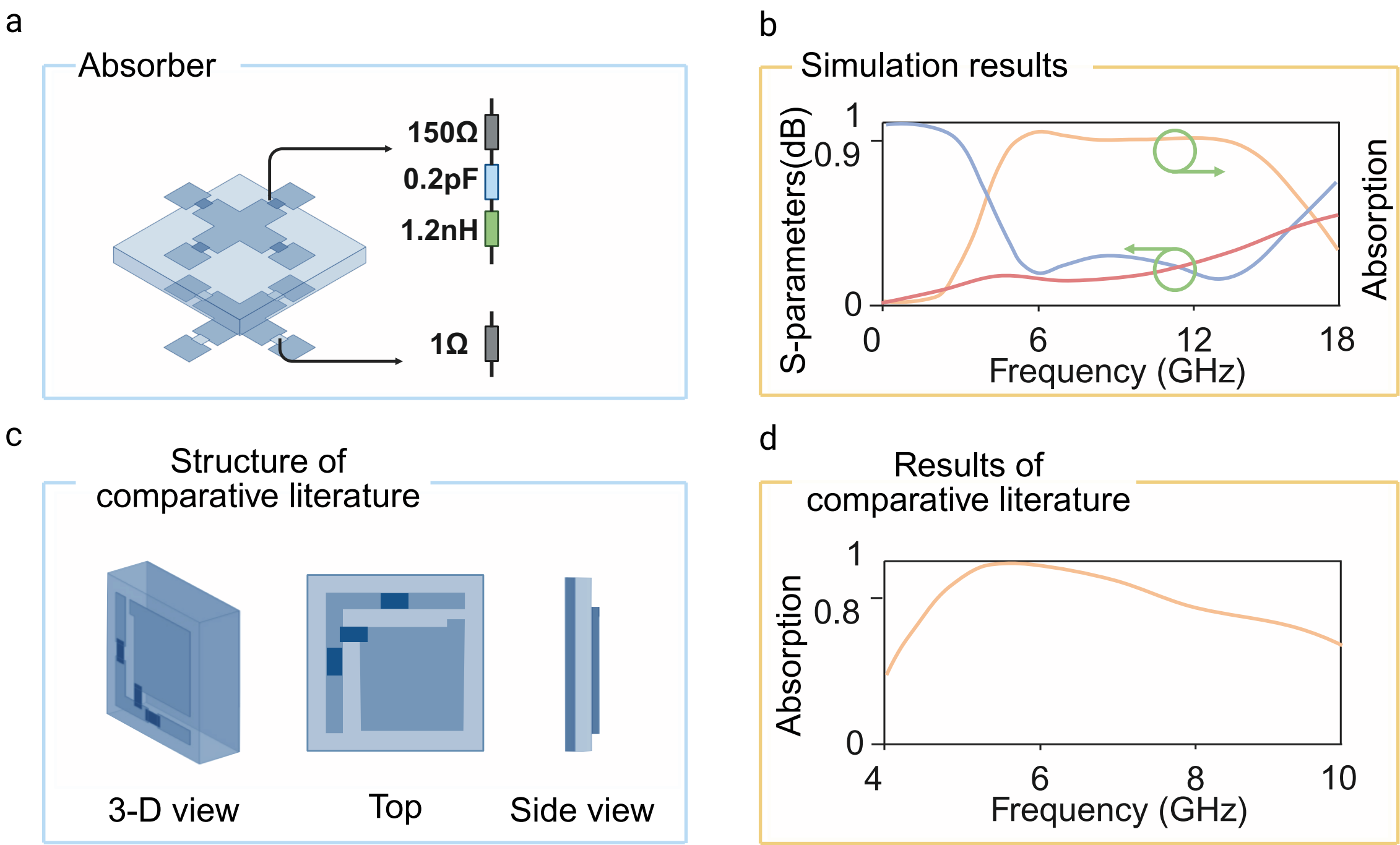

Supplementary Fig. 7: MCES-based absorber design using previously reported absorber structures as design targets. (a-b) MCES-based absorber design. (c-d) Conventionally designed absorber [11]. **a**, Absorber integrating series LC-R network (L = 1.2 nH, C = 0.2 pF, R = 115 Ω) atop the fundamental structure with a bottom resistor (1 Ω). **b**, Full-wave simulation shows $>$ 80% absorption across 4.7–15 GHz and $>$ 90% within 5.2–13.3 GHz. Reference design (**c-d**): $>$ 80% absorption in 4.72–8.49 GHz with $0.22\lambda_0$ unit cell. Our design achieves a 115% bandwidth enhancement (from 3.77 GHz to 8.1 GHz) and 5× miniaturization ($0.092\lambda_0$ unit cell). Created in BioRender. XU, M. (2026) https://BioRender.com/fq45uaj.

in the low-power conditions, covering 2.60-3.10 GHz and 4.7-6.0 GHz, respectively. Under high-power conditions, the SE of these two bands exceeds 19.4 dB and 17.2 dB, respectively. For comparison, prior work (Supplementary Figure 6k, l) achieved dual-band operation with IL $<$ 1 dB over 2.60-3.10 GHz and 4.36-5.46 GHz in the high-power conditions, while the SE surpassed 14.5 dB and 18 dB under high-power conditions, with a unit cell size as compact as $0.087\lambda_0\times\ 0.087\lambda_0$ [10]. In contrast, our ESS design reduces the unit cell dimensions from $0.095\lambda_L\times0.095\lambda_L$ to $0.027\lambda_L\times0.027\lambda_L$ while slightly outperforming the referenced work in terms of low-power bandwidth and high-power shielding performance.

Performance comparison between the bandpass, bandstop, dual-bandpass, and dual-bandstop ESS designed using the MCES approach and those reported in prior studies demonstrates the feasibility and superior performance of MCES-based ESS designs, as shown in Supplementary Table 7. Furthermore, as evidenced in Supplementary Figure 6g,k, conventional ESS designs rely on intricate metallic patterns and multilayer configurations to achieve diverse functionalities, resulting in highly complex structures that demand substantial effort in geometric optimization. In contrast, the MCES-based ESS exhibits a simplified architecture without compromising performance, enabling faster design cycles and offering significant potential for further development.

2.2.3 Absorber Design Based on MCES.

As illustrated in Supplementary Figure 7, we designed an absorber to validate the applicability of MCES in absorber design and compared its performance with prior works. In Supplementary Figure 7a, the absorber is realized by integrating a series configuration of an inductor (1.2 nH), a capacitor (0.2 pF), and a resistor (115 Ω) at the top of the fundamental electromagnetic structure, along with a resistor (1 Ω) at the bottom. Full-wave simulations of the proposed absorber (Supplementary Figure 7b) demonstrate an absorption rate exceeding 80% across 4.7-15 GHz and 90% within 5.2-13.3 GHz. For comparison, prior works (Supplementary Figure 7c-d) achieved $>$ 80% absorption in 4.72-8.49 GHz with a unit cell size as compact as $0.22\lambda_0\times\ 0.22\lambda_0$ [11]. Our design significantly outperforms these benchmarks: the absorption bandwidth is enhanced from 3.77 GHz to 8.1 GHz, while

the unit cell size is reduced from $0.22\lambda_0\times 0.22\lambda_0$ to $0.092\lambda_0\times 0.092\lambda_0$. These results underscore the superior performance of MCES-based absorbers, as shown in Supplementary Table 8.

Supplementary Table 8: COMPARISON BETWEEN THE PROPOSED ABSORBER AND OTHER REPORTED WORK

Ref	Polarization	BW (GHz) $\geq$ 80%	Dimension	BW$_{CE}$ (%)
[11]	Dual	4.72 GHz-8.49 GHz	$0.22\lambda_0$	166.78
[12]	Dual	1 GHz-12.9 GHz	$0.6\lambda_0$	13.21
[13]	Dual	2.68 GHz-12.19 GHz	$0.24\lambda_0$	16.56
[14]	Dual	5.3 GHz-11.2 GHz	$0.12\lambda_0$	41.5
[15]	Dual	2.22 GHz-11.49 GHz	$0.24\lambda_0$	33.47
[16]	Dual	4.6 GHz-20.45 GHz	$0.22\lambda_0$	21.95
[17]	Dual	2.7 GHz-12.7 GHz	$0.23\lambda_0$	57.85
[18]	Dual	2.85 GHz-5.31 GHz	$0.06\lambda_0$	53.15
[19]	Dual	3.1 GHz-14.02 GHz	$0.22\lambda_0$	17.12
[20]	Dual	6.79 GHz-14.96 GHz	$0.21\lambda_0$	16.01
Proposed absorber	Dual	4.7 GHz-15 GHz	$0.092\lambda_0$	753.6

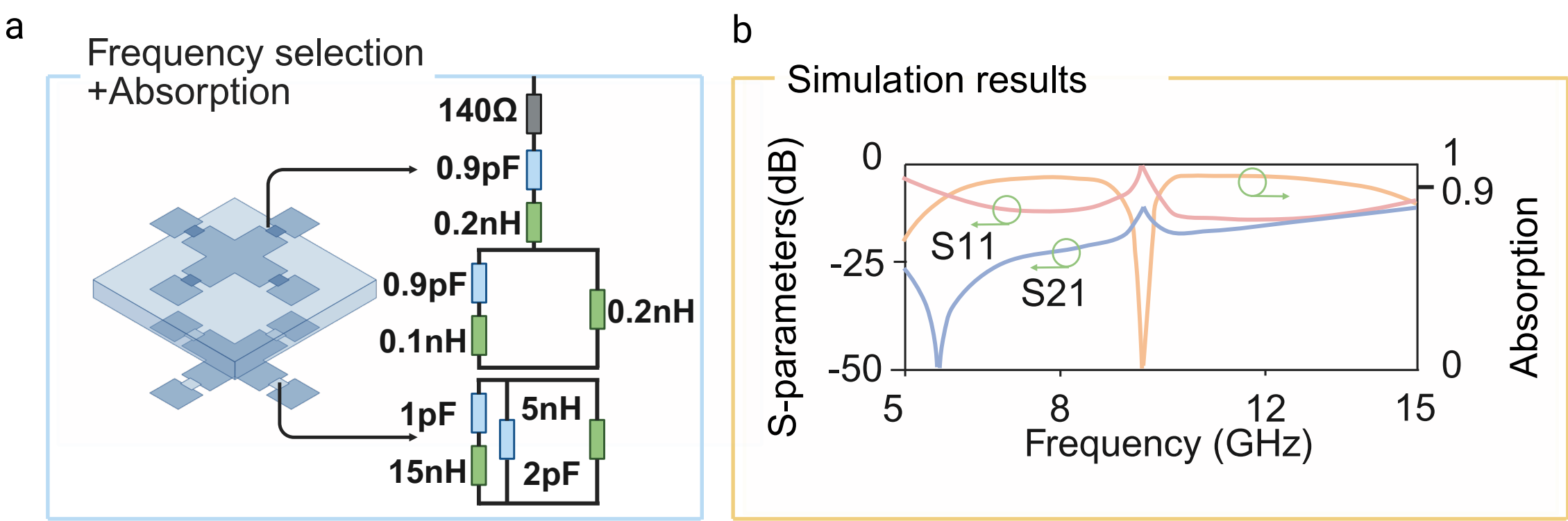

Supplementary Fig. 8: MCES-based absorptive frequency-selective reflector design. (a) Design. (b) Simulation result. **a**, Absorptive frequency-selective reflector integrating a series RLC network ($R = 140\ \Omega$, $L = 0.2$ nH, $C = 0.9$ pF) and series-parallel LC components ($L = 0.1$ nH, 0.2 nH, $C = 0.9$ pF) in the top layer, with a bottom layer comprising a parallel LC network ($L = 15$ nH, $C = 1$ pF) in parallel with an additional LC tank ($L = 5$ nH, $C = 2$ pF). **b**, Simulation reveals dual absorption bands at 6.5–9 GHz and 10.1–13.4 GHz, separated by a 0.24 GHz reflection band (9.56–9.8 GHz), successfully integrating absorber and band-stop FSS functionality. Created in BioRender. XU, M. (2026) https://BioRender.com/z4pan62.

2.2.4 Multi functional metasurface based on MCES.

To validate the feasibility of MCES for multifunctional metasurface design, we conducted the following work. As demonstrated in Supplementary Figure 8, MCES proves applicable not only to single-function metasurface design but also to multifunctional configurations. The proposed absorptive frequency-selective reflector (Supplementary Figure 8a) integrates a series combination of resistor (140 Ω), capacitor(0.9pF) and inductors(0.2 nH) in the top layer, along with series-parallel connections of inductor (0.1 nH and 0.2 nH), capacitors (0.9 pF), while the bottom layer incorporates series-connected inductor (15 nH) and capacitor (1 pF) in parallel with a parallel LC network (5 nH inductor and 2 pF capacitor). Simulation results (Supplementary Figure 8b) reveal dual absorption bands (6.5-9 GHz and 10.1-13.4 GHz) with a measured 3 dB reflection bandwidth of 0.24 GHz (9.56-9.8 GHz) between them, successfully achieving the integration of absorber and band-stop FSS functionalities.

The proposed absorptive energy selective reflector (Supplementary Figure 9a) is realized by integrating in the top layer a series connection of resistor (140 Ω), inductor (0.2 nH) and capacitor (0.9 pF), connected in series with a series-parallel network of capacitor(0.9pF) and inductors(0.1 nH and

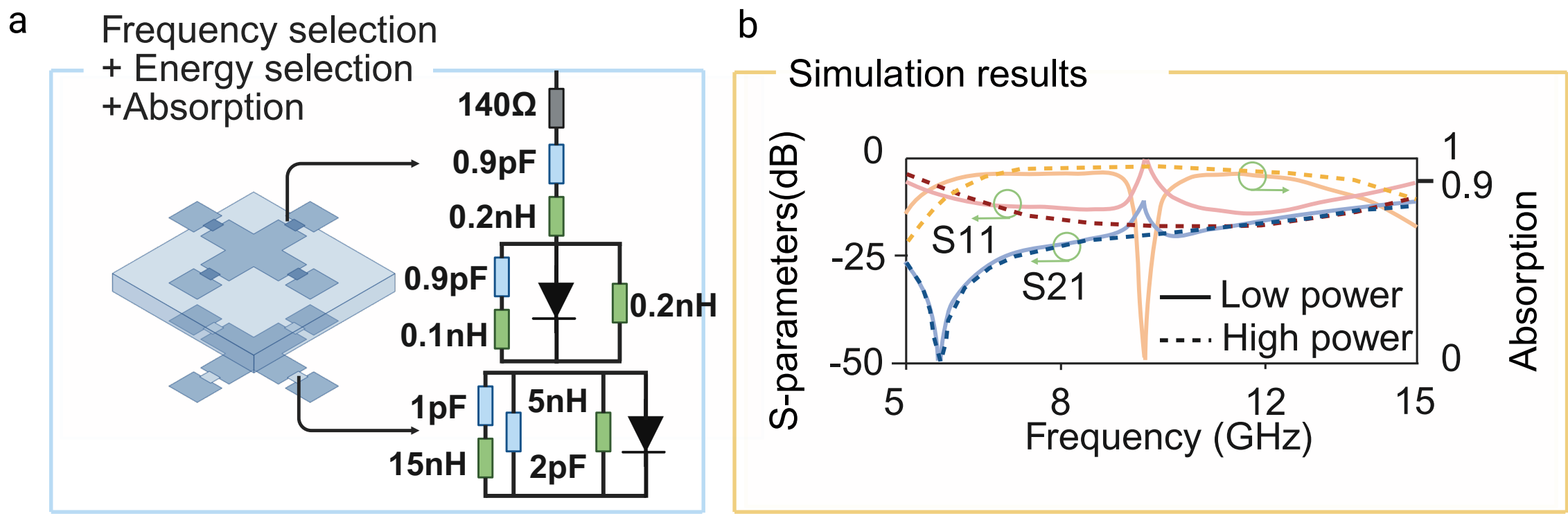

Supplementary Fig. 9: MCES-based absorptive energy-selective reflector design. (a) Design. (b) Simulation result. **a**, Absorptive energy-selective reflector comprising: an top layer with series RLC network ($R = 140\ \Omega$, $L = 0.2$ nH, $C = 0.9$ pF), a series-parallel LC network ($L = 0.1$ nH, 0.2 nH; $C = 0.9$ pF), and a parallel diode; a bottom layer with parallel LC networks ($L = 15$ nH, $C = 1$ pF and $L = 5$ nH, $C = 2$ pF), both diode-terminated. **b**, Under low-power conditions (diodes off), dual absorption bands at 6–9.3 GHz and 10.5–12.6 GHz are separated by a 0.2 GHz reflection band (9.6–9.8 GHz), integrating absorber and band-stop FSS functionalities. Under high-power excitation (diodes on), protection exceeds 10 dB, confirming that the designed reflector simultaneously combines the functionalities of an absorber, band-stop FSS, and ESS, thereby demonstrating the capability of MCES for multifunctional metasurface design. Created in BioRender. XU, M. (2026) https://BioRender.com/uoxb18e.

0.2 nH) , and finally paralleled with a diode, while the bottom layer incorporates a series LC circuit (15 nH inductor and 1 pF capacitor) in parallel with another LC network (5 nH inductor and 2 pF capacitor), both terminated with parallel diodes. Simulation results (Supplementary Figure 9b) demonstrate that under low-power conditions with diodes in off-state, the structure exhibits dual absorption bands (6-9.3 GHz and 10.5-12.6 GHz) with a measured 3 dB reflection bandwidth of 0.2 GHz (9.6-9.8 GHz) between them, successfully integrating absorber and band-stop FSS functionalities. Under high-power conditions with diodes in on-state, the absorption rate exceeds 90% from 6 GHz to 13.6 GHz, confirming that the designed reflector simultaneously combines the functionalities of an absorber, band-stop FSS, and ESS, thereby demonstrating the capability of MCES for multifunctional metasurface design.

Supplementary Note 3. Artificial Intelligence Assisted MCES Metasurface Design

Our previous work has demonstrated MCES's potential in metasurface engineering, though all prior implementations relied on manual design approaches. To advance the application of MCES in metasurfaces, we now explore the integration of AI to automate and optimize MCES-based metasurface design. This AI-driven methodology necessitates three critical phases: comprehensive dataset collection, model training based on the acquired datasets, and rigorous validation of the trained models. We begin by detailing the construction of our training dataset, which serves as the foundation for subsequent AI model development and optimization.

3.1 MCES enables standardized and unified metasurface design through implementation of the GDCM.

Current metasurface research primarily focuses on electromagnetic wave modulation, as exemplified by FSS and absorbers, along with adaptive control demonstrated in ESS. To enable a unified design framework encompassing various functionalities, we propose a novel metasurface architecture that integrates these diverse electromagnetic responses through a systematic design approach. We have established a unified design framework for metasurfaces using a cross shaped metasurface as the skeleton. It is crucial to emphasize that while we employ the cross-shaped metasurface as a demonstrative example in this study, the fundamental architecture of MCES can be constructed using metasurface units of arbitrary geometries — the core concept of MCES encompasses any metasurface unit

integrating multiple circuits to achieve diverse functionalities, without restriction on the specific configuration of the base structural unit. The theoretical support for this approach lies in the classical analysis method of metasurfaces, the equivalent circuit method, which holds that any metasurface structure can be equivalent to an electrical circuit structure, and the performance of different metasurface structures varies due to their different equivalent circuits. Meanwhile, the equivalent circuit method, as a classic metasurface design method, has been widely recognized by researchers and applied in various metasurface designs. Since the birth of metasurfaces, strip and cross shaped structures have been extensively studied by researchers as the most basic metasurface units. Therefore, we have chosen cross shaped structures as the basic framework. In the equivalent circuit method, the metal structure of the cross shaped structure is equivalent to an inductor L_I, and the gap is equivalent to a capacitor C_I. The entire cross shaped structure is equivalent to a series connection of an inductor L_I and a capacitor C_I. By filling different circuits in the gap, it can be equivalent to filling the circuit in parallel with the capacitor C_I.

The width of the cross stripes on the cross shaped metasurface is W_t and a period of P_t for the array. The material of Rogers 5880 with a dielectric constant of 2.2 and a loss tangent of 0.001 was selected as the substrate, and the structural dimensions are shown in Supplementary Figure 10. In the equivalent circuit method, the cross shaped metasurface is equivalent to a two-stage structure with an inductor L_I and a capacitor C_I connected in series [21, 22], as shown in the Supplementary Figure 10, and the dielectric layer is equivalent to a transmission line.

$$L = \frac{Z_0 p_t \sqrt{\varepsilon\mu}}{2\pi}[\ln(\csc\frac{\pi w_t}{2p_t}) + G(p_t, w_t, \lambda)] \tag{1}$$

$$C = 4\frac{p_t\sqrt{\varepsilon\mu}}{2\pi Z_0}[\ln(\csc\frac{\pi g_v}{2p_t}) + G(p_t, w_t, \lambda)]\varepsilon_{\text{eff}} \tag{2}$$

$$G(p, w, \lambda) = \frac{0.5(1-\beta^2)^2[2A(1-\frac{\beta^2}{4}) + 4\beta^2A^2]}{(1-\frac{\beta^2}{4}) + 2A\beta^2(1+\frac{\beta^2}{2}-\frac{\beta^4}{8}) + 2\beta^6A^2} \tag{3}$$

$$\begin{cases} A = \frac{1}{\sqrt{1-(\frac{p}{\lambda})^2}} - 1 \\ \beta = \sin\left(\frac{\pi w}{2p}\right). \end{cases} \tag{4}$$

λ is the wavelength in air at the operating frequency, ε and μ are permittivity and permeability, respectively. This model predicts the correct FSS response in [21] and [22], although it is only valid when $P_t < \lambda$. Fortunately, the unit size of the ESS designed in this article is much smaller than the wavelength, the model can provide an initial value to optimize for the design in this article. The ECM parameters fitted by SA are L=0.67 nH and C = 0.014 pF.

The metacircuit connected in parallel with the structural capacitor C_I includes an RLC network with diodes, that is, a circuit integrated on a cross shaped metasurface. At the same time, in order to adapt the metasurface designed by the framework to more environments, we also took into account the thickness H of the dielectric layer. In the end, various circuit topologies were formed by changing the presence (1), absence (0), and size of diode, H, and each R, L, C, demonstrating different functions and regulating electromagnetic waves differently. We will name different combinations of diode, and $R_{11}/L_{11}/C_{11}/R_{12}/L_{12}/C_{12}/R_{21}/L_{21}/C_{21}/R_{22}/L_{22}/C_{22}/H$ as GDCM codes. For example, when the GDCM code is {0 1 0 0 0 0 0 1 1 1 1 0 0 1 4}, it indicates that the design is FSS, with a structure consisting of a resistor R_{11} and a capacitor C_{12} connected in parallel on the top, and $R_{21}/L_{21}/C_{21}$ connected in series and connected in parallel with capacitor C_{22} on the bottom.

The electromagnetic response of the GDCM is calculated as follows. The MCES consists of passive structures (dielectric and metallic elements) and integrated circuit components (including diodes, R_{ij}, L_{ij}, and C_{ij}). The passive structures remain stationary and interact directly with free-space electromagnetic waves, converting them into guided waves. In contrast, the integrated circuits do not interact directly with spatial waves but instead modulate the electric currents within the circuit. By integrating different circuit configurations, the MCES can effectively manipulate electromagnetic fields in free space. To accurately characterize the relationship between the integrated circuits and the electromagnetic response, we employ microwave network theory for analysis.

The GDCM can be partitioned into three distinct networks. The top and bottom metal layers form two similar series-parallel networks (designated as Network 1 and Network 2), each consisting of an inductor L_I in series with two identical parallel circuits. Each parallel circuit contains a capacitor C_I connected in parallel with a diode and two series circuits: Circuit 1 (R_{i1}, L_{i1}, C_{i1} in series) and Circuit 2 (R_{i2}, L_{i2}, C_{i2} in series), where $i = 1, 2$.

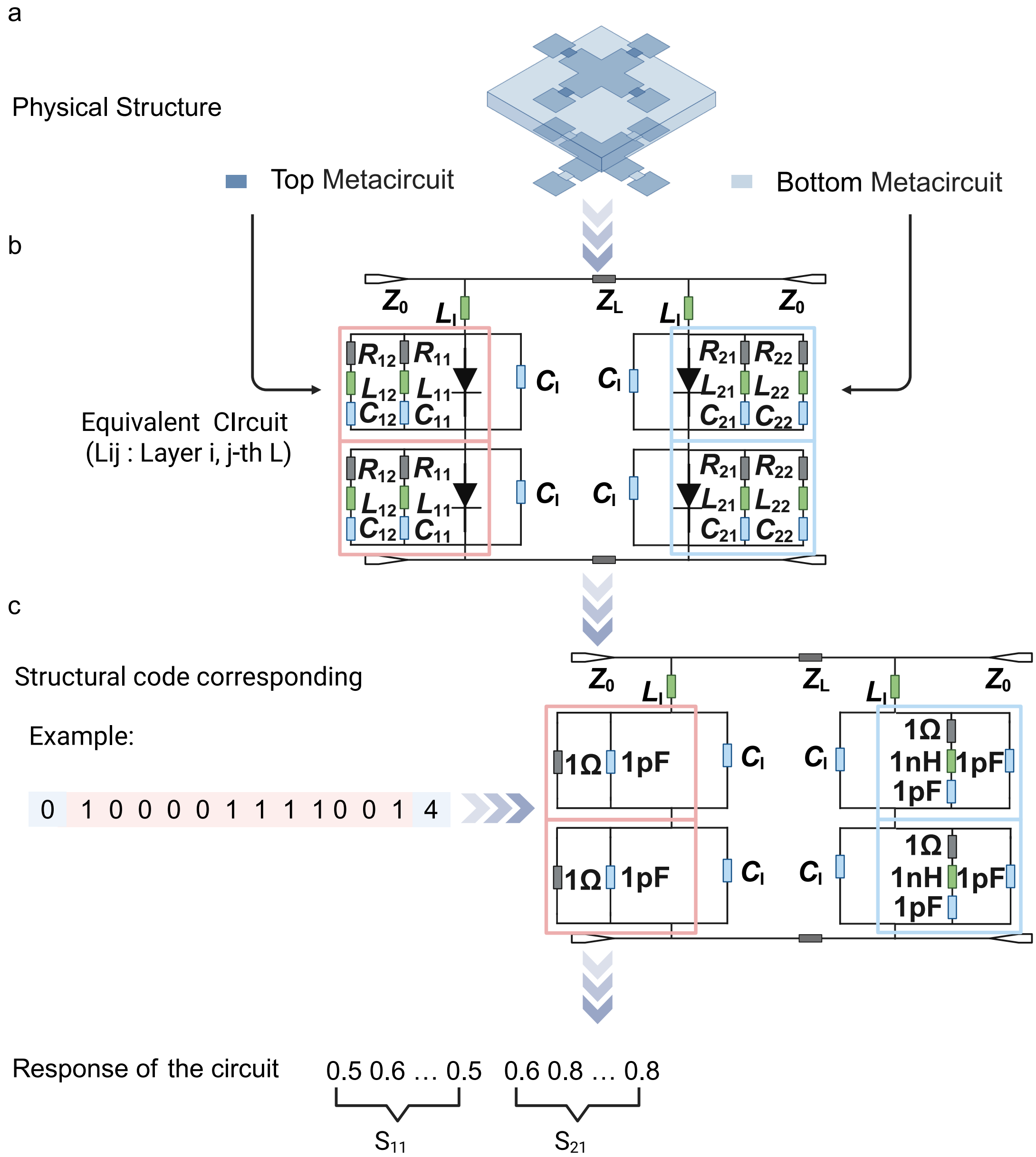

Supplementary Fig. 10: Generalizable design circuit for metasurfaces. **a**, We propose a metasurface design framework using a cross-shaped unit as a reconfigurable backbone. **b**, The metal arms of the cross structure act as an inductor L_I and the gap as a capacitor C_I, forming a base L_I–C_I series circuit. Functional circuits are integrated in parallel with C_I, enabling diverse electromagnetic functionalities. The framework accommodates various circuit topologies—including RLC networks and diodes—by controlling the presence (1) or absence (0) of elements and their values (e.g., R, L, C, diode states, dielectric thickness H). Each configuration is encoded as a GDCM sequence. **c**, Example: GDCM code {0 1 0 0 0 0 1 1 1 1 0 0 1 4} implements a design with top-layer parallel R_{11}–C_{12} network and bottom-layer series R_{21}–L_{21}–C_{21} circuit shunting C_{22}. Created in BioRender. XU, M. (2026) https://BioRender.com/wmfmfi4.

The impedance of inductor L_I is:

$$Z_{L_I} = j\omega L_I \tag{5}$$

The impedance of capacitor C_I is:

$$Z_{C_I} = \frac{1}{j\omega C_I} \tag{6}$$

The impedance of Circuit 1 is:

$$Z_{i1} = \delta_{Ri1} \cdot R_{i1} + j\omega \cdot \delta_{Li1} \cdot L_{i1} + \delta_{Ci1} \cdot \frac{1}{j\omega C_{i1}} \tag{7}$$

The impedance of Circuit 2 is:

$$Z_{i2} = \delta_{Ri2} \cdot R_{i2} + j\omega \cdot \delta_{Li2} \cdot L_{i2} + \delta_{Ci2} \cdot \frac{1}{j\omega C_{i2}} \tag{8}$$

where δ_{Rij}, δ_{Lij}, and δ_{Cij} indicate the presence (1) or absence (0) of components R_{ij}, L_{ij}, and C_{ij}, respectively.

The diode impedance is:

$$Z_{i3} = \gamma \cdot R_{\text{on}} + (1-\gamma) \cdot \frac{1}{j\omega C_{\text{off}}} \tag{9}$$

where γ represents the operational state of the diode (0 for off-state, 1 for on-state).

The capacitor C_I, Circuit 1 (Z_{i1}), Circuit 2 (Z_{i2}), and diode (Z_{i3}) are connected in parallel. The equivalent impedance of this parallel combination is the reciprocal of the total admittance:

$$Z_{\text{parallel}} = \frac{1}{\frac{1}{Z_{C_I}} + \frac{1}{Z_{i1}} + \frac{1}{Z_{i2}} + \delta \cdot \frac{1}{Z_{i3}}} \tag{10}$$

where δ indicates the presence (1) or absence (0) of the diode.

The inductor L_I impedance Z_{L_I} is in series with Z_{parallel}. Therefore, the impedance of Networks 1 and 2 is:

$$Z_i = Z_{L_I} + 2 \cdot Z_{\text{parallel}} = j\omega L_I + 2 \cdot \frac{1}{\frac{1}{Z_{C_I}} + \frac{1}{Z_{i1}} + \frac{1}{Z_{i2}} + \delta \cdot \frac{1}{Z_{i3}}} \tag{11}$$

This simplifies to:

$$Z_i = \frac{j\omega L_I \cdot (j\omega C_I \cdot Z_{i1} \cdot Z_{i2} \cdot Z_{i3} + Z_{i1} \cdot Z_{i3} + Z_{i2} \cdot Z_{i3} + \delta \cdot Z_{i1} \cdot Z_{i2}) + 2 \cdot Z_{i1} \cdot Z_{i2} \cdot Z_{i3}}{j\omega C_I \cdot Z_{i1} \cdot Z_{i2} \cdot Z_{i3} + Z_{i1} \cdot Z_{i3} + Z_{i2} \cdot Z_{i3} + \delta \cdot Z_{i1} \cdot Z_{i2}} \tag{12}$$

The corresponding admittance is:

$$Y_i = \frac{j\omega C_I \cdot Z_{i1} \cdot Z_{i2} \cdot Z_{i3} + Z_{i1} \cdot Z_{i3} + Z_{i2} \cdot Z_{i3} + \delta \cdot Z_{i1} \cdot Z_{i2}}{j\omega L_I \cdot (j\omega C_I \cdot Z_{i1} \cdot Z_{i2} \cdot Z_{i3} + Z_{i1} \cdot Z_{i3} + Z_{i2} \cdot Z_{i3} + \delta \cdot Z_{i1} \cdot Z_{i2}) + 2 \cdot Z_{i1} \cdot Z_{i2} \cdot Z_{i3}}, \quad i = 1, 2 \tag{13}$$

where

$$Z_{ij} = \delta_{Rij} \cdot R_{ij} + j\omega \cdot \delta_{Lij} \cdot L_{ij} + \delta_{Cij} \cdot \frac{1}{j\omega C_{ij}}, \quad i = 1, 2; j = 1, 2 \tag{14}$$

$$Z_{i3} = \gamma \cdot R_{\text{on}} + (1-\gamma) \cdot \frac{1}{j\omega C_{\text{off}}} \tag{15}$$

Here, δ indicates the presence (1) or absence (0) of a diode, and γ represents the operational state of the diode (0 for OFF state, 1 for ON state).

The intermediate dielectric layer (Network 3) is modeled as a transmission line segment with characteristic impedance $Z = Z_0/\sqrt{\varepsilon_r}$, where $Z_0 = 377\ \Omega$ represents the free-space wave impedance.

The transmission matrices of the three individual networks are derived separately. The overall transmission matrix of the complete system is obtained through matrix multiplication of these three matrices. From this composite matrix, the electromagnetic response of the entire network can be computed using the following scattering parameters:

$$S_{11} = \frac{A + B/Z_0 - CZ_0 - D}{A + B/Z_0 + CZ_0 + D} = \frac{\alpha - \beta}{\alpha + \beta} \tag{16}$$

$$S_{21} = \frac{2}{A + B/Z_0 + CZ_0 + D} = \frac{2}{\alpha + \beta} \tag{17}$$

where

$$\alpha = A + B/Z_0, \quad \beta = CZ_0 + D \tag{18}$$

The overall transmission matrix is derived as:

$$\begin{aligned} \begin{bmatrix} A & B \\ C & D \end{bmatrix} &= \begin{bmatrix} 1 & 0 \\ Y_1 & 1 \end{bmatrix} \cdot \begin{bmatrix} \cos(\beta L) & jZ\sin(\beta L) \\ jY\sin(\beta L) & \cos(\beta L) \end{bmatrix} \cdot \begin{bmatrix} 1 & 0 \\ Y_2 & 1 \end{bmatrix} \\ &= \begin{bmatrix} \cos(\beta L) + jZ\sin(\beta L) \cdot Y_2 & jZ\sin(\beta L) \\ Y_1\cos(\beta L) + jY\sin(\beta L) + (jY_1 Z\sin(\beta L) + \cos(\beta L)) \cdot Y_2 & jY_1 Z\sin(\beta L) + \cos(\beta L) \end{bmatrix} \end{aligned} \tag{19}$$

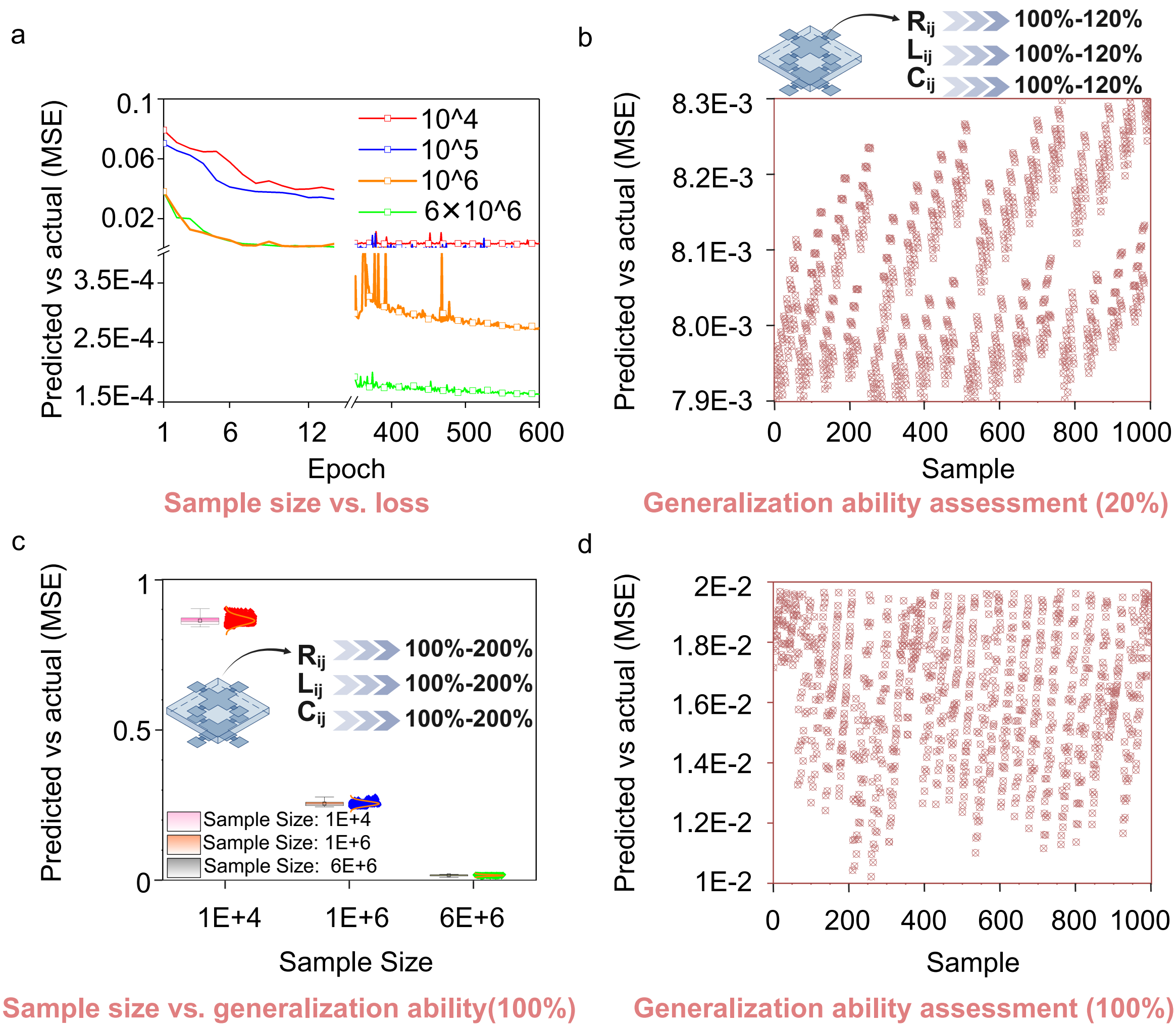

Supplementary Fig. 11: The advantage of a large training sample size. **a**, Loss function evolution for dataset sizes of 10^4, 10^5, 10^6 and 6×10^6, showing faster convergence and improved stability with increased data. **b, d**, Loss distributions for GDCM configurations with 20% and 100% increased R_{ij} L_{ij} C_{ij} values, yielding MSE of (10^{-3}) and (10^{-2}), respectively. **c**, Generalization performance under 100% component variations: models trained on 6×10^6 samples exhibit progressively lower MSE than those trained on 10^4 or 10^5 samples. Created in BioRender. XU, M. (2026) https://BioRender.com/94a9c2k.

3.2 MCES achieves two-order-of-magnitude higher data generation efficiency than conventional approaches.

Our previous work established the correspondence between GDCM codes and electromagnetic responses through the GDCM framework. Since GDCM operates through circuit representations, the corresponding S-parameters can be efficiently obtained via circuit simulations. While the dataset acquisition methodology has been detailed in the main text, we provide here a critical comparison between full-wave and circuit simulation efficiencies to substantiate that the MCES-based approach for AI-driven metasurface design achieves dataset acquisition rates hundreds of times faster than conventional full-wave simulation methods. The acquisition efficiency is quantified by the time required per data point, with shorter durations indicating higher efficiency. Comparative benchmarking performed on the same computer reveals striking differences: for the structure shown in Supplementary Figure 8a, circuit simulation completes in 0.29 seconds while full-wave simulation requires 1 minute 32 seconds, demonstrating a 317-fold (over two orders of magnitude) improvement in data acquisition efficiency through the MCES approach.

3.3 Large training sample size enhances model accuracy and generalization capability.

Building upon the demonstrated 317-fold acceleration in MCES data generation compared to conventional methods, we generated over 6 million data pairs for model training. We systematically investigate the impact of dataset size on model prediction accuracy and generalization capability. Supplementary Figure 11a illustrates the loss function evolution between true value and model predictions at sample sizes of 10^4, 10^5, 10^6, and 6×10^6, revealing faster convergence and more stable final loss values with increasing dataset size. Supplementary Figure 11b,d present the loss distributions when the trained model processes GDCM configurations with 20% and 100% increased $R_{ij}/L_{ij}/C_{ij}$ component values, respectively, showing mean squared errors (MSE) of 10^{-3} and 10^{-2} magnitude. Supplementary Figure 11c demonstrates the superior generalization performance of models trained with larger datasets (6×10^6 samples) compared to smaller ones (10^4 and 10^5 samples) when handling 100% component variations, as evidenced by progressively lower MSE values.

3.4 Leveraging the large-scale datasets generated by MCES, we achieve rapid design of diverse metasurfaces through classical optimization algorithms.

Building upon the forward-prediction neural network of GDCM discussed in the previous section, we employ optimization algorithms to achieve metasurface functionalities within specified frequency ranges. The inverse design workflow has three stages. First, particle swarm optimization searches the circuit parameter space using the trained forward network as a fast evaluator to find configurations that meet a given design target. No ADS simulations are used at this stage. Second, the optimized circuit parameters guide the selection of real components from manufacturer catalogs. Their S-parameter models are imported into CST for full-wave validation. Third, selected designs are fabricated and measured.

This work demonstrates the inverse design capability of our model through three representative examples:ESS, FSS and absorbers. It should be emphasized that our proposed model possesses the versatility to design a wide variety of metasurfaces - these three cases are presented merely as illustrative examples of its capabilities. The EM response characteristics of these functionalities will be defined as the optimization objectives. The optimal GDCM encoding will be determined through multiple iterations. Given that the neural network has already established a robust mapping relationship between GDCM encoding and the corresponding reflection/transmission curves, each iteration of the optimization process will be computationally efficient. Consequently, with minimal resource consumption, the optimal GDCM structure can be effectively identified within the target constraints. The performance of the GDCM unit structure will be further validated through electromagnetic simulations and analysis.

The $fitness$ function is defined as follows, where R and T represent the reflection coefficient and transmission coefficient, respectively. These curves are obtained by inputting GDCM codes into the forward prediction neural network. The overall optimization goal can be categorized based on the specific objectives of electromagnetic wave regulation (e.g., ESS, FSS, absorber, and others), with the aim of minimizing the $fitness$ function to achieve the desired metasurface functionalities:

$$fitness = G(\boldsymbol{R},\boldsymbol{T}) = \begin{cases} k_1|T_{off} - 1dB| - k_2|T_{on} - 10dB| \quad \text{(ESS)} \\ |T^2 + R^2| \quad \text{(absorber)} \\ k_3|T_{TB} - 3dB| - k_4|T_{SB} - 10dB| \quad \text{(FSS)} \\ \text{other.} \end{cases} \tag{20}$$

where R and T represent the reflection coefficient and transmission coefficient. T_{off} and T_{on} denote the ESS transmission coefficients under low-power and high-power conditions. T_{TB} and T_{SB} are the transmission coefficients of the FSS target's transition band and stopband. The scaling factor k_1, k_2, k_3,k_4 are used to adjust the weight.

The particle swarm size is set to 250, and after 100 iterations, the optimized GDCM configurations for ESS, FSS, and absorbers are obtained. After several complete optimization processes, each process only takes about 15 seconds to identify the three optimal samples. The results of ESS, FSS, and absorbers in excellent journals in recent years have been used as the inverse design objectives of the model, where ESS requires IL less than 1 dB and SE greater than 10 dB at 5.4-14.5 GHz (IL less than 1 dB and SE greater than 10 dB within the working bandwidth are industry standards for ESS performance). FSS requires a 3 dB bandwidth greater than 12% and a 20 dB suppression bandwidth

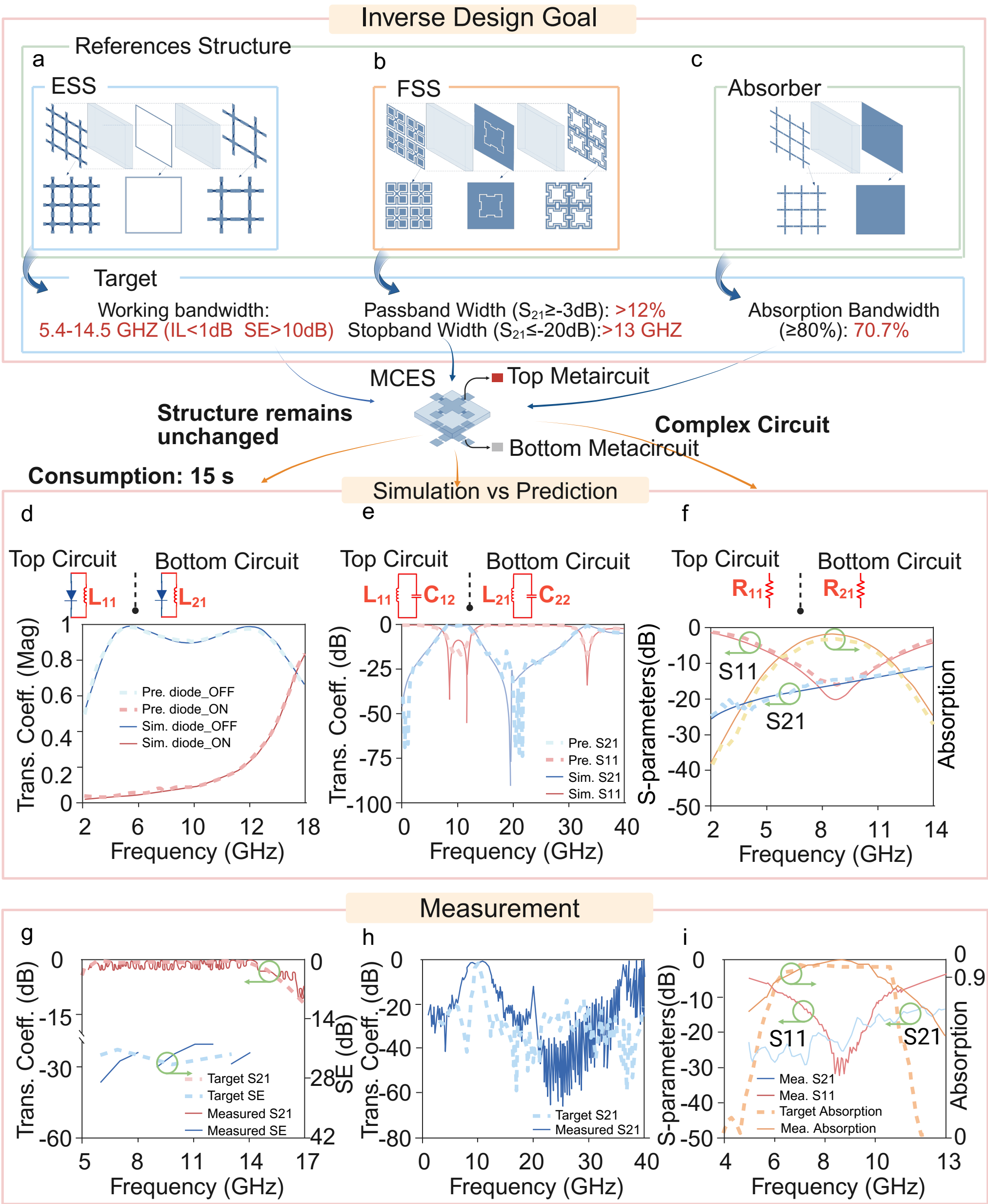

Supplementary Fig. 12: Metasurface inverse design results. **a-c** Inverse design objective. To validate the inverse design capability of the proposed model, we aimed to outperform advanced ESS, FSS, and absorber designs reported in excellent journals. Specifically, the ESS design target was set to IL less than 1 dB and SE greater than 10 dB across 5.4-14.5 GHz (these are industry standards for ESS performance). FSS requires a 3 dB bandwidth greater than 12% and a 20 dB suppression bandwidth exceeding 13 GHz. The bandwidth of the absorber with an absorption rate greater than 80% exceeds 70.7%. (d–f) Comparison of inverse design results and CST simulation values for **d** ESS (GDCM code : {1 0 8 0 0 0 0 0 8 0 0 0 0 4}), **e** FSS (GDCM code : {0 0 1.1 0 0 0 0.2 0 1.1 0 0 0 0.2 5}), and **f** absorbers (GDCM code : {0 160 0 0 0 0 0 1 0 0 0 0 0 5}). (g–i) Experimental results of inverse-designed **g** ESS, **h** FSS, and **i** absorbers. Created in BioRender. XU, M. (2026) https://BioRender.com/pd2xrq0.

exceeding 13 GHz. The bandwidth of the absorber with an absorption rate greater than 80% exceeds 70.7%.The comparison between reverse design and full wave simulation results is presented.

The GDCM code for the designed ESS is {1 0 8 0 0 0 0 0 8 0 0 0 0 4}, as shown in Supplementary Figure 12d. When the diode is in the off-state, S_{21} is greater than -1 dB from 3.7 to 16 GHz; when the diode is in the on-state, S_{21} is less than -10 dB from 0 to 15.1 GHz, indicating that the ESS operates in the 3.7–15.1 GHz band. The performance of this ESS is comparable to that of the latest ESS designs, with detailed comparative data provided in the Supplementary Table 12.

The GDCM code for the designed FSS is {0 0 1.1 0 0 0 0.2 0 1.1 0 0 0 0.2 5}, as shown in Supplementary Figure 12e. It can be observed that the metasurface now functions as an FSS, exhibiting a transmission coefficient greater than -3 dB from 7.5 to 13 GHz (54% fractional bandwidth), less than -10 dB from 0 to 6 GHz and 14 to 29 GHz, and less than -20 dB from 0 to 3 GHz and 16 to 25.2 GHz. This demonstrates a wide transmission passband and strong out-of-band suppression. The predicted and simulated values match well near the passband and maintain consistent trends at higher frequencies. The performance of this FSS is comparable to that of the latest FSS designs, with detailed comparative data provided in the Supplementary Table 13.

The GDCM code for the designed absorber is {0 160 0 0 0 0 0 1 0 0 0 0 0 5}, as shown in Supplementary Figure 12f. It can be observed that the simulated absorption exceeds 80% between 5.3 and 11.8 GHz(76%fractional bandwidth), while the predicted absorption exceeds 90% between 6.5 and 10.6 GHz, indicating a wide absorption band. The predicted results are basically consistent with the full wave simulation results.

3.5 Optimization Algorithm for Inverse Design

This supplementary note provides detailed information about the optimization algorithm used for inverse design in the MCES framework, including implementation parameters, algorithm selection rationale, and comparative performance analysis.

Particle swarm optimization (PSO) is a population-based stochastic optimization algorithm inspired by the social behavior of bird flocks and fish schools. In PSO, a swarm of particles explores the search space, where each particle represents a candidate solution in the continuous parameter space defined by the MCES framework (including diode states, R/L/C values, and substrate thickness). For the metasurface inverse design problems in this work, the PSO algorithm was configured with a swarm size of 250 particles and a maximum of 100 iterations, determined empirically to balance exploration and convergence.

Other optimization algorithms are also compatible with our method. To demonstrate this, we performed a systematic comparative analysis of PSO against three other global optimization algorithms: genetic algorithm (GA), differential evolution (DE), and simulated annealing (SA). The comparison was conducted on 30 independent runs for each algorithm, using identical objective functions and termination criteria. The results are summarized in Supplementary Table 9.

Supplementary Table 9: COMPARATIVE PERFORMANCE OF GLOBAL OPTIMIZATION ALGORITHMS FOR METASURFACE INVERSE DESIGN (30 INDEPENDENT RUNS)

Algorithm	Best Objective	Mean Objective	Standard Deviation	Average Time (seconds)
PSO (proposed)	-2.00266	-2.00182	0.000439	1.55
Genetic Algorithm	-2.00230	-2.00187	0.000335	2.75
Differential Evolution	-2.00166	-2.00102	0.000321	0.47
Simulated Annealing	-2.00252	-2.00218	0.000159	9.22

All four algorithms achieved 100% success rates, consistently meeting the design specifications. Simulated annealing gave the best mean objective value (-2.00218) with the smallest standard deviation (0.000159). PSO ranked second (-2.00182), closely followed by GA (-2.00187). DE achieved the lowest mean objective (-2.00102) among the four algorithms.

In terms of speed, DE was the fastest at 0.47 seconds per run, followed by PSO at 1.55 seconds, GA at 2.75 seconds, and SA at 9.22 seconds. GA achieves similar solution quality but requires nearly twice the computation time of PSO. DE converges fastest but yields the poorest solution quality among all algorithms tested. PSO offers the best balance between solution quality and computational efficiency, delivering results comparable to SA (-2.00182 vs -2.00218) while being approximately six times faster (1.55 seconds vs 9.22 seconds).

Most importantly, all four algorithms consistently found valid designs. This confirms that the MCES framework creates a well-conditioned design space where even simple optimization methods

work reliably. The framework's strength lies in its data generation capability and unified design paradigm, not in algorithmic complexity.

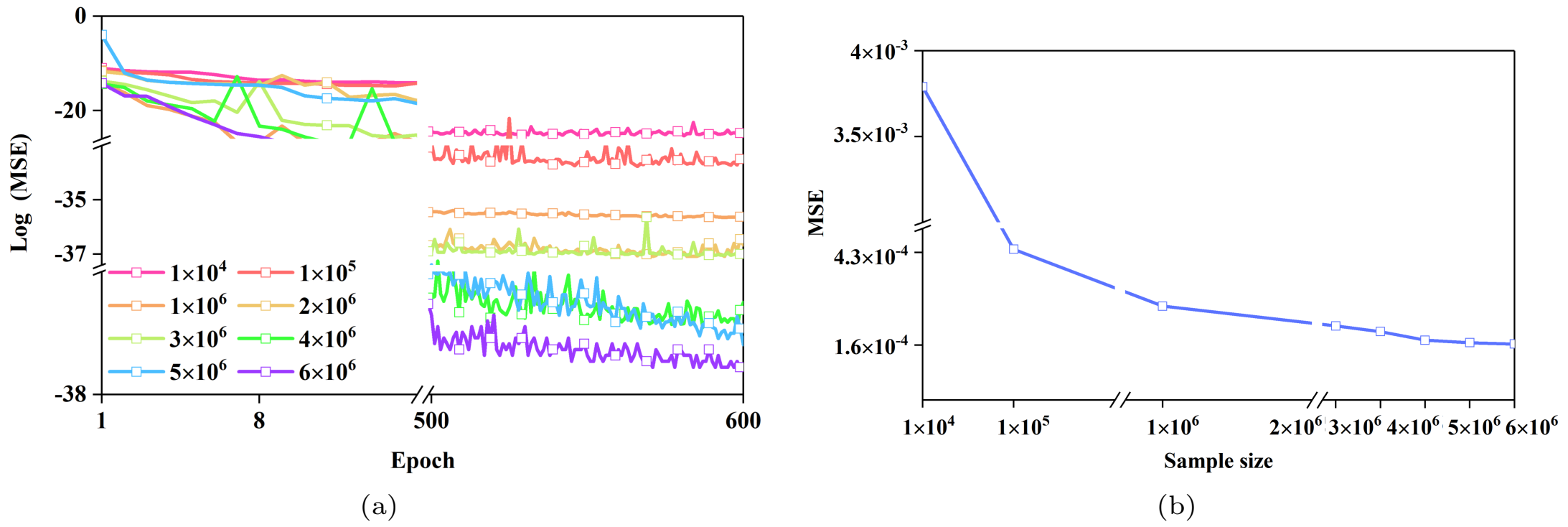

Supplementary Fig. 13: Validation MSE versus dataset size. (a) Validation MSE on a logarithmic scale. (b) Validation MSE in linear scale.

3.6 Dataset Size Justification Analysis

To quantitatively assess the impact of dataset size on model performance, we trained models using sample sizes of 1×10^4, 1×10^5, 1×10^6, 2×10^6, 3×10^6, 4×10^6, 5×10^6, and 6×10^6, as shown in Supplementary Figure 13 and Supplementary Table 10.

As the dataset size increases from 1×10^4 to 1×10^6, the validation MSE decreases by approximately 92.8%. The rate of improvement slows after 3×10^6 samples, with an additional reduction of only 18.2% when increasing from 3×10^6 to 6×10^6 samples. As illustrated in Supplementary Figure S13(b), the trend of the model MSE flattens with increasing dataset size, which clearly indicates diminishing marginal returns.

To evaluate generalization capability, we selected an out-of-sample circuit configuration not included in any training subset (GDCM code: 0 450 21 0.6 450 21 0.6 45 21 6 4). The MSE between the model predictions and full-wave simulation results is reported in Supplementary Table 11 and Supplementary Figure 14.

The model trained on 6×10^6 samples achieves an out-of-sample MSE of 6.06×10^{-4}, a reduction of approximately 91,073 times compared to the model trained on 1×10^4 samples. Notably, the out-of-sample MSE reaches the 10^{-3} level at 5×10^6 samples, indicating practically useful accuracy.

Based on the above analysis, we summarize the key observations regarding dataset size effects. Model performance continues to improve up to 6×10^6 samples, with the rate of improvement diminishing after 3×10^6 samples. The validation MSE stabilizes around 1.6×10^{-4} beyond 5×10^6 samples. Some redundancy exists in the dataset, but it is not excessive. The validation MSE continues to decrease from 1×10^6 to 6×10^6 samples (40.3% reduction), and the out-of-sample error drops by a factor of 180 over the same range, confirming that additional samples provide meaningful new information. Diminishing returns are clearly observed: the MSE reduction is 92.8% from 1×10^4 to 1×10^6 samples, but only 18.2% from 3×10^6 to 6×10^6 samples.

Regarding the minimum viable dataset size, a dataset of 1×10^6 samples yields an out-of-sample MSE of 0.110, which is insufficient for practical use. Increasing to 5×10^6 samples brings the error to the 10^{-3} level (0.00109), achieving practically useful accuracy. The full 6×10^6 dataset further improves the error to 6.06×10^{-4}. Thus, a minimum of 5×10^6 samples is recommended for reliable design tasks.

Supplementary Table 10: VALIDATION MSE FOR MODELS TRAINED WITH DIFFERENT DATASET SIZES (600 EPOCHS)

Dataset Size	Validation MSE	Dataset Size	Validation MSE
1×10^4	3.79×10^{-3}	3×10^6	1.99×10^{-4}
1×10^5	4.39×10^{-4}	4×10^6	1.74×10^{-4}
1×10^6	2.73×10^{-4}	5×10^6	1.66×10^{-4}
2×10^6	2.15×10^{-4}	6×10^6	1.63×10^{-4}

Supplementary Table 11: OUT-OF-SAMPLE MSE FOR MODELS TRAINED WITH DIFFERENT DATASET SIZES

Dataset Size	Out-of-sample MSE	Dataset Size	Out-of-sample MSE
1×10^4	55.19139	3×10^6	0.04398
1×10^5	26.44115	4×10^6	0.03751
1×10^6	0.11023	5×10^6	0.00109
2×10^6	0.045	6×10^6	6.06007×10^{-4}

Finally, the generalization improvement has clear practical engineering significance. The 180-fold reduction in out-of-sample error from 1×10^6 to 6×10^6 samples directly translates to more accurate inverse design for unseen configurations. Achieving prediction errors on the order of 10^{-4} with the full dataset ensures that the trained model is suitable for practical metasurface design applications.

Supplementary Note 4. Experimental verification of MCES

To validate the functionality of metasurfaces designed using the MCES framework, we fabricated and tested FSS, ESS, and absorber prototypes. The experimental validation in this study employs two different test methods, each following standard measurement methods in their respective fields [9, 23–29].

4.1 Experimental verification of ESS based on MCES.

With the continuous expansion of ESS functionality and performance, the design of ESS has become increasingly complex. The better performance and versatility are often caused by its complex structure. Therefore, the design of ESS has fallen into a complex design. The MCES proposed in this article provides new ideas and methods for the design of ESS, which extends performance and functionality by adding circuits to one or more simple structures. The analysis of simple structures is extremely simple, and circuit design can be achieved quickly through various design software. The combination of simple structures and complex circuits can greatly simplify design difficulties, save time in optimizing structures, improve design speed, and expand performance and functionality.

Having detailed the inverse-designed ESS structure and its simulated performance under periodic boundary conditions in preceding sections, we now detail the waveguide simulation results, experimental methodology, and measured performance of the ESS prototype. The IL and SE of the infinite ESS in the free space are simulated with periodic boundary, while the ESS is finite in reality. Generally, as long as the size of ESS exceeds ten wavelengths, the edge effect can be ignored in the test to obtain accurate results. Another ingenious measurement method to evaluate the performance of the periodic structure is using a parallel plate waveguide with a TEM mode plane wave [30–32], which requires less area of the sample and does not need an anechoic chamber. However, the SE of the ESS cannot be tested due to the low power electric field withstand of the parallel plate waveguide. In order to reduce the difficulty of testing, constructing high-intensity electric field in the waveguide is also a common test method [5]. In this article, the waveguide test method is also used to verify the performance of the proposed ESS.

Since the main mode TE10 frequency of a single standard rectangular waveguide cannot cover a frequency band as wide as 4–14 GHz, standard rectangular waveguides of WR137, WR90, and WR62 are selected as the test environment to facilitate comparison with the literature. The inner wall sizes of the three waveguides are 34.85 × 15.80 mm, 22.86 × 10.16 mm, and 15.80 × 7.90 mm, respectively. And the main mode TE10 frequency can cover 5.3–8 GHz, 8–12.5 GHz, and 12.5–18 GHz, which can satisfy the test requirements of the designed ESS. The boundary conditions of all sides in the waveguide are perfect electrical conductor (PEC) which is different from the periodic boundary in the mentioned above simulation. Therefore, before the measurement, full-wave simulation is carried out in the waveguide to verify the transmission performance of the ESS in the waveguide.

Furthermore, the designed ESS unit is optimized according to the inner wall dimensions of the C-band, X-band, and Ku-band waveguide. The transmission coefficients of the ESS in different waveguides are simulated with diode off and diode on by CST, and the results are shown in Supplementary Figure 15d. At the same time, the simulation results of the ESS in free space are also shown in the figure as a comparison. When the diode is turned off, the changing trend of the ESS transmission

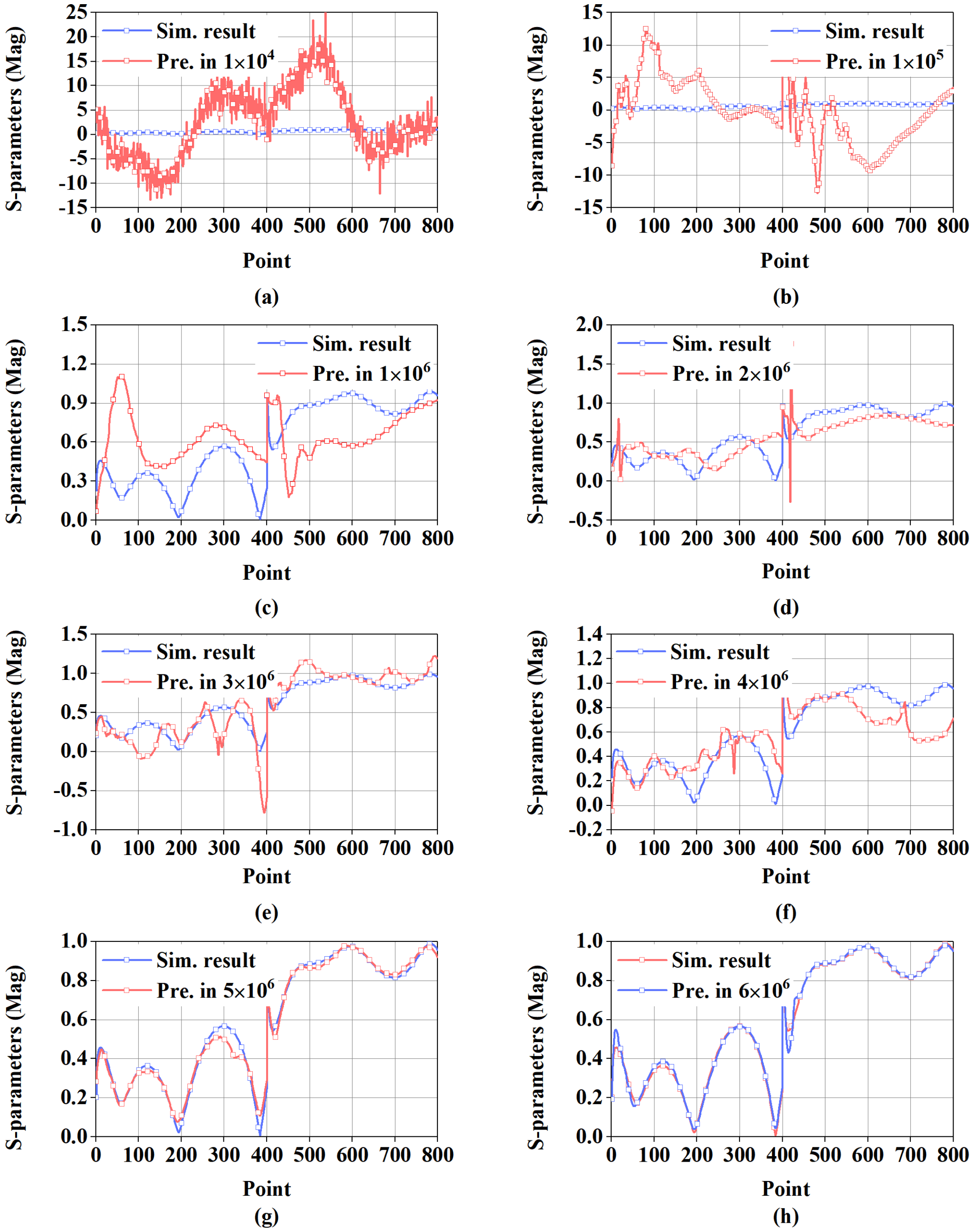

Supplementary Fig. 14: Predicted versus simulated responses for an out-of-sample configuration using models trained with different dataset sizes.

coefficient in the waveguide is consistent with the variation trend in free space, which is greater than -1 dB in 4.85-14.5 GHz, although it decreases rapidly in the low-frequency band because it is close to the cut-off frequency of the C-band waveguide. When the diode is turned on, the SE of ESS in the waveguide is better than that in free space. In conclusion, the simulation results show that the method of waveguide test can be used to preliminarily prove the correctness of the design concept of the ultra-broadband ESS proposed in this article.

To verify the performance of the designed ESS, three ESS prototypes are fabricated by printed circuit board technology, as shown inside of Supplementary Figure 15e. To simplify the connection with the waveguide, the outside size of the prototype is consistent with the waveguide flange, and the ESS size agrees with the inner wall dimension of the waveguide. There are many vias around the prototype for connecting waveguide flanges on both sides.

The IL of the prototype in low-power conditions is measured with a vector network analyzer (VNA) and waveguide. In the test, the output port and input port of the VNA are respectively connected to

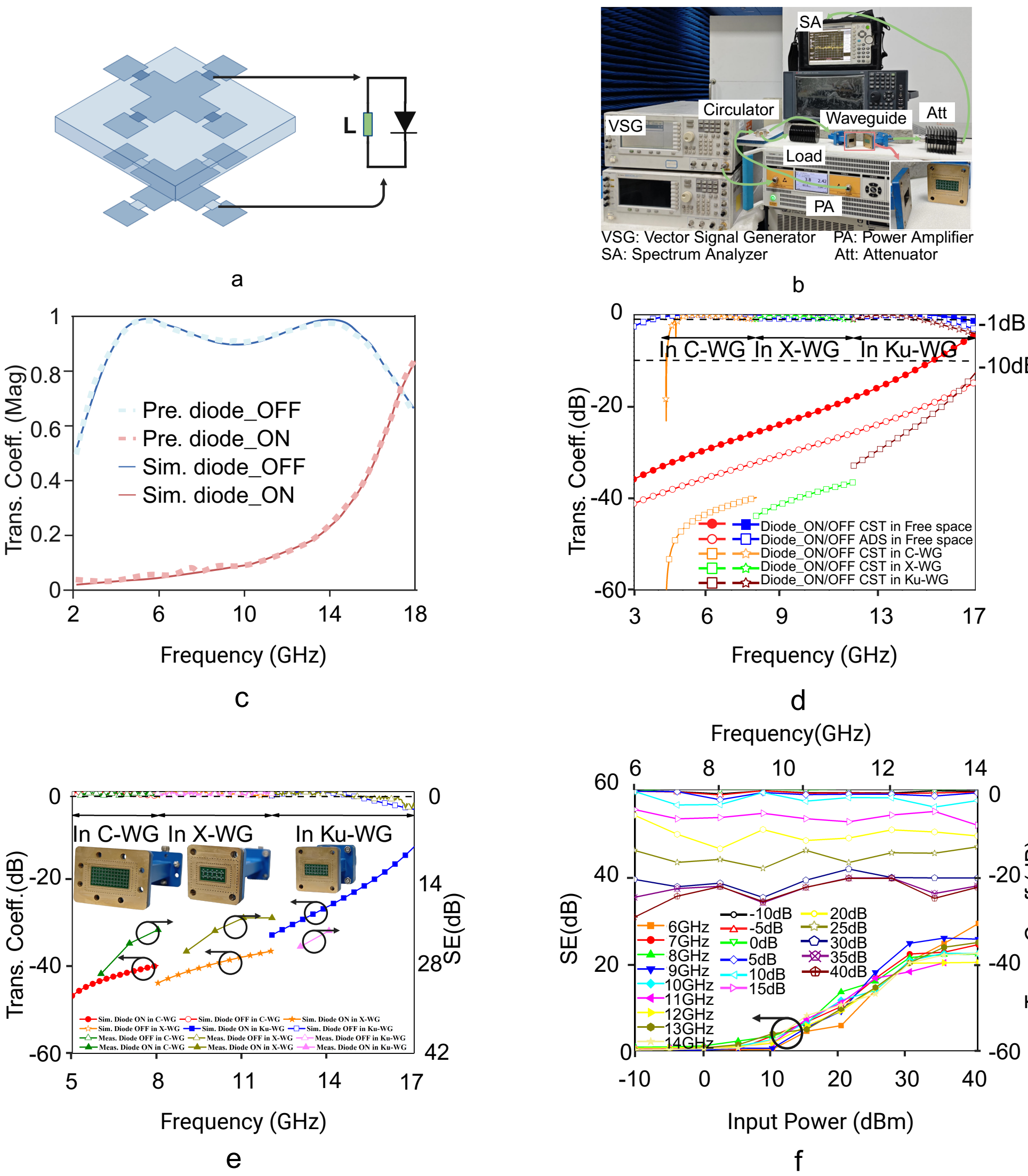

Supplementary Fig. 15: Measurement of ESS. (a) ECM of the ESS proposed based on UDCM. (b)Experimental setup for HIRF. (c)Comparison between predicted and simulated performance of the inversely designed ESS(periodic boundary). (d)Transmission coefficients of ESS in ADS and CST. (e) Transmission coefficient measurement results of the ESS under LIRF and HIRF. (f)SE and transmission coefficient measurement results of the ESS under different injection power. Created in BioRender. XU, M. (2026) https://BioRender.com/dv6dbgo.

the two waveguide coaxial connectors of the waveguide, and the output power is set to less than -25 dBm. According to the electric field and magnetic field distribution of TE10 mode in the waveguide, the peak value of electric field intensity at the center of the waveguide can be calculated as 5.2 V/m (at 10 GHz) which is small enough to keep the diode off. Three prototypes are tested in the operating frequency bands of the three waveguides, and the results are shown in Supplementary Figure 15f. In the C-band waveguide, the 1 dB IL band of ESS covers 5.2–8 GHz. As it is close to the cut-off frequency of the C-band waveguide, it shows rapid attenuation in the low-frequency band, which is consistent with the simulation results in the waveguide. In the X-band and Ku-band waveguides, the 1 dB IL band of ESS covers 8–12 GHz and 12–14.5 GHz, respectively. Thus, the ESS prototype has low IL performance in 5.2–14.5 GHz. The test results are in good agreement with the simulation results.

To demonstrate the performance of SE and nonlinear response of the ultra-broadband ESS in high-power conditions, a rectangular waveguide with a high power amplifier (PA) measurement scenario is established as shown in Supplementary Figure 15b. High-power signal is generated by a vector signal generator (VSG) and amplified by PA, the power level of which is controlled by the VSG. The PA output port is connected in series with a circulator to prevent the reflection of the high-power microwave from damaging the PA, and a load is installed on the third port of the circulator to absorb reflected power. Then, the high-power signal enters the waveguide. The power level passing

Supplementary Table 12: COMPARISON BETWEEN THE PROPOSED ESS AND OTHER REPORTED WORK

Ref	Polarization	Simulated Bandwidth[1] (GHz)	Measured[2] IL/SE
[6]	Single	2.28 GHz-3.81 GHz<br>50%	0.6 dB/20 dB
[7]	Dual	5.40-14.45 GHz<br>91.1%	0.01 dB/24 dB
[33]	Single	<1.1 GHz<br>Low bandpass	4 dB/18 dB
[5]	Single	2.60-3.52 GHz<br>30%	0.6 dB/31.8 dB
Proposed	Dual	5.2-14.5 GHz<br>94.4%	0.01 dB/27.2 dB

- [1] IL<1 dB and SE>10 dB are used as comparison standards.
- [2] Maximum SE and minimum IL were selected for the comparison.

through the ESS prototype inside of the waveguide is monitored by a spectrum analyzer. Before the measurement, the loss of the test system itself needs to be calibrated to accurately calculate the signal power injected into the waveguide.

By adjusting the output power of the VSG from small to large, the electric field strength of different power can be obtained in C/X/Ku waveguide. Define the monitored waveguide input power and output power as P_{in} and P_{out} respectively, then the SE of the ESS can be calculated by $\text{SE(dB)} = P_{\text{in}}(\text{dBm}) - P_{\text{out}}(\text{dBm})$. The maximum electric field in the center of the waveguide can be obtained by the following equation according to the input power:

$$E_{\text{TE10}} = \sqrt{\frac{4\eta P_{\text{TE10}}}{ab\sqrt{1-(\lambda/2a)^2}}}. \tag{21}$$

With the increase of the incident power level from −10 dBm to the maximum input power, the nonlinear performance of the ESS in C/X/Ku band waveguide is measured with different frequencies, and the curves of SE versus input power are shown in Supplementary Figure 15f. The measurement results show that all ESS prototypes are in a low IL state when the injection power is small, and then the SE gradually improves with the increase of injection power. In the C-band waveguide, the SE is tested at 6, 7, and 8 GHz. The maximum SE 27.25 dB, 24.4dB , and 22.2dB are obtained at 6, 7, and 8 GHz, respectively. For the ESS in X-band waveguide, the threshold of the diode starting to turn on is 25 dBm. At this time, the maximum electric field in the center of the waveguide is 1.6 kV/m (at 10 GHz), that is to say, the threshold of the ESS in free space is around 1.6 kV/m. When the input power reaches its maximum value, the maximum SE is 25.6 dB at 9 GHz, 22.4 dB at 10 GHz, 20.3 dB at 11 GHz, and 20.3 dB at 12 GHz, respectively. The nonlinear performance of ESS in Ku band waveguide was measured at 13 and 14 GHz. When the input power is 40 dBm (with maximum electric fields of 13.3 and 12.8 kV/m), the maximum SE at 13 and 14 GHz is 24.9 dB and 22.3 dB, respectively.

The transmission coefficient of ESS is defined by $S_{21}(\text{dB}) = P_{\text{out}}(\text{dBm}) - P_{\text{in}}(\text{dBm})$, and the relationship between SE and transmission coefficient can be expressed by $SE(\text{dB}) = -|S_{21}|(\text{dB})$. In order to further illustrate the relationship between the transmission coefficient of the proposed ESS and the input power, the transmission coefficient under different power is shown in Supplementary Figure 15f. Obviously, as the input power increases, the transmission coefficient of the ESS decreases. When the input power exceeds 25 dBm, the transmission coefficient is less than −10 dB, that is, the SE is greater than 10 dB.

Based on the above simulation and test results, it is preliminarily proved that the design method of using GDCM to guide the design of ultra-broadband ESS is feasible, and the designed ESS has the performance of low IL and high SE in the band of 5.2–14.5 GHz. The main performance of the proposed ESS in this article is compared with that is designed in other research as shown in Supplementary Table 12. It is observed that the ESS designed in this article works in an ultra-broadband, which overcomes the problems of large IL and low SE in the wide and high-frequency band. Moreover, the proposed designs are dual-polarized which can be applied to dual polarization RF front-end.

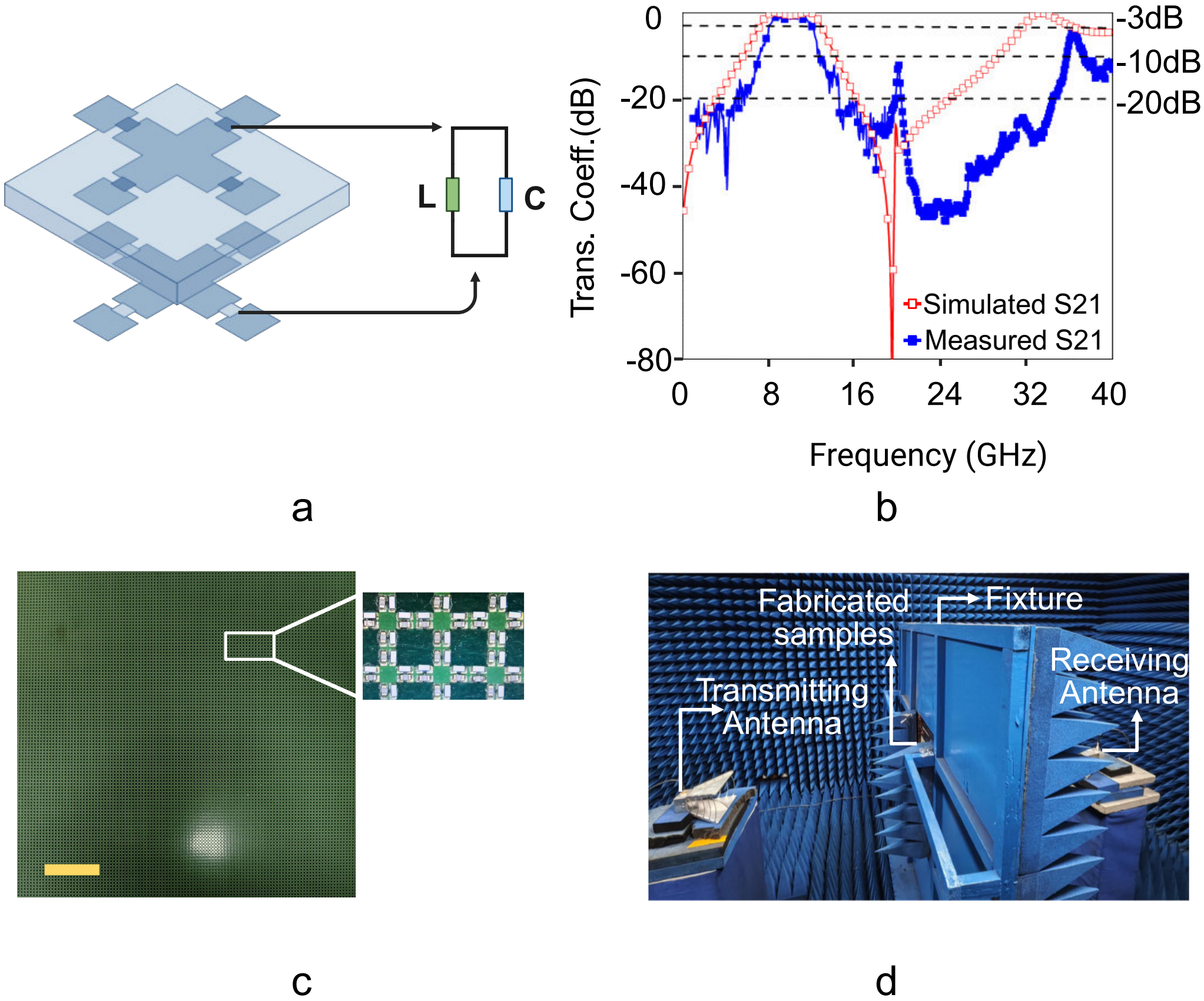

Supplementary Fig. 16: Measurement of FSS. (a) The proposed FSS based on MCES (GDCM code: {0 0 1.1 0 0 0 0 0.2 0 1.1 0 0 0 0 0.2 5}). (b)Comparisons between the simulated and the measured results. (c)Photograph of the FSS prototype. The scale bar, 5 cm. (d)Photograph of the experimental setup. Created in BioRender. XU, M. (2026) https://BioRender.com/0d60krt.

Supplementary Table 13: COMPARISON BETWEEN THE PROPOSED FSS AND OTHER REPORTED WORK

**Ref**	**Dimension**	**Thickness**	**Passband width (S_{21} $\geq$ -3 dB)**	**Insertion loss**	**Stopband (S_{21} $\leq$-20 dB)**
[30]	0.13λ	0.05λ	9%	1 dB	N/A
[34]	0.33λ	0.16λ	12%	about 0.2 dB	13 GHz (11 to 24 GHz)
[35]	0.21λ	0.05λ	15.7%	N/A	6 GHz (24 to 30 GHz)
[36]	0.05λ	0.19λ	40%	about 0.2 dB	8.7 GHz (5 to 13.7 GHz)
[37]	0.11λ	0.017λ	3.7%	0.5 dB	11 GHz (2 to 3 GHz 3.5 to 13.5 GHz)
Proposed	0.09λ	0.13λ	41%	0.1 dB	20 GHz (0 to 5 GHz 14.4 to 19.4 GHz 20.6 to 30.6 GHz)

4.2 Experimental verification of FSS based on MCES.

The FSS of the model design is shown in Supplementary Figure 16a, the structural code of GDCM is {0 0 1.1 0 0 0 0 0.2 0 1.1 0 0 0 0 0.2 5}, which means that the response layer is a parallel connection of inductor L ($L11 = L21 = 1.1$nH) and capacitor C ($C12 = C22 = 0.2$pF), achieving the function of FSS. At this point, the equivalent circuit diagram and simulation results of the corresponding GDCM are shown in Supplementary Figure 12e.

For further verification, an FSS prototype has been fabricated and measured. As shown in Supplementary Figure 16c, the overall size of the manufactured prototype is 320 mm $\times$ 320 mm. The

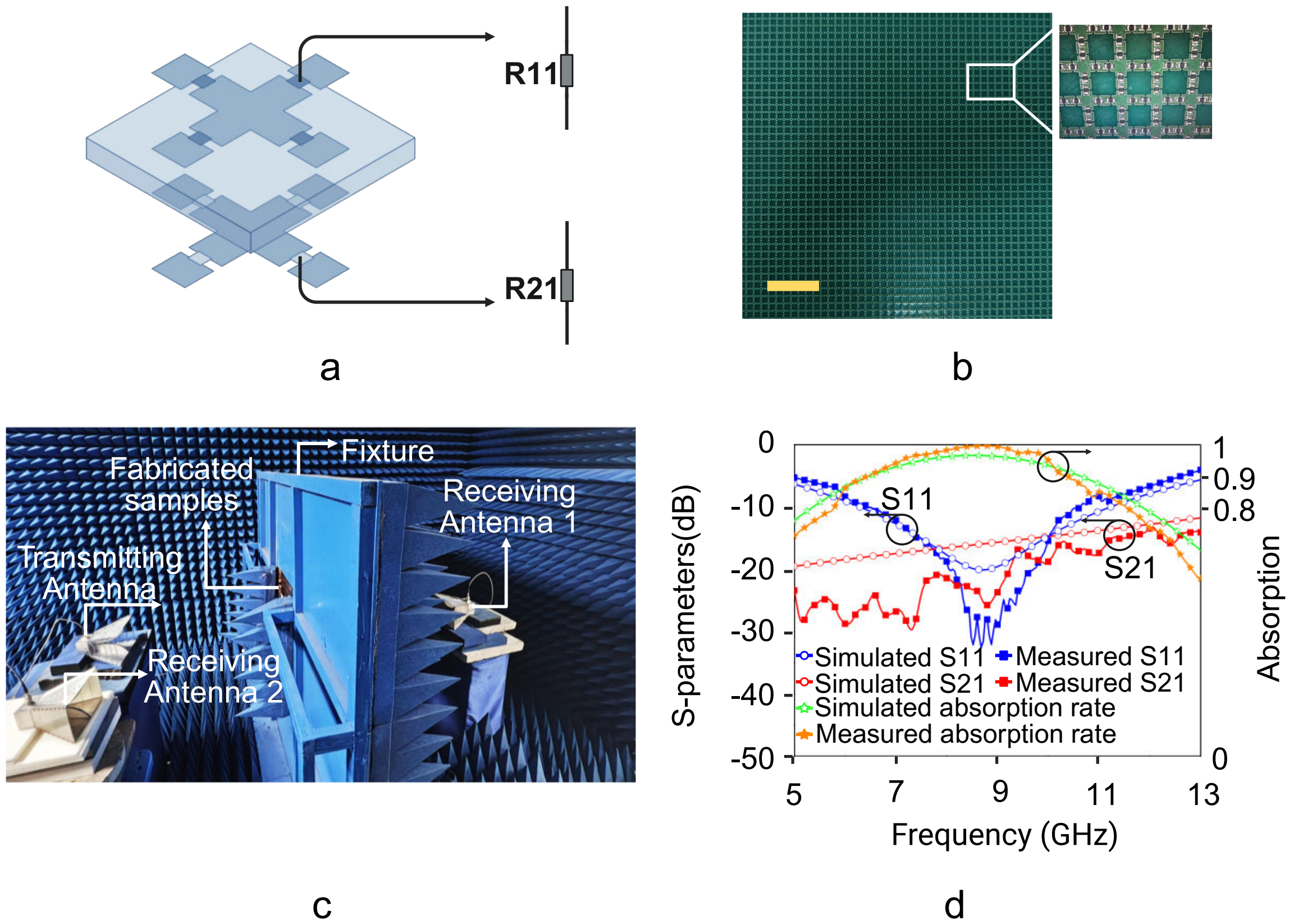

Supplementary Fig. 17: Measurement of absorber. (a) The proposed absorber based on MCES (GDCM code: {0 160 0 0 0 0 0 1 0 0 0 0 0 5}). (b)Photograph of the absorber prototype. The scale bar, 5 cm. (c)Photograph of the experimental setup. (d)Comparisons between the simulated and the measured results. Created in BioRender. XU, M. (2026) https://BioRender.com/d2gmtoc.

fabricated prototype is characterized in a microwave chamber using the free-space measurement system (see Supplementary Figure 16d). The FSS prototype is placed within a large absorptive screen with the dimensions of 1 m × 1 m. The screen has a rectangular-shaped opening, which has the same dimensions as those of the FSS. The screen is placed within two antennas connected to the two ports of a vector network analyzer (VNA). To cover the testing frequency region, three pairs of horn antennas are adopted. Details on the experimental setup can be found in [38].

The measured transmission coefficients of the fabricated FSS are shown in Supplementary Figure 16b, together with the simulated results. The passband and the wide out-of-band rejection characteristics can also be observed in the measured results. It can be observed that the metasurface now functions as an FSS, exhibiting a transmission coefficient greater than -3 dB from 8 to 12.1 GHz, less than -10 dB from 0 to 7.1 GHz and 12.8 to 35.2 GHz, and less than -20 dB from 0 to 5 GHz, 14.4 to 19.4 GHz and 20.6 to 30.6 GHz. This demonstrates a wide transmission passband and strong out-of-band suppression.

Compared with the simulation results, the passband exhibits a slight reduction in bandwidth, while the out-of-band suppression performance is significantly improved. The -10 dB out-of-band suppression bandwidth increases from 21 GHz to 29.5 GHz, and the -20 dB suppression bandwidth expands from 12.2 GHz to 20 GHz. These discrepancies primarily stem from fabrication tolerances in the FSS array and diffraction effects of EM waves during measurement. Nevertheless, despite these variations, the measured results demonstrate favorable performance. Furthermore, comparisons with similar previous works have been made and the results are listed in Supplementary Table 13. It can be found that the designed FSS structure has better passband, and wider stop-band. Considering this ideal band-pass frequency response, the designed FSS is a good candidate for radome wall.

4.3 Experimental verification of absorber based on MCES.

The absorber of the model design is shown in Supplementary Figure 17a, the structural code of GDCM is {0 160 0 0 0 0 0 1 0 0 0 0 0 5}), which means that the first response layer is composed of resistors $R_{11}(160\Omega)$, and the second response layer is $R_{21}(1\Omega)$, achieving the function of an absorber.

Supplementary Table 14: COMPARISON BETWEEN THE PROPOSED ABSORBER AND OTHER REPORTED WORK

Ref	Dimension	Thickness	BW (GHz) $\geq$ 80%	BW_{CE} (%)	Absorber type	Air gap (Y/N)
[12]	0.46λ	0.6λ	1-12.9	13.21	Symmetric	Y
[13]	0.55λ	0.24λ	2.68-12.19	16.56	Symmetric	Y
[14]	0.37λ	0.12λ	5.3-11.2	41.50	Symmetric	N
[15]	0.40λ	0.24λ	2.22-11.49	33.47	Symmetric	Y
[16]	0.50λ	0.22λ	4.6-20.45	21.95	Symmetric	Y
[17]	0.30λ	0.23λ	2.7-12.7	57.85	Symmetric	Y
[18]	0.40λ	0.06λ	2.85-5.31	53.15	Symmetric	N
[19]	0.57λ	0.22λ	3.1-14.02	17.12	Symmetric	Y
[20]	0.46λ	0.21λ	6.79-14.96	16.01	Symmetric	Y
[11]	0.22λ	0.07λ	4.72-8.49	166.78	Asymmetric	N
[14]	0.37λ	0.14λ	5.3-11.2	37.2	Symmetric	N
Proposed	0.08λ	0.14λ	5.8-11.6	697.3	Symmetric	N

- BW:Bandwidth; BW_{CE}:Cost-effective BW; Y:Yes; N:No;and—:Not reported.

The absorption can be calculated using the following equation [39]:

$$A(\omega) = 1 - R(\omega)T(\omega) \tag{22}$$

where R(ω) and T (ω) are the reflection and transmission coefficients, respectively, denoted as $|S11|^2$ and $|S21|^2$. At this point, the equivalent circuit diagram and simulation results of the corresponding GDCM are shown in Supplementary Figure 12f.

Supplementary Figure 17c shows the deployed RF setup for measuring and testing the proposed absorber. A Keysight PNA (8361C) vector network analyzer (VNA) was used to measure the complex scattering S-parameters, which were postprocessed to exper imentally validate the performance and wideband output of the absorber and to further benchmark its behavior. During the measurements, three standard gain horn antennas connected to the VNA ports were utilized: one serving as the transmitter (Tx) and two used as the receiver (Rx1,Rx2). To effectively mitigate unwanted environmental reflections, tapered wedge absorbers were placed around the device under test (DUT). The Tx horn antenna provided the normal incident waves to the prototype. Initially, the incident wave was directed toward the backplane of the absorber. These reflection and transmission coefficients are used as the basis for reflection and transmission. The reflected wave was then captured using the Rx2 horn antenna, and the transmitted wave was captured using the Rx1 horn antenna. The above stages can be considered standard procedures for testing similar EM prototypes.

The simulations and measurements exhibited similar trends for the S-parameter and absorption cases, as shown in Supplementary Figure 17d. Moreover, it can be seen from the overall spectrum results that there are reasonably good agreements between the simulations and measurements. The fabrication imperfections, soldering irregularities, non ideal testing conditions, parasitic effects of the resistors, substrate quality, and environmental noise and reflections may have also caused slight mismatches or interference between the simulated and measured results.

Furthermore, comparisons with similar previous works have been made and the results are listed in Supplementary Table 14. It can be observed that the proposed absorber structure achieves a superior absorption performance. The benefits of the developed configuration include a simple structural design, compact electrical size, smaller thickness, wider absorption bandwidth and no air layer. This resulted in a cost-effective BW of 697.3%. It utilizes only a few resistors and eliminates air gaps and spacers to produce a comparable BW in the C- and X-bands.

4.4 MCES enables standardized design of metasurfaces via micro-nano fabrication processes.

In previous inverse-designed MCES, lumped components were employed during fabrication due to the simplicity of the integrated circuits. It is important to note that lumped elements exhibit significant non-idealities at microwave and higher frequencies, including parasitic effects, frequency dependent losses, and limited self-resonant frequencies. Therefore, we carefully select high quality components whose self-resonant frequencies exceed the operating band. This component selection, combined with operating frequencies kept safely below device resonance, helps maintain good agreement between

simulation and measurement. However, this fabrication approach struggles to accommodate more complex circuit designs. In practice, lumped resistors, inductors, and capacitors exhibit significant non-idealities at microwave and higher frequencies, including parasitic inductance and capacitance, skin-effect losses, dielectric losses, and limited self-resonant frequencies. These effects can substantially degrade performance, especially when operating near or beyond the self-resonant frequency of the components.

In this work, we adopt micro-nano processes to integrate sophisticated circuits into a single standalone chip, achieving the final form of MCES physical implementation. Specifically, while maintaining the same physical structure (i.e., the basic metasurface unit), different metasurface functionalities can be realized by replacing the integrated chip, which contains the complex circuitry. Moving forward, metasurface design and fabrication will only require the design of intricate circuits, followed by micro-nano-based manufacturing. By interchanging different chips, diverse functionalities can be achieved. This approach fundamentally revolutionizes the design methodology and fabrication verification process for metasurfaces.

The design concept and experimental results of the micro-nano-based MCES are illustrated in Supplementary Figure 18. By integrating complex circuitry into a single chip, functional reconfiguration can be achieved simply by replacing the top or bottom chip layers. This approach eliminates the need for endlessly modifying intricate metasurface structures—future researchers need only redesign

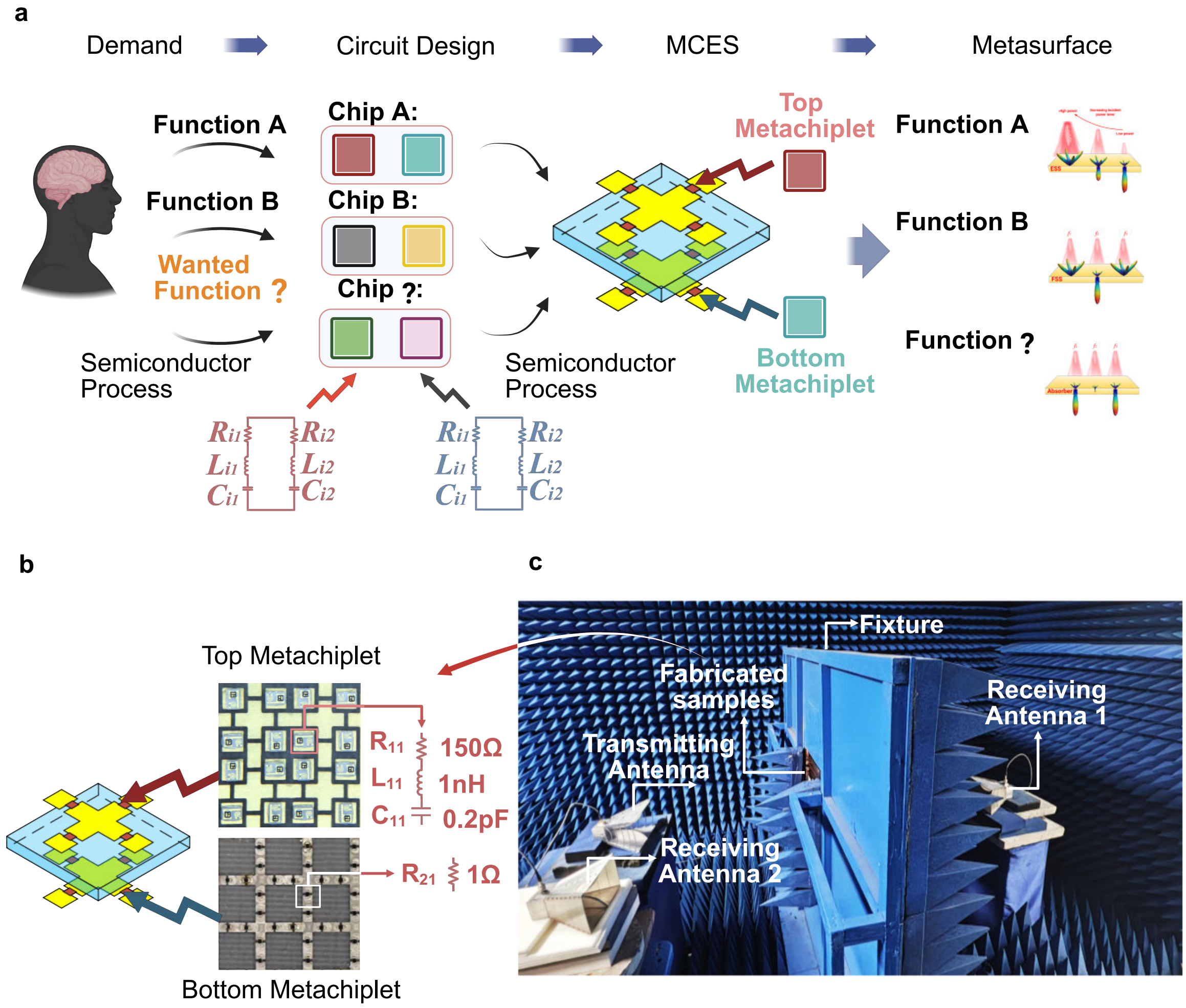

Supplementary Fig. 18: Design and measurement results of MCES based on micro-nano technology. **a**, micro-nano-based MCES implementation. Sophisticated circuits are integrated into a single chip using micro-nano processes, enabling diverse metasurface functionalities without structural modification. Different electromagnetic responses are achieved by interchanging integrated circuit chips, while maintaining a uniform metasurface geometry. This approach transforms metasurface design: future developments require only circuit redesign and standard micro-nano fabrication, eliminating the need for complex structural modifications and streamlining both design methodology and experimental validation. **b,c**, Photographs of the fabricated absorber sample and free-space test method. Created in BioRender. XU, M. (2026) https://BioRender.com/5bgyvx2.

the integrated circuits within the chip to realize diverse metasurface functionalities. It is important to note that the implementation of metachiplets must consider process limitations. For example, resistance value and area are interrelated. Nichrome (NiCr) and tantalum nitride (TaN), with sheet resistances of 50 and 100 $\Omega/\square$, are commonly used to produce resistors. The maximum resistance for a single resistor is 500 Ω with dimensions of 0.05 mm by 0.5 mm. Larger resistance values can be achieved by connecting multiple resistors in series. Therefore, during the design process, we typically require resistance values to be below 500 Ω.

As demonstrated in our fabricated absorber, the top chip incorporates a series RLC (R_{11} = 150Ω, L_{11} = 1nH, R_{11} = 0.2pF) network, while the bottom chip contains a single resistor(R_{21} = 1Ω). Notably, the chip is relatively large compared to lumped components. To adapt micro-nano processes for the MCES implementation, we increased the unit cell size from 2.8 mm to 4 mm. This further validates our earlier discussion that the essence of the MCES concept lies in "simple structure + complex circuitry". Importantly, MCES can be implemented using various unit cell and is not limited to the specific structure proposed in this work. The device was characterized using the method depicted in Supplementary Figure 18b, yielding measured S_{11} and S_{21}. The absorption rate, derived from these measurements, is compared with the simulated results in Supplementary Figure 18(c). The absorber exhibits >80% absorption from 4.5 to 11.6 GHz and >90% absorption between 5.6 and 10.3 GHz. While a slight frequency shift is observed relative to simulations, the S-parameter trends remain consistent, with discrepancies likely attributable to fabrication tolerances or measurement uncertainties. Compared to lumped-component-based absorbers, the micro-nano-based design achieves a significant improvement in absorption bandwidth (for > 80% absorption) from 67% to 88.2%, while reducing thickness from $0.13\lambda_0$ to $0.1\lambda_0$ and increasing the BW_{CE} from 697.3 to 713.3.

4.5 Micro-Nano Fabrication for Parasitic Effect Minimization.

This supplementary note provides a detailed explanation of how micro-nano fabrication minimizes parasitic effects and offers a quantitative comparison with traditional PCB-based fabrication processes.

Micro-nano fabrication minimizes parasitic effects through extreme miniaturization, precise dimensional control, monolithic integration, and advanced materials. First, extreme miniaturization reduces the physical distances between circuit elements, which directly lowers the parasitic inductance and capacitance associated with interconnects. Additionally, precise dimensional control via lithographic techniques minimizes process induced variations, ensuring consistency between fabrication and design. Furthermore, monolithic integration eliminates the need for discrete component packaging, removing parasitic contributions from bond wires, lead frames, and encapsulation materials. Finally, advanced materials such as high-resistivity substrates and thick metal layers further reduce substrate losses and dielectric absorption. These combined factors result in significantly reduced parasitic effects, which improves the accuracy of circuit models and enhances agreement between simulation and measurement, particularly at higher frequencies where parasitics play a dominant role. The self-resonant frequencies of integrated passive devices fabricated using this approach can exceed the Ka-band [40–43], making them well suited for high-frequency applications.

To demonstrate these advantages quantitatively, we compare key parameters with conventional PCB processes. In PCB implementations using surface-mount components, each interconnect contributes parasitic inductance and capacitance arising from pads, traces, and packaging. For example, the Murata GQM1875C2ER10BB12 capacitor has an equivalent series inductance of 0.371 nH, while the Skyworks SMP1340-007LF PIN diode introduces approximately 0.4 nH of parasitic inductance from its lead frame and bond wires. Similarly, the Murata LQG15HH1N0B02 inductor shows a parasitic capacitance of approximately 0.1 pF from its internal winding structure. When combined with additional parasitics from PCB pads and interconnects, the total series inductance typically falls within 0.2–1 nH and the total shunt capacitance within 0.1–0.3 pF. In contrast, micro-nano integration reduces interconnect parasitics to negligible levels. Component self-resonant frequencies increase from around 20 GHz for discrete parts (e.g., the Murata LQP03TG0N1B02) to exceed the Ka-band for integrated passives. Moreover, micro-nano fabrication integrates RLC networks within a footprint below 1 mm^2, rather than the 1–2 mm^2 required by surface-mount processes, achieving a level of circuit complexity and integration density that the latter cannot match. The use of packaged components at high frequencies often introduces significant performance degradation due to these parasitic effects. In contrast, micro-nano integration minimizes such parasitics, ensuring better agreement between simulated and measured responses.

4.6 Cost Comparison between Micro-Nano Fabrication and Conventional Methods

To better illustrate the cost, we obtained pricing information from commercial foundries. Based on quotes from commercial foundries, the fabrication cost is approximately 0.23 USD per metachiplet (calculated from 1,390 USD for 6,000 units). For comparison, the total cost of discrete components for a series RLC network is approximately 0.24 USD, including a resistor (RT0201FRE07150RL at 0.023 USD), an inductor (Murata LQW03AW8N2J00D at 0.075 USD), and a capacitor (Murata GQM1875C2ER10BB12 at 0.138 USD). This shows that the metachiplet cost is already slightly lower than the combined discrete component cost, without considering additional assembly expenses.

In the research and development phase, rapid iteration without mask costs offers significant time savings over conventional PCB processes. Traditional PCB approaches require weeks per iteration with new mask fabrication. In contrast, design errors in our workflow can be corrected by simply updating the design file and re-exposing the next day, cutting iteration time from weeks to days. When amortized across multiple design cycles and production runs, the overall cost becomes increasingly favorable.

At large-scale production, the cost advantage becomes increasingly clear. A single run produces tens of thousands of metachiplets, and the same design can be reused across multiple projects with no additional development cost. Considering the full workflow from design to production, the overall cost is acceptable, especially when performance and design flexibility are priorities. Rapid iteration during development combined with scalable production costs makes the MCES framework a practical solution for real-world engineering applications.

Supplementary References

[1] Zhao, P.-C., Zong, Z.-Y., Wu, W., Li, B., Fang, D.-G.: Miniaturized-element bandpass fss by loading capacitive structures. IEEE Transactions on Antennas and Propagation **67**(5), 3539–3544 (2019) https://doi.org/10.1109/TAP.2019.2902633

[2] Yu, Y.-M., Chiu, C.-N., Chiou, Y.-P., Wu, T.-L.: An effective via-based frequency adjustment and minimization methodology for single-layered frequency-selective surfaces. IEEE Transactions on Antennas and Propagation **63**(4), 1641–1649 (2015) https://doi.org/10.1109/TAP.2015.2398123

[3] Zhao, P.-C., Zong, Z.-Y., Wu, W., Li, B., Fang, D.-G.: An fss structure based on parallel lc resonators for multiband applications. IEEE Transactions on Antennas and Propagation **65**(10), 5257–5266 (2017) https://doi.org/10.1109/TAP.2017.2735461

[4] Dey, S., Dey, S.: Miniaturized near all-angle stable 2.5-d fss for dual-band shielding in transmission mode and multiband absorption in reflection mode. IEEE Transactions on Antennas and Propagation **71**(9), 7323–7332 (2024) https://doi.org/10.1109/TAP.2023.3291816

[5] Zhou, L., Liu, L., Shen, Z.: High-performance energy selective surface based on the double-resonance concept. IEEE Transactions on Antennas and Propagation **69**(11), 7658–7666 (2021) https://doi.org/10.1109/TAP.2021.3075548

[6] Hu, N., Wang, K., Zhang, J., Zha, S., Wu, Z., Liu, C., Liu, P.: Design of ultrawideband energy-selective surface for high-power microwave protection. IEEE Antennas and Wireless Propagation Letters **18**(4), 669–673 (2019) https://doi.org/10.1109/LAWP.2019.2900760

[7] Wu, Z., Xu, Y., Liu, P., Tian, T., Lin, M.: An ultra-broadband energy selective surface design method: From filter circuits to metamaterials. IEEE Transactions on Antennas and Propagation **71**(7), 5865–5873 (2023) https://doi.org/10.1109/TAP.2023.3276447

[8] Tian, T., Huang, X., Xu, Y., Liu, P., Liu, C., Hu, N., Zhang, J., Wu, Z.: A wideband energy selective surface with quasi-elliptic bandpass response and high-power microwave shielding. IEEE Transactions on Electromagnetic Compatibility **66**(1), 224–233 (2024) https://doi.org/10.1109/TEMC.2023.3325438

[9] Jiang, Y., Tian, L., Jiang, Z.H., Hong, W.: Analysis and design of ultrathin filtering energy-selective surfaces with ultrawideband and high shielding effectiveness. IEEE Transactions on Antennas and Propagation **73**(1), 641–646 (2025) https://doi.org/10.1109/TAP.2024.3464876

[10] Zhong, S., Chen, M., Xu, G., Chen, Y.: Dual-band energy-selective surface with enhanced independence based on two-layer diode-loaded fss. IEEE Transactions on Antennas and Propagation **72**(6), 5011–5020 (2024) https://doi.org/10.1109/TAP.2024.3391392

[11] Islam, K.M.R., Rahimian, A., Machado, G.G., Ali Babar Abbasi, M., Ahmad Cheema, A., Meenan, B.J.: Design and experimental performance evaluation of a single-layer polarization-insensitive asymmetric microwave metasurface absorber. IEEE Transactions on Antennas and Propagation **72**(8), 6520–6529 (2024) https://doi.org/10.1109/TAP.2024.3424952

[12] Yao, Z., Xiao, S., Li, Y., Wang, B.-Z.: On the design of wideband absorber based on multilayer and multiresonant fss array. IEEE Antennas and Wireless Propagation Letters **20**(3), 284–288 (2021) https://doi.org/10.1109/LAWP.2020.3046010

[13] Zhang, B., Jin, C., Shen, Z.: Low-profile broadband absorber based on multimode resistor-embedded metallic strips. IEEE Transactions on Microwave Theory and Techniques **68**(3), 835–843 (2020) https://doi.org/10.1109/TMTT.2019.2956933

[14] Kundu, D., Mohan, A., Chakrabarty, A.: Single-layer wideband microwave absorber using array of crossed dipoles. IEEE Antennas and Wireless Propagation Letters **15**, 1589–1592 (2016) https:

//doi.org/10.1109/LAWP.2016.2517663

[15] Yao, Z., Xiao, S., Jiang, Z., Yan, L., Wang, B.-Z.: On the design of ultrawideband circuit analog absorber based on quasi-single-layer fss. IEEE Antennas and Wireless Propagation Letters **19**(4), 591–595 (2020) https://doi.org/10.1109/LAWP.2020.2972919

[16] Vadakkekalathil Abdulhakim, L.M., Aanandan, C.K.: A novel polarization independent wideband circuit analog absorber using crossed loops. Radioengineering **27**(3), 738–745 (2018) https://doi.org/10.13164/re.2018.0738

[17] Zhang, Y., Yang, W., Li, X., Liu, G.: Design and analysis of a broadband microwave metamaterial absorber. IEEE Photonics Journal **15**(3), 1–10 (2023) https://doi.org/10.1109/JPHOT.2023.3277449

[18] Yuan, W., Cheng, Y.: Low-frequency and broadband metamaterial absorber based on lumped elements: design, characterization and experiment. Applied Physics A-materials Science & Processing **117**(4), 1915–1921 (2014) https://doi.org/10.1007/s00339-014-8637-3

[19] Zhang, M., Zhang, B., Liu, X., Sun, S., Jin, C.: Design of wideband absorber based on dual-resistor-loaded metallic strips. International Journal of Antennas and Propagation **2020**(1), 1238656 (2020) https://doi.org/10.1155/2020/1238656

[20] Yoo, M., Lim, S.: Polarization-independent and ultrawideband metamaterial absorber using a hexagonal artificial impedance surface and a resistor-capacitor layer. IEEE Transactions on Antennas and Propagation **62**(5), 2652–2658 (2014) https://doi.org/10.1109/TAP.2014.2308511

[21] Sung, G.H.-h., Sowerby, K.W., Neve, M.J., Williamson, A.G.: A frequency-selective wall for interference reduction in wireless indoor environments. IEEE Antennas and Propagation Magazine **48**(5), 29–37 (2006) https://doi.org/10.1109/MAP.2006.277152

[22] Yao, Z., Xiao, S., Wang, B.-Z.: Study on an accurate and efficient design method of resonant fsss based on the macro-model of units in the basic strip-gap fss. IEEE Transactions on Antennas and Propagation **69**(5), 2741–2750 (2021) https://doi.org/10.1109/TAP.2020.3027514

[23] Li, Z., Weng, X., Li, Y., Yi, X., Liao, M., Bi, M.: Reconfigurable active fss with switchable passband/stopband response. IEEE Trans. Antennas Propag. **74**(3), 2566–2577 (2026) https://doi.org/10.1109/TAP.2025.3650638

[24] Liu, N., Sheng, X., Zhang, C., Guo, D.: Design and synthesis of band-pass frequency selective surface with wideband rejection and fast roll-off characteristics for radome applications. IEEE Trans. Antennas Propag. **68**(4), 2975–2983 (2020) https://doi.org/10.1109/TAP.2019.2955665

[25] Han, Y., Liang, M., Ji, Q., Li, Q., Che, W.: Low-profile and wideband paper-based honeycomb microwave absorber with capacitive loops. IEEE Trans. Antennas Propag. **73**(6), 3757–3766 (2025) https://doi.org/10.1109/TAP.2025.3542585

[26] Islam, K.M.R., Rahimian, A., Machado, G.G., Abbasi, M.A.B., Cheema, A.A., Meenan, B.J.: Design and experimental performance evaluation of a single-layer polarization-insensitive asymmetric microwave metasurface absorber. IEEE Trans. Antennas Propag. **72**(8), 6520–6529 (2024) https://doi.org/10.1109/TAP.2024.3424952

[27] Ning, Z., Li, M., Chen, L., Gu, J., Ding, D.: Circuit modeling and analysis of multiband energy-selective surfaces using characteristic modes. IEEE Trans. Antennas Propag. **73**(10), 7583–7595 (2025) https://doi.org/10.1109/TAP.2025.3575579

[28] Zhou, L., Liu, L., Shen, Z.: High-performance energy selective surface based on the double-resonance concept. IEEE Trans. Antennas Propag. **69**(11), 7658–7666 (2021) https://doi.org/10.1109/TAP.2021.3075548

[29] Qu, M., He, Z., Su, J., Nayyeri, V.: A rasorber with energy-selective passband and ultrawideband absorptive out-of-band. IEEE Trans. Microw. Theory Techn. **72**(10), 5643–5652 (2024) https:

//doi.org/10.1109/TMTT.2024.3387917

[30] Ebrahimi, A., Shen, Z., Withayachumnankul, W., Al-Sarawi, S.F., Abbott, D.: Varactor-tunable second-order bandpass frequency-selective surface with embedded bias network. IEEE Transactions on Antennas and Propagation **64**(5), 1672–1680 (2016) https://doi.org/10.1109/TAP.2016.2537378

[31] Zhou, Q., Guo, M., Moghadas, H., Wu, Z., Liu, P., Daneshmand, M.: A frequency selective rasorber with three transmission bands and three absorption bands. IEEE Access **7**(1), 160973–160981 (2019) https://doi.org/10.1109/ACCESS.2019.2951503

[32] Jin, C., Lv, Q., Zhang, B., Liu, J., An, S., He, Z.S., Shen, Z.: Ultra-wide-angle bandpass frequency selective surface. IEEE Transactions on Antennas and Propagation **69**(9), 5673–5681 (2021) https://doi.org/10.1109/TAP.2021.3061144

[33] Yang, C., Liu, P.-G., Huang, X.-J.: A novel method of energy selective surface for adaptive hpm/emp protection. IEEE Antennas and Wireless Propagation Letters **12**(1), 112–115 (2013) https://doi.org/10.1109/LAWP.2013.2243105

[34] Liu, N., Sheng, X., Zhang, C., Guo, D.: Design and synthesis of band-pass frequency selective surface with wideband rejection and fast roll-off characteristics for radome applications. IEEE Transactions on Antennas and Propagation **68**(4), 2975–2983 (2020) https://doi.org/10.1109/TAP.2019.2955665

[35] Wu, B., Li, H.-L., Zhao, Y.-T., Wang, Y.-L., Lu, W.-B., Chen, L.: Design and validation of flexible multilayer frequency selective surface with transmission zeros. IEEE Antennas and Wireless Propagation Letters **18**(2), 250–254 (2019) https://doi.org/10.1109/LAWP.2018.2888523

[36] Yepes, C., Cavallo, D., Gandini, E., Monni, S., Neto, A., Vliet, F.E.: Angularly stable frequency selective surface combined with a wide-scan phased array. IEEE Transactions on Antennas and Propagation **66**(2), 1046–1051 (2018) https://doi.org/10.1109/TAP.2017.2778768

[37] Afzal, W., Ebrahimi, A., Robel, M.R., Rowe, W.S.T.: Low-profile higher-order narrowband bandpass miniaturized-element frequency-selective surface. IEEE Transactions on Antennas and Propagation **71**(4), 3736–3740 (2023) https://doi.org/10.1109/TAP.2023.3239171

[38] Momeni Hasan Abadi, S.M.A., Li, M., Behdad, N.: Harmonic-suppressed miniaturized-element frequency selective surfaces with higher order bandpass responses. IEEE Transactions on Antennas and Propagation **62**(5), 2562–2571 (2014) https://doi.org/10.1109/TAP.2014.2303822

[39] Luo, Z., Ji, S., Zhao, J., Dai, H., Jiang, C.: Design and analysis of an ultra-thin dual-band wide-angle polarization-insensitive metamaterial absorber for c-band application. Optik **243**, 166785 (2021) https://doi.org/10.1016/j.ijleo.2021.166785

[40] Zeng, Y., Wu, Y., Yang, Y., Pan, L., Wang, W.: A novel minimized millimeter-wave on-chip spoof surface plasmon polariton and its applications based on ipd technology. Microelectron. J. **162**, 106755 (2025) https://doi.org/10.1016/j.mejo.2025.106755

[41] Li, Y., Ni, J., Li, S., Han, Q., Tian, J., Li, N., Ma, T.: Design of a millimeter-wave wideband filtering balun in gaas ipd technology. Front. Phys. **13**, 1555410 (2025) https://doi.org/10.3389/fphy.2025.1555410

[42] Ji, N., Shen, G., Feng, W., Xue, Q., Che, W.: Packaged ka-band ipd bandpass filter using in-phase and out-of-phase mixed couplings. IEEE Trans. Compon. Packag. Manuf. Technol. **15**(8), 1750–1758 (2025) https://doi.org/10.1109/TCPMT.2025.3581320

[43] Shen, G., Wen, B., He, Z., Ji, N., Feng, W., Che, W.: A 3.5-/28-ghz dual-band ipd bandpass filter using gaas process for 5g application. IEEE Microw. Wireless Technol. Lett. **35**(6), 686–689 (2025) https://doi.org/10.1109/LMWT.2025.3552089
