# Supplementary material for: Large-scale data-driven inverse EM design based on metacircuit-embedded surface
Source: Nat Commun. 2026 Jul 13;17:8603. doi: 10.1038/s41467-026-75411-z (PMC13486762; doi:10.1038/s41467-026-75411-z)
Supplement: Supplementary file 2 [file 41467_2026_75411_MOESM2_ESM.pdf]

nature portfolio

Peer Review File

Large-Scale Data-Driven Inverse EM Design Based on Metacircuit-Embedded Surface

Corresponding Author: Professor Song Zha

This file contains all reviewer reports in order by version, followed by all author rebuttals in order by version.

Version 0:

Reviewer comments:

Reviewer #1

(Remarks to the Author)
Review Comments
This paper proposes a metacircuit-embedded surface (MCES) design framework, which transfers the design focus from geometric structures to metacircuits. By replacing full-wave simulations with circuit simulations, it enables the rapid generation of large-scale training data. Using a double-layer cross-element as the basic structure, the authors generated millions of samples within days, and built an AI-driven metasurface model based on these data, forming a complete forward prediction and inverse design pipeline. This framework can modulate the metasurface response in the frequency, amplitude, phase, and polarization domains, with its performance validated through electromagnetic (EM) simulations and experimental measurements. However, the authors' claim that this work lays the foundation for general-purpose EM large model still requires further justification.
1.The authors claim that data generated by rapid circuit simulations can pave the way for the efficient construction of EM large model. However, this claim currently lacks sufficient experimental verification. The manuscript does not provide a quantitative comparison between ADS circuit simulation results and CST full-wave EM simulation results; thus, the accuracy of the mapping relationship between them cannot be evaluated. It should be noted that the response obtained from circuit simulation is not the EM solution derived from Maxwell's equations. Whether this response can be termed an EM response, and whether a model trained on such data can be called an "EM large model", remains debatable. Reliance solely on circuit simulation data is insufficient to serve as a solid data foundation for constructing EM models. Furthermore, the inverse design of EM structures using circuit simulation data is theoretically not rigorous, as the input–output relationship cannot truly reflect full-wave EM behavior. The authors realize various metasurface functions by designing multiple metacircuits; however, it should be emphasized that ADS circuit simulation only models idealized circuit responses. Actual passive components (e.g., capacitors, inductors) typically exhibit strong frequency dependence, and may even show nonlinear or dispersive effects under certain conditions. Therefore, the authors are requested to explicitly clarify whether the real characteristics of components (e.g., frequency responses from datasheets, equivalent circuit models, or S-parameter models) were imported into ADS to ensure that the circuit simulation results reasonably approximate the actual EM behavior of physical devices.
2.The title of this paper is "Efficient EM Large Model Framework Based on Metacircuit-Embedded Surface". However, based on the content, the forward model adopts a conventional fully connected neural network (FCNN) with an input dimension of 14, hidden layers (64–256–512–1024), and an output dimension of 800 to predict responses from circuit parameters. For inverse design, the authors mainly rely on traditional optimization algorithms rather than generative models, differentiable inverse frameworks, or more advanced large-model methods with strong generalization capabilities. Although about 6 million data samples were used, which can be considered a large-scale dataset for EMs-driven inverse design, the definition of a "large model" is not solely determined by data volume. The adopted FCNN consists of 1 input layer, 4 hidden layers, and 1 output layer, whose structural complexity and parameter scale are significantly lower than those typically associated with modern "large models" (i.e., deep architectures, huge parameter counts, or pretraining capabilities). In addition, both forward prediction and inverse design rely on conventional methods without demonstrating architectural innovations, pretraining–fine-tuning pipelines, or cross-task generalization expected from large models. In summary, the current work is more appropriately described as "inverse design based on large-scale data" rather than a genuine "EM large model framework". The authors are suggested to revisit the use of the term "Large Model" in the title and throughout the manuscript, or further improve the model architecture, parameter scale, and inverse-design methodology to justify the "large model" claim.
3.In the introduction, the authors state that micro-nano fabrication can minimize parasitic effects, but they do not further

explain the underlying mechanism or provide a comparison with traditional fabrication processes in the main text.
4.The authors state that mapping the high-dimensional discontinuous geometric domain to a low-dimensional continuous circuit-parameter space significantly reduces optimization complexity. However, for conventional geometric designs (not discretization-based topology optimization), geometric parameters themselves are continuous variables. The authors should provide a clear explanation.
5.The proposed MCES framework can generate millions of data samples within a short time via ADS circuit simulation for designing metasurfaces with diverse performances, thus improving model generalization. This does not mean that conventional EM modeling cannot achieve a similar data scale. In fact, traditional EM modeling can also obtain large-scale datasets with various performance metrics via full-wave simulations of numerous structures. The main difference lies in data acquisition efficiency: the advantage of MCES is that it drastically reduces single-simulation time and thus generates large-scale data more efficiently under the same computational resources, rather than invalidating the ability of traditional EM simulation to produce massive data.
6.The paper mentions that metasurfaces with different functions can be realized by modifying the metacircuit design. However, in engineering applications, redesigning and fabricating different circuit structures via micro-nano processes for each function usually leads to much higher fabrication complexity and cost compared with conventional metal-patch-based structures for the same performance. The authors should further clarify the advantages of the proposed method in terms of practical manufacturing cost, scalability, and engineering feasibility.
7.The authors claim that large-scale datasets can reduce the requirements for AI training. This statement needs further clarification. Large datasets do help improve generalization and reduce overfitting, which may relax requirements on model structure or regularization. Nevertheless, large datasets do not reduce the demand for computational resources during training, and excessive low-quality data may even degrade training performance. The authors should use a more accurate expression.
8.Regarding the dataset size: ADS circuit simulation is computationally cheap, and the authors used a million-level dataset for model training, but the justification for this scale is insufficient. Generally, circuit models with clear physical mechanisms do not require such a huge amount of data. Supplementary Fig. S11 shows the MSE versus training epochs and the generalization performance under different dataset sizes, but the authors did not analyze the implications of these results. A systematic sensitivity analysis is suggested based on the provided curves:
- Does model performance saturate beyond a certain data size?
- Is there obvious redundancy in the million-level data?
- Does the improvement in accuracy/generalization exhibit diminishing returns with increasing data volume?
- Can a minimum acceptable data range be provided?
In addition, the authors should clarify whether the generalization improvement from massive data has practical engineering significance.
9.In Fig. S12, the authors present inversely designed metasurface structures validated by full-wave simulation and measurement. However, all training data were obtained from ADS circuit simulations. The authors should further clarify: during inverse design, was the model optimized to match the response from ADS circuit simulation, which was then verified by CST full-wave simulation?
10.The authors achieved fast design of diverse metasurfaces via classical optimization algorithms but did not specify the exact algorithm used. From the term “particle swarm size”, it can be inferred that particle swarm optimization (PSO) was likely adopted. The authors are suggested to explicitly name the optimization algorithm, briefly introduce its principle, and describe its detailed implementation in the design flow. Furthermore, please explain why PSO was chosen over other popular global optimization methods (e.g., genetic algorithm (GA), differential evolution (DE), simulated annealing (SA)) and provide a comparative analysis. Performance metrics such as convergence speed and solution stability are strongly recommended to verify the advantages and rationality of PSO for this inverse design task.
11.The authors attribute the frequency shift between ESS prediction and simulation to the use of a wider frequency band. The Interpolating Solver in HFSS sets meshes according to the highest simulated frequency; thus, a large difference in the upper frequency can lead to different meshes and discrepancies in EM responses. However, ADS circuit simulation is based on circuit equations (mainly dependent on RLC parameters), where the difference between narrowband and broadband simulation is often negligible. The authors should provide validation or further discussion on this point.
12.I have some minor issues listed below:
a) Please verify the DOIs in the references. Certain links, such as references [30] and [31], are currently broken.
b) It is recommended to add scale bars to prototype images (e.g., Fig. 4) to clearly indicate physical dimensions.
13. there are some typo error remains in the manuscript. such as in line 110, the title words"framework" is not correct

Reviewer #2

(Remarks to the Author)
The manuscript is clearly written, and the presented results are of strong interest to the metasurface design community. The proposed framework represents an important step toward scalable and large-scale metasurface modeling.

However, the core methodology relies on metacircuits composed of lumped elements to achieve the desired electromagnetic functionality. This raises a fundamental concern regarding high-frequency operation. In practice, lumped resistors, inductors, and capacitors exhibit significant non-idealities at microwave and higher frequencies, including parasitic inductance and capacitance, skin-effect losses, dielectric losses, and limited self-resonant frequencies. These effects can substantially degrade performance, especially when operating near or beyond the self-resonant frequency of the components.

The reported agreement between simulation and measurement at high frequencies appears somewhat optimistic in this context. Since the experimental prototypes are small and waveguide-integrated, they contain only a limited number of meta-

unit cells and are subject to controlled boundary conditions. This configuration may not fully capture the cumulative loss mechanisms, parasitic coupling, and fabrication tolerances that would arise in a larger, free-space metasurface implementation. It would therefore strengthen the manuscript to clarify how the proposed approach scales to electrically large apertures and whether distributed effects or increased loss would compromise performance.

Additionally, some of the optimized lumped element values appear relatively high (e.g., 150-Ω resistors). Realizing such values in micro-/nano-fabrication platforms—particularly in thin-film or integrated implementations—can be challenging due to sheet resistance limitations, area constraints, and process variability. The manuscript would benefit from a discussion of fabrication feasibility and tolerances, especially if the long-term goal is integration beyond standard PCB processes.

Fully PCB-based metasurfaces are attractive precisely because they are comparatively low-loss, scalable, and fabrication-friendly. Introducing lumped elements may undermine these advantages unless the associated loss mechanisms and practical implementation constraints are carefully addressed.

A more detailed analysis of component non-idealities, Q-factors, parasitics, and scalability would significantly strengthen the technical credibility of the proposed approach.

Some works missing from the references include:
- "A Generative Machine Learning-Based Approach for Inverse Design of Multilayer Metasurfaces," in IEEE Transactions on Antennas and Propagation, vol. 69, no. 9, pp. 5725-5739, Sept. 2021, doi: 10.1109/TAP.2021.3060142

-"Synthesis of multi-band reflective polarizing metasurfaces using a generative adversarial network"

Reviewer #3

(Remarks to the Author)
The authors gave a positive reply to the reviewers' comments. Some new analyses have been done and added to the revised manuscript. It is recommended for publication in present form.

Version 1:

Reviewer comments:

Reviewer #2

(Remarks to the Author)
All my comments are addressed by the authors. I have no further comments. Thank you.

(Remarks on code availability)
The source code and data set are available through the provided link.

Open Access This Peer Review File is licensed under a Creative Commons Attribution 4.0 International License, which permits use, sharing, adaptation, distribution and reproduction in any medium or format, as long as you give appropriate credit to the original author(s) and the source, provide a link to the Creative Commons license, and indicate if changes were made.
In cases where reviewers are anonymous, credit should be given to 'Anonymous Referee' and the source.
The images or other third party material in this Peer Review File are included in the article's Creative Commons license, unless indicated otherwise in a credit line to the material. If material is not included in the article's Creative Commons license and your intended use is not permitted by statutory regulation or exceeds the permitted use, you will need to obtain permission directly from the copyright holder.
To view a copy of this license, visit https://creativecommons.org/licenses/by/4.0/

Response to Reviewers' Comments for Original Manuscript ID NCOMMS-26-005356-T

Large-Scale Data-Driven Inverse EM Design Based on Metacircuit-Embedded Surface

Responses to Comments for publication to
NATURE COMMUNICATIONS

by

Ming Xu, Song Zha, Mingtuan Lin, Hanqing Liu, Jihong Zhang, Yanlin Xu,
Huan Jiang, Qiang Cheng, and Peiguo Liu

May 1, 2026

Nature Communications
Original Manuscript ID NCOMMS-26-005356-T

Authors' Response to Reviewer 1

We would like to thank the reviewer for the valuable and constructive comments. We have revised the paper in line with the reviewer's comments, thereby improving the clarity of the paper accordingly.

Comment 1: *The authors claim that data generated by rapid circuit simulations can pave the way for the efficient construction of EM large model. However, this claim currently lacks sufficient experimental verification. The manuscript does not provide a quantitative comparison between ADS circuit simulation results and CST full-wave EM simulation results; thus, the accuracy of the mapping relationship between them cannot be evaluated. It should be noted that the response obtained from circuit simulation is not the EM solution derived from Maxwell's equations. Whether this response can be termed an EM response, and whether a model trained on such data can be called an "EM large model", remains debatable. Reliance solely on circuit simulation data is insufficient to serve as a solid data foundation for constructing EM models. Furthermore, the inverse design of EM structures using circuit simulation data is theoretically not rigorous, as the input–output relationship cannot truly reflect full-wave EM behavior. The authors realize various metasurface functions by designing multiple metacircuits; however, it should be emphasized that ADS circuit simulation only models idealized circuit responses. Actual passive components (e.g., capacitors, inductors) typically exhibit strong frequency dependence, and may even show nonlinear or dispersive effects under certain conditions. Therefore, the authors are requested to explicitly clarify whether the real characteristics of components (e.g., frequency responses from datasheets, equivalent circuit models, or S-parameter models) were imported into ADS to ensure that the circuit simulation results reasonably approximate the actual EM behavior of physical devices.*

Comment 1.1: *The authors claim that data generated by rapid circuit simulations can pave the way for the efficient construction of EM large model. However, this claim currently lacks sufficient experimental verification. The manuscript does not provide a quantitative comparison between ADS circuit simulation results and CST full-wave EM simulation results; thus, the accuracy of the mapping relationship between them cannot be evaluated. It should be noted that the response obtained from circuit simulation is not the EM solution derived from Maxwell's equations. Whether this response can be termed an EM response, and whether a model trained on such data can be called an "EM large model", remains debatable. Reliance solely on circuit simulation data is insufficient to serve as a solid data foundation for constructing EM models. Furthermore, the inverse design of EM structures using circuit simulation data is theoretically not rigorous, as the input–output relationship cannot truly reflect full-wave EM behavior.*

Response: We sincerely thank the reviewer for raising these important and insightful questions regarding the validity of using circuit-simulated data as a foundation for electromagnetic modeling, as well as the quantitative agreement between ADS circuit simulations and CST full-wave simulations. We appreciate the opportunity to clarify these points, which are central to the metacircuit-embedded surface (MCES) framework.

Our training data generation does not rely solely on pure circuit simulations. The MCES framework employs a field-circuit co-simulation approach that integrates both full-wave and circuit solvers. First, a single full-wave simulation (CST) extracts the intrinsic S-parameters of the fixed topology (e.g., the double-layer cross-shaped metallic frame). These S-parameters are then imported into ADS as the "field" component and co-simulated with the "circuit" component consisting of lumped elements. Therefore, the generated training data are fundamentally electromagnetic responses that genuinely reflect full-wave behavior, not merely circuit-equation solutions. This critical detail has been explicitly clarified in the revised manuscript (Page 10, Lines 320-331).

Revisions have been made as follows:

> **Manuscript:**
>
> Methods
>
> *Training data generation*
>
> *The training dataset for the MCES framework was generated using a field-circuit co-simulation approach in ADS. First, the S-parameters of the fixed metasurface topology (e.g., the double-layer cross-shaped metallic structure) were extracted through a single full-wave simulation in CST Microwave Studio. These S-parameters fully capture the electromagnetic behavior of the passive frame, including its frequency-dependent transmission, reflection, and loss characteristics. Subsequently, the extracted S-parameters were imported into ADS as a frequency-dependent component and co-simulated with lumped-element circuits comprising resistors (R), inductors (L), capacitors (C), and diodes. This field-circuit co-simulation approach ensures that the generated data inherently account for the full-wave electromagnetic behavior of the fixed structure while maintaining the computational efficiency required for large-scale dataset generation*....

Following the reviewer's suggestion, we have added a systematic quantitative comparison between ADS field-circuit co-simulation and CST full-wave simulation. For a randomly selected metacircuit, the S-parameter responses from both methods were compared. The results show excellent agreement across the entire frequency band (0.1–40 GHz), with near-perfect overlap between the curves (Fig. 1). This quantitative validation confirms that data generated through field-circuit co-simulation are highly consistent with full-

wave simulation results, and their accuracy is sufficient to serve as a reliable foundation for constructing electromagnetic models.

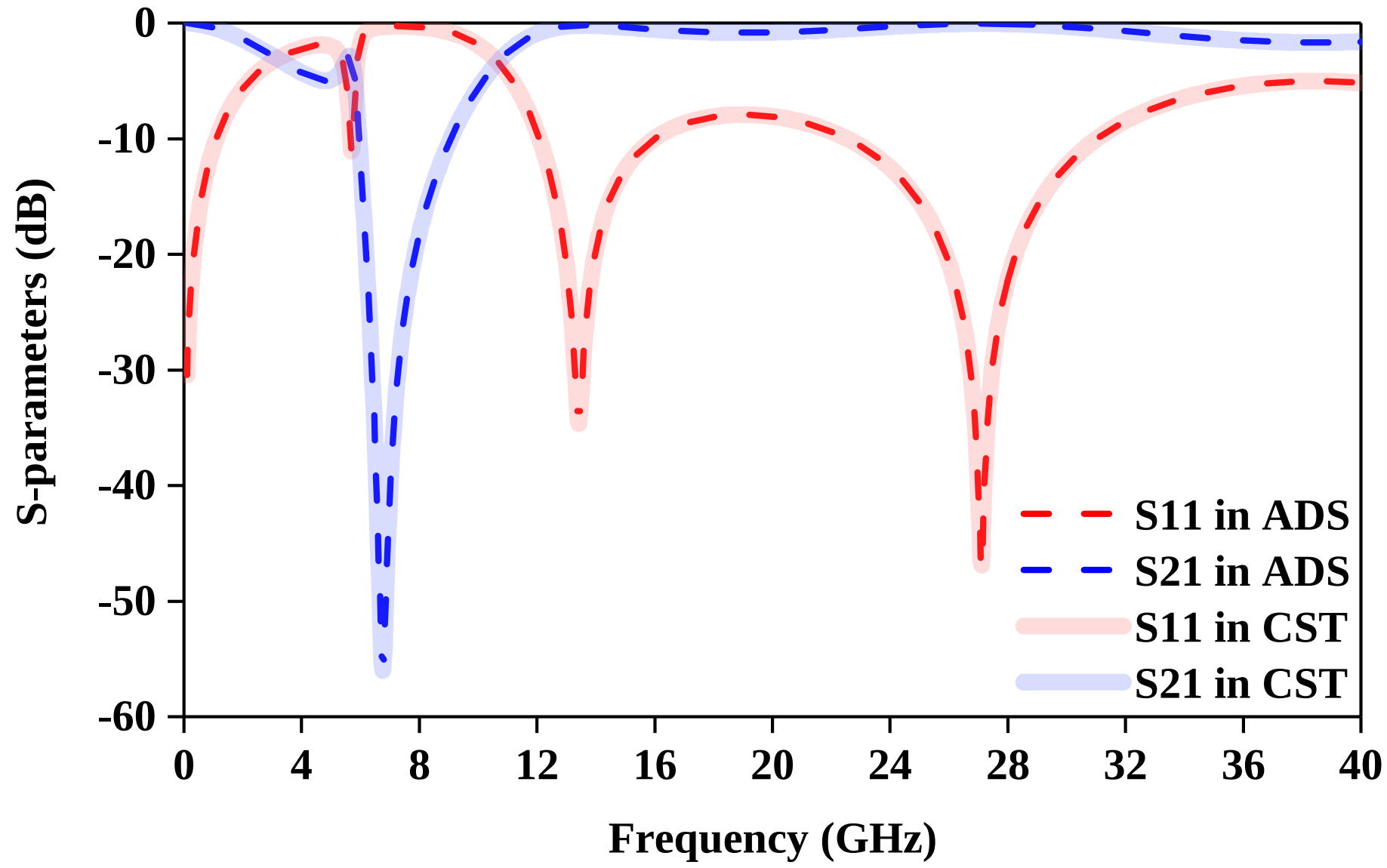

Fig. 1 Comparison of simulation results between ADS field-circuit co-simulation and CST full-wave simulations (Random GDCM code: 0 3.5 2.3 0.2 0 0 0 3.5 2.3 0.2 0 0 0 3.175).

Comment 1.2: *The authors realize various metasurface functions by designing multiple metacircuits; however, it should be emphasized that ADS circuit simulation only models idealized circuit responses. Actual passive components (e.g., capacitors, inductors) typically exhibit strong frequency dependence, and may even show nonlinear or dispersive effects under certain conditions. Therefore, the authors are requested to explicitly clarify whether the real characteristics of components (e.g., frequency responses from datasheets, equivalent circuit models, or S-parameter models) were imported into ADS to ensure that the circuit simulation results reasonably approximate the actual EM behavior of physical devices.*

Response: We thank the reviewer for raising this important concern. Indeed, real passive components do exhibit parasitic effects, frequency dependence, nonlinearity, and dispersive behaviors that idealized circuit models do not capture. We appreciate the opportunity to clarify how our work addresses these practical considerations.

In our design workflow, we use ideal RLC models during the design phase, with real component characteristics fully incorporated during the validation phase after the ideal design is complete. The workflow proceeds as follows. The model first outputs ideal RLC parameter combinations corresponding to a target response. Real components with the closest available values are then selected from manufacturer catalogs. Finally, the

S-parameter models of actual components, which include full frequency dependence and parasitic effects, are imported into the simulation for final validation. This ensures that while our training data generation benefits from the efficiency of ideal models, all final designs are rigorously verified against realistic component behaviors.

We adopt ideal models in the design phase for two key reasons. First, ideal models allow us to explore the broadest possible circuit parameter space without being constrained by specific component libraries. Second, circuit topologies discovered using ideal models are universal and can be mapped to real components from various manufacturers.

We recognize that practical lumped components with packaging introduce parasitic effects and nonlinearities that can affect model accuracy. Therefore, when implementing designs, we select components with minimal parasitic parameters and ensure that the operating frequency remains well below their self-resonant frequencies. This practice helps maintain good agreement between simulation and measurement.

For high-frequency applications, we have proposed a micro-nano integration pathway. Through micro-nano fabrication, RLC networks are fabricated directly within the chip, which significantly reduces parasitic inductance and capacitance introduced by pads, interconnects, and solder joints. Integrated passive devices can achieve self-resonant frequencies exceeding the Ka-band [1-4]. With parasitic effects precisely controlled and minimized, the discrepancy between ideal models and measured responses is significantly reduced.

Following this design philosophy, the fabricated prototypes exhibit excellent agreement with simulations, as demonstrated by the experimental results presented in this work. We have clarified this design validation workflow in the revised manuscript(Page 11, Lines 359-365).

Revisions have been made as follows:

Manuscript:

Methods

Design and validation workflow for component modeling

The trained model first generates ideal RLC parameters for a target response. Real components with the closest available values are then selected based on manufacturer datasheets. Finally, S-parameter models of these components are imported into a full-wave simulator to validate the design under realistic conditions. This three-stage approach ensures that designs benefit from the efficiency of ideal models during training while being rigorously validated against real component behavior....

[1] Zeng, Y., Wu, Y., Yang, Y., Pan, L., Wang, W.: A novel minimized millimeter-wave on-chip spoof surface plasmon polariton and its applications based on IPD technology. Microelectronics Journal **162**, 106755 (2025). https://doi.org/10.1016/j.mejo.2025.106755

[2] Li, Y., Ni, J., Li, S., Han, Q., Tian, J., Li, N., Ma, T.: Design of a millimeter-wave wideband filtering balun in GaAs IPD technology. Frontiers in Physics **13**, 1555410 (2025). https://doi.org/10.3389/fphy.2025.1555410

[3] Ji, N., Shen, G., Feng, W., Xue, Q., Che, W.: Packaged Ka-band IPD bandpass filter using in-phase and out-of-phase mixed couplings. IEEE Transactions on Components, Packaging and Manufacturing Technology **15**(8), 1750–1758 (2025). https://doi.org/10.1109/TCPMT.2025.3581320

[4] Shen, G., Wen, B., He, Z., Ji, N., Feng, W., Che, W.: A 3.5-/28-GHz dual-band IPD bandpass filter using GaAs process for 5G application. IEEE Microwave and Wireless Technology Letters **35**(6), 686–689 (2025). https://doi.org/10.1109/LMWT.2025.3552089

Comment 2: *The title of this paper is "Efficient EM Large Model Framework Based on Metacircuit-Embedded Surface". However, based on the content, the forward model adopts a conventional fully connected neural network (FCNN) with an input dimension of 14, hidden layers (64–256–512–1024), and an output dimension of 800 to predict responses from circuit parameters. For inverse design, the authors mainly rely on traditional optimization algorithms rather than generative models, differentiable inverse frameworks, or more advanced large-model methods with strong generalization capabilities. Although about 6 million data samples were used, which can be considered a large-scale dataset for EMs-driven inverse design, the definition of a "large model" is not solely determined by data volume. The adopted FCNN consists of 1 input layer, 4 hidden layers, and 1 output layer, whose structural complexity and parameter scale are significantly lower than those typically associated with modern "large models" (i.e., deep architectures, huge parameter counts, or pretraining capabilities). In addition, both forward prediction and inverse design rely on conventional methods without demonstrating architectural innovations, pretraining–fine-tuning pipelines, or cross-task generalization expected from large models. In summary, the current work is more appropriately described as "inverse design based on large-scale data" rather than a genuine "EM large model framework". The authors are suggested to revisit the use of the term "Large Model" in the title and throughout the manuscript, or further improve the model architecture, parameter scale, and inverse-design methodology to justify the "large model" claim.*

Response: We sincerely thank the reviewer for this insightful and constructive suggestion regarding the use of the term "large model" in our manuscript. We fully agree that our neural network's parameter count is relatively small compared to modern large language models or vision transformers, which typically range from hundreds of millions to billions of parameters. We appreciate this guidance, which has helped us more accurately position our contribution.

Our main contribution is not the development of a complex neural architecture but rather the establishment of a unified design framework that leverages large scale, high quality data to enable efficient AI assisted electromagnetic design. By generating 6 million training samples within this framework, the proposed MCES framework demonstrates strong accuracy and versatility, providing a scalable foundation for future studies exploring deeper architectures and larger parameter counts.

To avoid any ambiguity, we have revised the title to "Large-Scale Data-Driven Inverse EM Design Based on Metacircuit-Embedded Surface." In addition, we avoid claiming that we have built a "large model" throughout the manuscript.

Comment 3: *In the introduction, the authors state that micro-nano fabrication can minimize parasitic effects, but they do not further explain the underlying mechanism or provide a comparison with traditional fabrication processes in the main text.*

Response: We thank the reviewer for pointing out this lack of clarity. We appreciate the opportunity to provide a more detailed explanation of the micro-nano fabrication process used in our work and its benefits for high-frequency performance.

Micro-nano fabrication minimizes parasitic effects through extreme miniaturization, precise dimensional control, monolithic integration, and advanced materials. First, extreme miniaturization reduces the physical distances between circuit elements, which directly lowers the parasitic inductance and capacitance associated with interconnects. Additionally, precise dimensional control via lithographic techniques minimizes process induced variations, ensuring consistency between fabrication and design. Furthermore, monolithic integration eliminates the need for discrete component packaging, removing parasitic contributions from bond wires, lead frames, and encapsulation materials. Finally, advanced materials such as high-resistivity substrates and thick metal layers further reduce substrate losses and dielectric absorption. These combined factors result in significantly reduced parasitic effects, which improves the accuracy of circuit models and enhances agreement between simulation and measurement, particularly at higher frequencies where parasitics play a dominant role. The self-resonant frequencies of integrated passive devices fabricated using this approach can exceed the Ka-band, making them well suited for high-frequency applications.

To demonstrate these advantages quantitatively, we compare key parameters with conventional PCB processes. In PCB implementations using surface-mount components, each interconnect contributes parasitic inductance and capacitance arising from pads, traces, and packaging. For example, the Murata GQM1875C2ER10BB12 capacitor has an equivalent series inductance of 0.371 nH, while the Skyworks SMP1340-007LF PIN diode introduces approximately 0.4 nH of parasitic inductance from its lead frame and bond wires. Similarly, the Murata LQG15HH1N0B02 inductor shows a parasitic capacitance of approximately 0.1 pF from its internal winding structure. When combined with

additional parasitics from PCB pads and interconnects, the total series inductance typically falls within 0.2–1 nH and the total shunt capacitance within 0.1–0.3 pF. In contrast, micro-nano integration reduces interconnect parasitics to negligible levels. Component self-resonant frequencies increase from around 20 GHz for discrete parts (e.g., the Murata LQP03TG0N1B02) to exceed the Ka-band for integrated passives. Moreover, micro-nano fabrication integrates RLC networks within a footprint below 1 mm^2, rather than the 1–2 mm^2 required by surface-mount processes, achieving a level of circuit complexity and integration density that the latter cannot match. The use of packaged components at high frequencies often introduces significant performance degradation due to these parasitic effects. In contrast, micro-nano integration minimizes such parasitics, ensuring better agreement between simulated and measured responses. In the main manuscript, we have added a brief reference to this discussion in the Methods section (Page 11, Lines 343-349) and Supplementary Information (Page 34, Lines 697-730).

Revisions have been made as follows:

Manuscript:

Methods

Micro-nano fabrication considerations

....Micro-nano fabrication minimizes parasitic effects through extreme miniaturization, precise dimensional control, monolithic integration, and advanced materials. A detailed explanation of these mechanisms, along with quantitative comparisons with traditional PCB processes, is provided in Supplementary Information Section 4.....

Supplementary Information:

4.5 Micro-Nano Fabrication for Parasitic Effect Minimization.

....This supplementary note provides a detailed explanation of how micro-nano fabrication minimizes parasitic effects and offers a quantitative comparison with traditional PCB-based fabrication processes.
Micro-nano fabrication minimizes parasitic effects through extreme miniaturization, precise dimensional control, monolithic integration, and advanced materials. First, extreme miniaturization reduces the physical distances between circuit elements, which directly lowers the parasitic inductance and capacitance associated with interconnects. Additionally, precise dimensional control via lithographic techniques minimizes process induced variations, ensuring consistency between fabrication and design. Furthermore, monolithic integration eliminates the need for discrete component packaging, removing parasitic contributions from bond wires, lead frames, and encapsulation materials. Finally,

advanced materials such as high-resistivity substrates and thick metal layers further reduce substrate losses and dielectric absorption. These combined factors result in significantly reduced parasitic effects, which improves the accuracy of circuit models and enhances agreement between simulation and measurement, particularly at higher frequencies where parasitics play a dominant role. The self-resonant frequencies of integrated passive devices fabricated using this approach can exceed the Ka-band, making them well suited for high-frequency applications.

To demonstrate these advantages quantitatively, we compare key parameters with conventional PCB processes. In PCB implementations using surface-mount components, each interconnect contributes parasitic inductance and capacitance arising from pads, traces, and packaging. For example, the Murata GQM1875C2ER10BB12 capacitor has an equivalent series inductance of 0.371 nH, while the Skyworks SMP1340-007LF PIN diode introduces approximately 0.4 nH of parasitic inductance from its lead frame and bond wires. Similarly, the Murata LQG15HH1N0B02 inductor shows a parasitic capacitance of approximately 0.1 pF from its internal winding structure. When combined with additional parasitics from PCB pads and interconnects, the total series inductance typically falls within 0.2–1 nH and the total shunt capacitance within 0.1–0.3 pF. In contrast, micro-nano integration reduces interconnect parasitics to negligible levels. Component self-resonant frequencies increase from around 20 GHz for discrete parts (e.g., the Murata LQP03TG0N1B02) to exceed the Ka-band for integrated passives. Moreover, micro-nano fabrication integrates RLC networks within a footprint below 1 mm^2, rather than the 1–2 mm^2 required by surface-mount processes, achieving a level of circuit complexity and integration density that the latter cannot match. The use of packaged components at high frequencies often introduces significant performance degradation due to these parasitic effects. In contrast, micro-nano integration minimizes such parasitics, ensuring better agreement between simulated and measured responses....

Comment 4: *The authors state that mapping the high-dimensional discontinuous geometric domain to a low-dimensional continuous circuit-parameter space significantly reduces optimization complexity. However, for conventional geometric designs (not discretization-based topology optimization), geometric parameters themselves are continuous variables. The authors should provide a clear explanation.*

Response: We thank the reviewer for raising this important suggestion. We agree that geometric parameters are continuous under a fixed topology. However, in our paper, "dis-

continuity" specifically refers to the discreteness of topological types rather than parametric variations. To avoid any potential ambiguity, we have revised the corresponding sentence in the main text (Page 2, Lines 84–87).

Revisions have been made as follows:

> **Manuscript:**
>
> Introduction
>
> *....With this approach, various metasurface functions can be attained simply by replacing the metacircuit, without altering the underlying structure. By mapping the design space from a high-dimensional geometric domain where discontinuities arise from switching between distinct topological families to a low-dimensional continuous circuit parameter space within a fixed topology, our method significantly reduces optimization complexity. Moreover, replacing full-wave simulations with fast circuit-based models....*

Comment 5: *The proposed MCES framework can generate millions of data samples within a short time via ADS circuit simulation for designing metasurfaces with diverse performances, thus improving model generalization. This does not mean that conventional EM modeling cannot achieve a similar data scale. In fact, traditional EM modeling can also obtain large-scale datasets with various performance metrics via full-wave simulations of numerous structures. The main difference lies in data acquisition efficiency: the advantage of MCES is that it drastically reduces single-simulation time and thus generates large-scale data more efficiently under the same computational resources, rather than invalidating the ability of traditional EM simulation to produce massive data.*

Response: We thank the reviewer for raising this important point. In fact, traditional EM modeling can also generate large-scale datasets with various performance metrics through full-wave simulations of numerous structures. However, this approach requires access to high-performance computing servers and needs prohibitively long simulation times.

To avoid any potential misunderstanding, we have revised the corresponding sentence in the main text (Page 11, Lines 332-342).

Revisions have been made as follows:

> **Manuscript:**
>
> Methods
>
> *Training data generation*
>
> *Traditional EM modeling can indeed obtain large-scale datasets with various performance metrics via full-wave simulations of numerous structures. However, this approach requires access to high-performance computing servers and*

involves prohibitively long simulation times. Generating millions of samples using conventional methods would take years of computation, making such large-scale data generation practically infeasible for most research groups.

The key advantage of the MCES framework lies not in enabling large-scale data generation, but in achieving this with dramatically improved efficiency. By reducing the simulation time per sample from minutes to milliseconds, MCES can generate millions of samples in days on a consumer-grade laptop. This represents an improvement of approximately two orders of magnitude compared to conventional approaches and democratizes access to large-scale data-driven design for researchers without access to supercomputing resources....

Comment 6: *The paper mentions that metasurfaces with different functions can be realized by modifying the metacircuit design. However, in engineering applications, redesigning and fabricating different circuit structures via micro-nano processes for each function usually leads to much higher fabrication complexity and cost compared with conventional metal-patch-based structures for the same performance. The authors should further clarify the advantages of the proposed method in terms of practical manufacturing cost, scalability, and engineering feasibility.*

Response: We thank the reviewer for raising this important suggestion. We understand that our description of micro-nano fabrication may have led the reader to associate it with high-cost semiconductor processes. We wish to clarify that this is not the case. The micro-nano fabrication used for our metachiplets is based on electron-beam lithography, a direct-write technique that does not require expensive photomasks and offers short fabrication cycles at controllable cost.

To provide a clearer illustration of the cost, we obtained pricing information from commercial foundries. Based on the quotes received, the fabrication cost is approximately 0.23 USD per metachiplet, calculated from 1,390 USD for 6,000 units. For comparison, the total cost of discrete components for a series RLC network is approximately 0.24 USD, including a resistor (RT0201FRE07150RL at 0.023 USD), an inductor (Murata LQW03AW8N2J00D at 0.075 USD), and a capacitor (Murata GQM1875C2ER10BB12 at 0.138 USD). This shows that the metachiplet cost is already slightly lower than the combined discrete component cost, without considering additional assembly expenses.

When comparing micro-nano fabrication with traditional PCB processes, a fair assessment must also consider time cost, not just unit fabrication cost. In the research and development phase, rapid iteration without mask costs offers significant time savings over conventional PCB processes. Traditional PCB approaches require weeks per iteration with new mask fabrication. In contrast, design errors in our workflow can be corrected by simply updating the design file and re-exposing the next day, cutting iteration time from

weeks to days. When amortized across multiple design cycles and production runs, the overall cost becomes increasingly favorable.

At large-scale production, the cost advantage becomes increasingly clear. A single run produces tens of thousands of metachiplets, and the same design can be reused across multiple projects with no additional development cost. Considering the full workflow from design to production, the overall cost is acceptable, especially when performance and design flexibility are priorities. Rapid iteration during development combined with scalable production costs makes the MCES framework a practical solution for real-world engineering applications. We have added this analysis to the revised manuscript (Page 11, Lines 350-353) and Supplementary Information (Page 35, Lines 731-751).

Revisions have been made as follows:

Manuscript:

Methods

Cost Comparison between Micro-Nano Fabrication and Conventional Methods

A detailed cost analysis of the micro-nano fabrication process, including component-level cost breakdown, comparison with discrete alternatives, and full-workflow economic assessment, is provided in Supplementary Information Section 4. ....

Supplementary Information:

4.6 Cost Comparison between Micro-Nano Fabrication and Conventional Methods

To better illustrate the cost, we obtained pricing information from commercial foundries. Based on quotes from commercial foundries, the fabrication cost is approximately 0.23 USD per metachiplet (calculated from 1,390 USD for 6,000 units). For comparison, the total cost of discrete components for a series RLC network is approximately 0.24 USD, including a resistor (RT0201FRE07150RL at 0.023 USD), an inductor (Murata LQW03AW8N2J00D at 0.075 USD), and a capacitor (Murata GQM1875C2E R10BB12 at 0.138 USD). This shows that the metachiplet cost is already slightly lower than the combined discrete component cost, without considering additional assembly expenses.

In the research and development phase, rapid iteration without mask costs offers significant time savings over conventional PCB processes. Traditional PCB approaches require weeks per iteration with new mask fabrication. In contrast, design errors in our workflow can be corrected by simply updating the design file and re-exposing the next day, cutting iteration time from weeks to days. When amortized across multiple design cycles and production runs, the overall cost becomes increasingly favorable.

At large-scale production, the cost advantage becomes increasingly clear. A single run produces tens of thousands of metachiplets, and the same design can be reused across multiple projects with no additional development cost. Considering the full workflow from design to production, the overall cost is acceptable, especially when performance and design flexibility are priorities. Rapid iteration during development combined with scalable production costs makes the MCES framework a practical solution for real-world engineering applications....

Comment 7: *The authors claim that large-scale datasets can reduce the requirements for AI training. This statement needs further clarification. Large datasets do help improve generalization and reduce overfitting, which may relax requirements on model structure or regularization. Nevertheless, large datasets do not reduce the demand for computational resources during training, and excessive low-quality data may even degrade training performance. The authors should use a more accurate expression.*

Response: We sincerely thank the reviewer for this precise and valuable suggestion. We agree that our original statement lacked precision, as a large dataset does not reduce the fundamental requirements of AI training.

To avoid any ambiguity, we have revised the title of the relevant subsection in the Supplementary Information (Page 21, Lines 351-352).

Revisions have been made as follows:

Supplementary Information:

3.3 Large training sample size enhances model accuracy and generalization capability

Comment 8: *Regarding the dataset size: ADS circuit simulation is computationally cheap, and the authors used a million-level dataset for model training, but the justification for this scale is insufficient. Generally, circuit models with clear physical mechanisms do not require such a huge amount of data. Supplementary Fig. S11 shows the MSE versus training epochs and the generalization performance under different dataset sizes, but the authors did not analyze the implications of these results. A systematic sensitivity analysis is suggested based on the provided curves:*

- Does model performance saturate beyond a certain data size?

- Is there obvious redundancy in the million-level data?

- Does the improvement in accuracy/generalization exhibit diminishing returns with increasing data volume?

- Can a minimum acceptable data range be provided?

In addition, the authors should clarify whether the generalization improvement from massive data has practical engineering significance.

Response: We are grateful to the reviewer for raising this important point. Following the reviewer's suggestion, we trained models using sample sizes of 1×10^4, 1×10^5, 1×10^6, 2×10^6, 3×10^6, 4×10^6, 5×10^6, and 6×10^6 and evaluated their performance. All models share the same network architecture, consisting of four hidden layers with 64, 256, 512, and 1024 neurons respectively. All training runs were conducted under identical hardware conditions using a system equipped with an AMD EPYC 7402 CPU at 2.79 GHz. This ensures a fair comparison across different dataset sizes. We first show the relationship between model MSE and dataset size in Table I and Fig. 2. We then demonstrate the predictive capability of models trained on different sample sizes for out-of-sample configurations in Table II and Fig. 3.

Table I: VALIDATION MSE FOR MODELS TRAINED WITH DIFFERENT DATASET SIZES (600 EPOCHS)

**Dataset Size**	**Validation MSE**	**Dataset Size**	**Validation MSE**
1×10^4	3.79×10^{-3}	3×10^6	1.99×10^{-4}
1×10^5	4.39×10^{-4}	4×10^6	1.74×10^{-4}
1×10^6	2.73×10^{-4}	5×10^6	1.66×10^{-4}
2×10^6	2.15×10^{-4}	6×10^6	1.63×10^{-4}

(1) Does model performance saturate beyond a certain data size?

Model performance saturates after 5×10^6 samples. As shown in Fig. 2(b), the model MSE decreases from 3.79×10^{-3} at 1×10^4 samples to 1.63×10^{-4} at 6×10^6 samples. However, the rate of improvement slows significantly after 3×10^6 samples, and the performance essentially saturates around 1.6×10^{-4} once the dataset exceeds 5×10^6 samples.

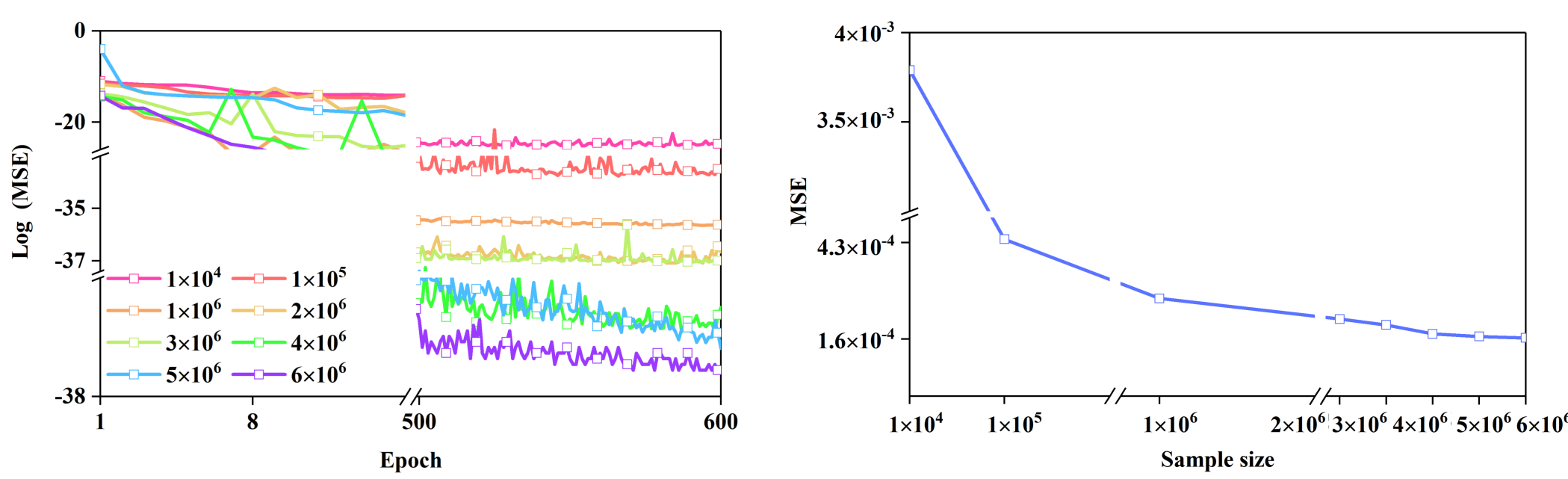

(a) (b)

Fig. 2 Validation MSE versus dataset size. (a) Validation MSE on a logarithmic scale. (b) Validation MSE in linear scale.

Therefore, we consider that the model performance saturates when the dataset size exceeds 5×10^6.

(2) Is there obvious redundancy in the million-level data?

The dataset exhibits some redundancy, yet it is not excessive. First, as shown in Fig. 2(b), the validation MSE continues to decrease as dataset size increases from 1×10^4 to 6×10^6. As the sample size increased from 1×10^6 to 6×10^6, the MSE decreased by 40.3%. Second, and more critically, Table II shows that the out-of-sample prediction error drops from 55.19 to 6.06×10^{-4} when the sample size increases from 1×10^4 to 6×10^6, a reduction exceeding five orders of magnitude. Notably, the error continues to decrease even beyond 1×10^6 samples, dropping from 0.110 to 6.06×10^{-4} when the sample size increases from 1×10^6 to 6×10^6, a 180-fold improvement. If the dataset were dominated by redundant samples, the marginal benefit of additional samples would quickly diminish, and both validation and out-of-sample errors would saturate. The observed continuous improvement confirms the value of the million level dataset. Despite some redundancy, data increments still provide a wealth of new information and effectively cover different design spaces.

Table II: Out-of-sample MSE for models trained with different dataset sizes

**Dataset Size**	**Out-of-sample MSE**	**Dataset Size**	**Out-of-sample MSE**
1×10^4	55.19139	3×10^6	0.04398
1×10^5	26.44115	4×10^6	0.03751
1×10^6	0.11023	5×10^6	0.00109
2×10^6	0.045	6×10^6	6.06007×10^{-4}

(3) Does the improvement exhibit diminishing returns with increasing data volume?

Indeed, diminishing returns are evident. The validation MSE decreases by 92.8% when increasing from 1×10^4 to 1×10^6 samples, but only by an additional 18.2% when increasing from 3×10^6 to 6×10^6 samples. As illustrated in Fig. 2(b), the trend of the model MSE flattens with increasing dataset size, which clearly indicates diminishing marginal returns. Nonetheless, the continued improvement beyond 3×10^6 samples confirms that additional data still contribute meaningful information.

(4) Can a minimum acceptable data range be provided?

At least 5×10^6 samples are sufficient for practical applications. As shown in Fig. 3, the out-of-sample prediction accuracy improves continuously with the sample size. Specifically, Figs. 3(a) and (b) show that when the sample size is 10^4 and 10^5, the predicted values exceed 10, which is unacceptable for practical design applications. As the sample size increases, the accuracy improves. However, as shown in Fig. 3(c) to (f), the predicted values still differ from the simulated results when the sample size is below 4×10^6, with some predicted values exceeding 1. Until the sample size reaches 5×10^6, the prediction

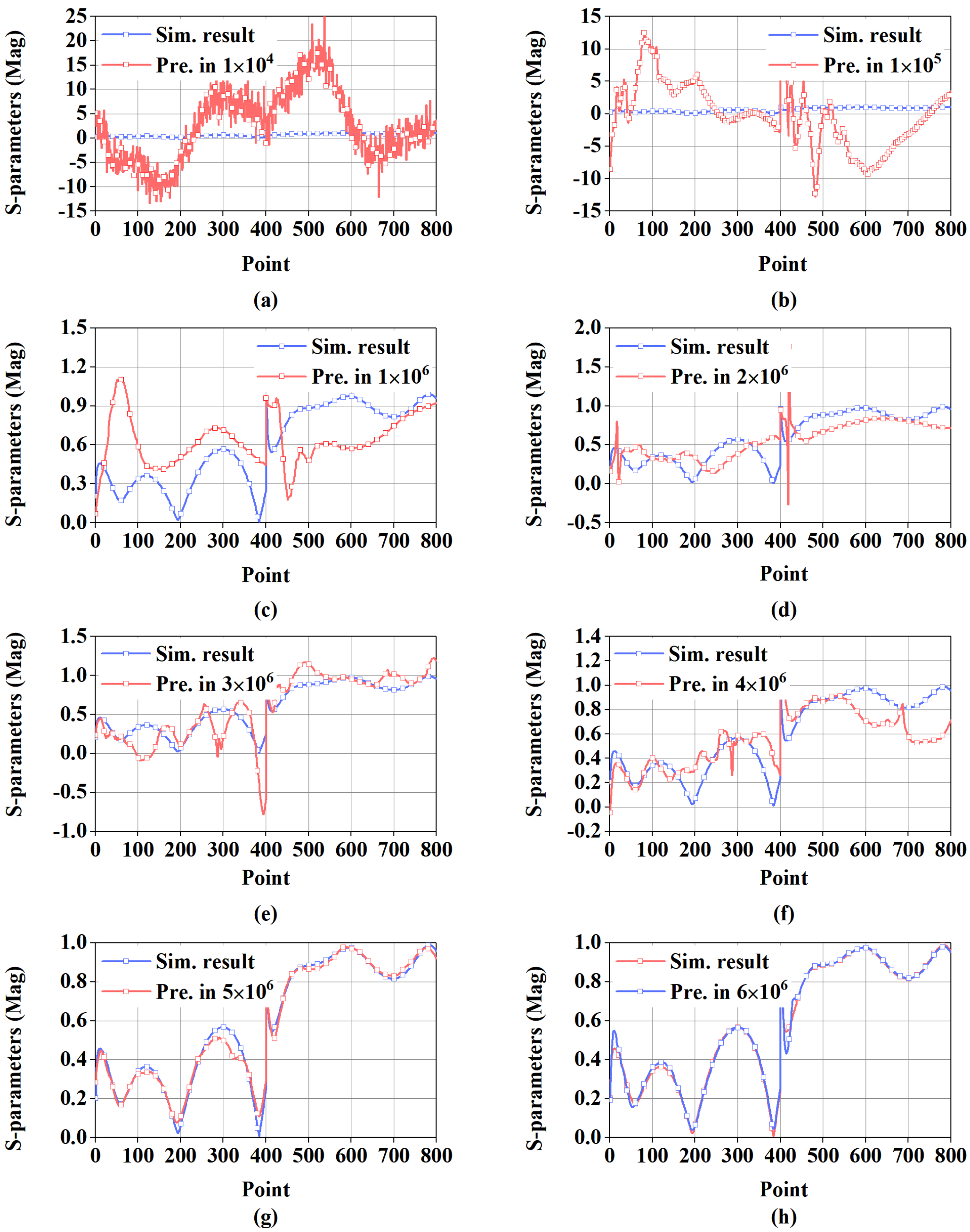

Fig. 3 Comparison of predicted and simulated responses for an out-of-sample configuration (GDCM code: 0 450 21 0.6 450 21 0.6 450 21 0.6 450 21 0.6 4) using models trained with different dataset sizes.

error drops to the level of 10^{-3}. At this point, the predicted values are highly consistent with the simulation results, reaching a practical level of accuracy. The full dataset with 6×10^6 samples further increased the MSE to 6.06×10^{-4}, and the predicted values showed excellent consistency with the simulation results. Therefore, a minimum dataset size of approximately 5×10^6 samples is recommended for practical design applications.

(5) In addition, the authors should clarify whether the generalization improvement from massive data has practical engineering significance.

The generalization improvement from massive data is practically significant. As shown in Fig. 3, the predicted values and simulation results are highly consistent only when the sample size exceeds 5×10^6. At this point, the out-of-sample MSE reaches the 10^{-3} level, achieving the accuracy required for practical applications. Specifically, when the sample size increased from 1×10^6 to 6×10^6, the out-of-sample MSE significantly decreased from 0.110 to 6.06×10^{-4}, representing a 180-fold reduction. Such a reduction in prediction error directly benefits the accuracy of inverse design for out-of-sample designs, a key requirement for practical engineering applications. The ability to achieve prediction errors on the order of 10^{-4} with the full dataset ensures that the trained model can be confidently used for practical metasurface design tasks.

In summary, model performance saturates beyond 5×10^6 samples. The million-level dataset contains some redundancy, but not excessively, as additional samples continue to improve validation and out-of-sample errors. Diminishing returns are clearly observed, as the trend of the model MSE flattens with increasing dataset size. Nonetheless, the continued improvement beyond 3×10^6 samples confirms that additional data still contribute meaningful information. A minimum dataset size of approximately 5×10^6 samples is recommended for practical design applications, as it consistently reduces the out-of-sample prediction error to the 10^{-3} level. We have added this analysis to the Supplementary Information (Page 25, Lines 452-482).

Revisions have been made as follows:

Supplementary Information:

3.6 Dataset Size Justification Analysis

To quantitatively assess the impact of dataset size on model performance, we trained models using sample sizes of 1×10^4, 1×10^5, 1×10^6, 2×10^6, 3×10^6, 4×10^6, 5×10^6, *and* 6×10^6, *as shown in Fig. S13 and Table S10.*
As the dataset size increases from 1×10^4 *to* 1×10^6, *the validation MSE decreases by approximately 92.8%. The rate of improvement slows after* 3×10^6 *samples, with an additional reduction of only 18.2% when increasing from* 3×10^6 *to* 6×10^6 *samples. As illustrated in Fig. S13(b), the trend of the model MSE flattens with increasing dataset size, which clearly indicates diminishing marginal returns.*

To evaluate generalization capability, we selected an out-of-sample circuit configuration not included in any training subset (GDCM code: 0, 450, 21, 0.6, 450, 2, 0.6, 45, 21, 6, 4). The MSE between the model predictions and full-wave simulation results is reported in Table S11 and Fig. S14.
The model trained on 6×10^6 samples achieves an out-of-sample MSE of 6.06×10^{-4}, a reduction of approximately 91,073 times compared to the model trained on 1×10^4 samples. Notably, the out-of-sample MSE reaches the 10^{-3} level at 5×10^6 samples, indicating practically useful accuracy.
Based on the above analysis, we summarize the key observations regarding dataset size effects. Model performance continues to improve up to 6×10^6 samples, with the rate of improvement diminishing after 3×10^6 samples. The validation MSE stabilizes around 1.6×10^{-4} beyond 5×10^6 samples. Some redundancy exists in the dataset, but it is not excessive. The validation MSE continues to decrease from 1×10^6 to 6×10^6 samples (40.3% reduction), and the out-of-sample error drops by a factor of 180 over the same range, confirming that additional samples provide meaningful new information. Diminishing returns are clearly observed: the MSE reduction is 92.8% from 1×10^4 to 1×10^6 samples, but only 18.2% from 3×10^6 to 6×10^6 samples.
Regarding the minimum viable dataset size, a dataset of 1×10^6 samples yields an out-of-sample MSE of 0.110, which is insufficient for practical use. Increasing to 5×10^6 samples brings the error to the 10^{-3} level (0.00109), achieving practically useful accuracy. The full 6×10^6 dataset further improves the error to 6.06×10^{-4}. Thus, a minimum of 5×10^6 samples is recommended for reliable design tasks.
Finally, the generalization improvement has clear practical engineering significance. The 180-fold reduction in out-of-sample error from 1×10^6 to 6×10^6 samples directly translates to more accurate inverse design for unseen configurations. Achieving prediction errors on the order of 10^{-4} with the full dataset ensures that the trained model is suitable for practical metasurface design applications....

Comment 9: *In Fig. S12, the authors present inversely designed metasurface structures validated by full-wave simulation and measurement. However, all training data were obtained from ADS circuit simulations. The authors should further clarify: during inverse design, was the model optimized to match the response from ADS circuit simulation, which was then verified by CST full-wave simulation?*
Response: We thank the reviewer for raising this valuable suggestion. During the inverse design process, we optimize the metacircuits using a model trained on ADS field-circuit co-simulation data to achieve the desired performance. In other words, this can be regarded as matching the response of the ADS field-circuit co-simulation. The optimization

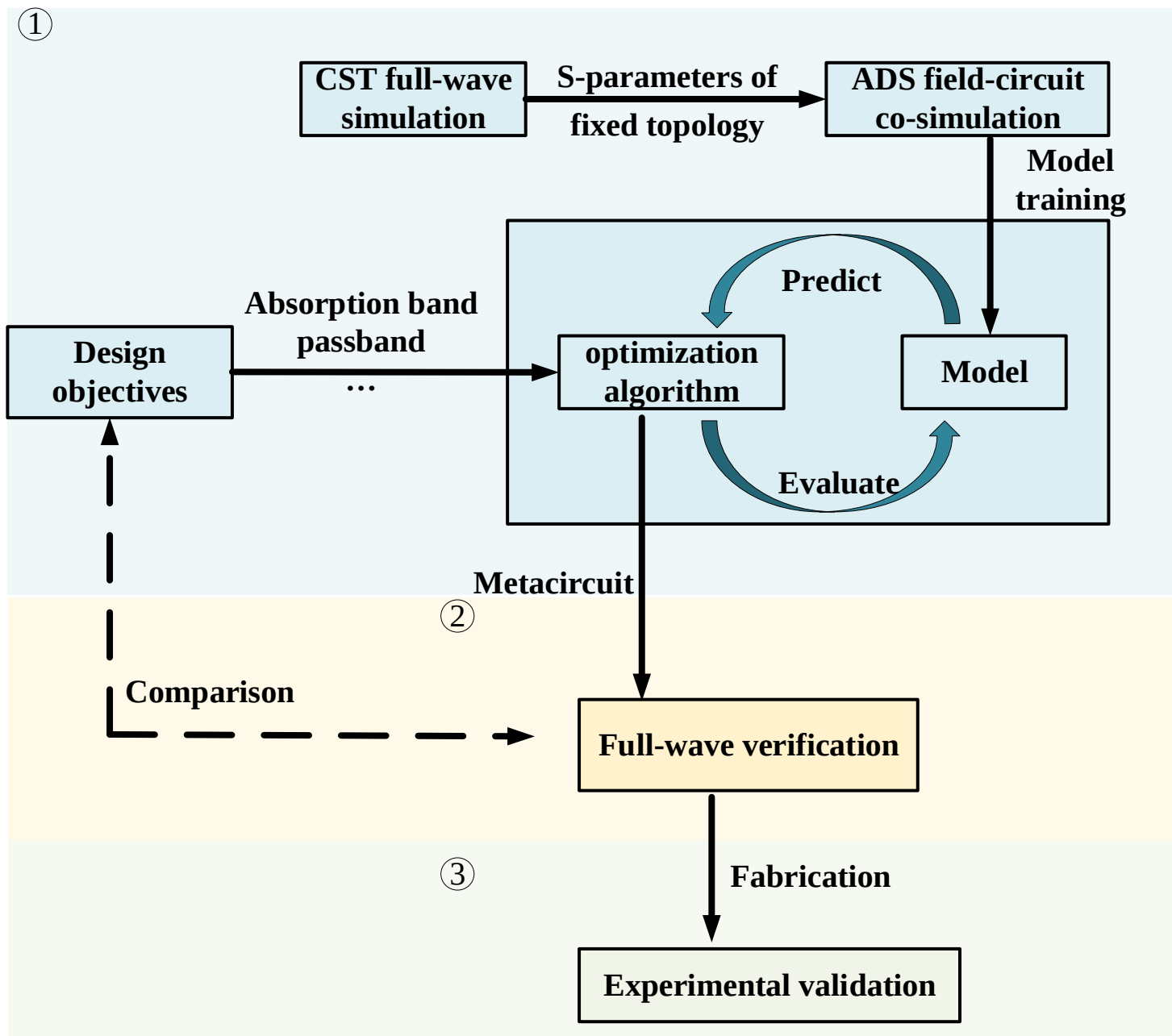

Fig. 4 The three-stage workflow of inverse design.

invokes the trained forward model rather than running ADS simulations directly. Once the model outputs an optimized circuit configuration, the design is directly validated in CST full-wave simulations. The inverse design and validation workflow follows three stages as shown in Fig. 4. In Stage 1, circuit-level optimization runs entirely within the model space. Given a design target, particle swarm optimization searches the circuit parameters using the trained forward network as a fast evaluator. No ADS simulations are run at this stage. In Stage 2, the optimized metacircuit guides the selection of real components from manufacturer catalogs. S-parameter models of real components are then imported into CST for full-wave validation. In Stage 3, selected designs are fabricated and measured to validate real-world performance.

To avoid any ambiguity, a detailed description of this workflow is provided in the Supplementary Information (Page 22, Lines 369-374).

Revisions have been made as follows:

Supplementary Information:

3.4 Leveraging the large-scale datasets generated by MCES, we achieve rapid design of diverse metasurfaces through classical optimization algorithms.

....we employ optimization algorithms to achieve metasurface functionalities

within specified frequency ranges. The inverse design workflow has three stages. First, particle swarm optimization searches the circuit parameter space using the trained forward network as a fast evaluator to find configurations that meet a given design target. No ADS simulations are used at this stage. Second, the optimized circuit parameters guide the selection of real components from manufacturer catalogs. Their S-parameter models are imported into CST for full-wave validation. Third, selected designs are fabricated and measured....

Comment 10: *The authors achieved fast design of diverse metasurfaces via classical optimization algorithms but did not specify the exact algorithm used. From the term "particle swarm size", it can be inferred that particle swarm optimization (PSO) was likely adopted. The authors are suggested to explicitly name the optimization algorithm, briefly introduce its principle, and describe its detailed implementation in the design flow. Furthermore, please explain why PSO was chosen over other popular global optimization methods (e.g., genetic algorithm (GA), differential evolution (DE), simulated annealing (SA)) and provide a comparative analysis. Performance metrics such as convergence speed and solution stability are strongly recommended to verify the advantages and rationality of PSO for this inverse design task.*

Comment 10.1: *The authors achieved fast design of diverse metasurfaces via classical optimization algorithms but did not specify the exact algorithm used. From the term "particle swarm size", it can be inferred that particle swarm optimization (PSO) was likely adopted. The authors are suggested to explicitly name the optimization algorithm, briefly introduce its principle, and describe its detailed implementation in the design flow.*

Response: We thank the reviewer for this valuable suggestion. We use particle swarm optimization (PSO) for the inverse design stage. PSO is a population-based stochastic algorithm that iteratively updates candidate solutions based on individual and collective best positions to efficiently explore the circuit parameter space.

Fig. 5 shows the optimization workflow. Given a design target, particle swarm optimization searches the circuit parameters using the trained forward network as a fast evaluator. The network predicts the response for each candidate, and the objective function measures how well it meets the design specifications. This feedback guides the search until convergence, producing optimized circuit parameters for full-wave validation and experimental testing.

We apply this approach to frequency selective surface design as a case study. The objective function focuses on engineering requirements rather than perfect curve matching. Key PSO parameters include a swarm size of 250 particles and 100 maximum iterations, with frequency ranges defined by passband and transition band indices in the 800-point response. To ensure statistical reliability, we run multiple independent optimizations (typically 3 to 10) and select the best result.

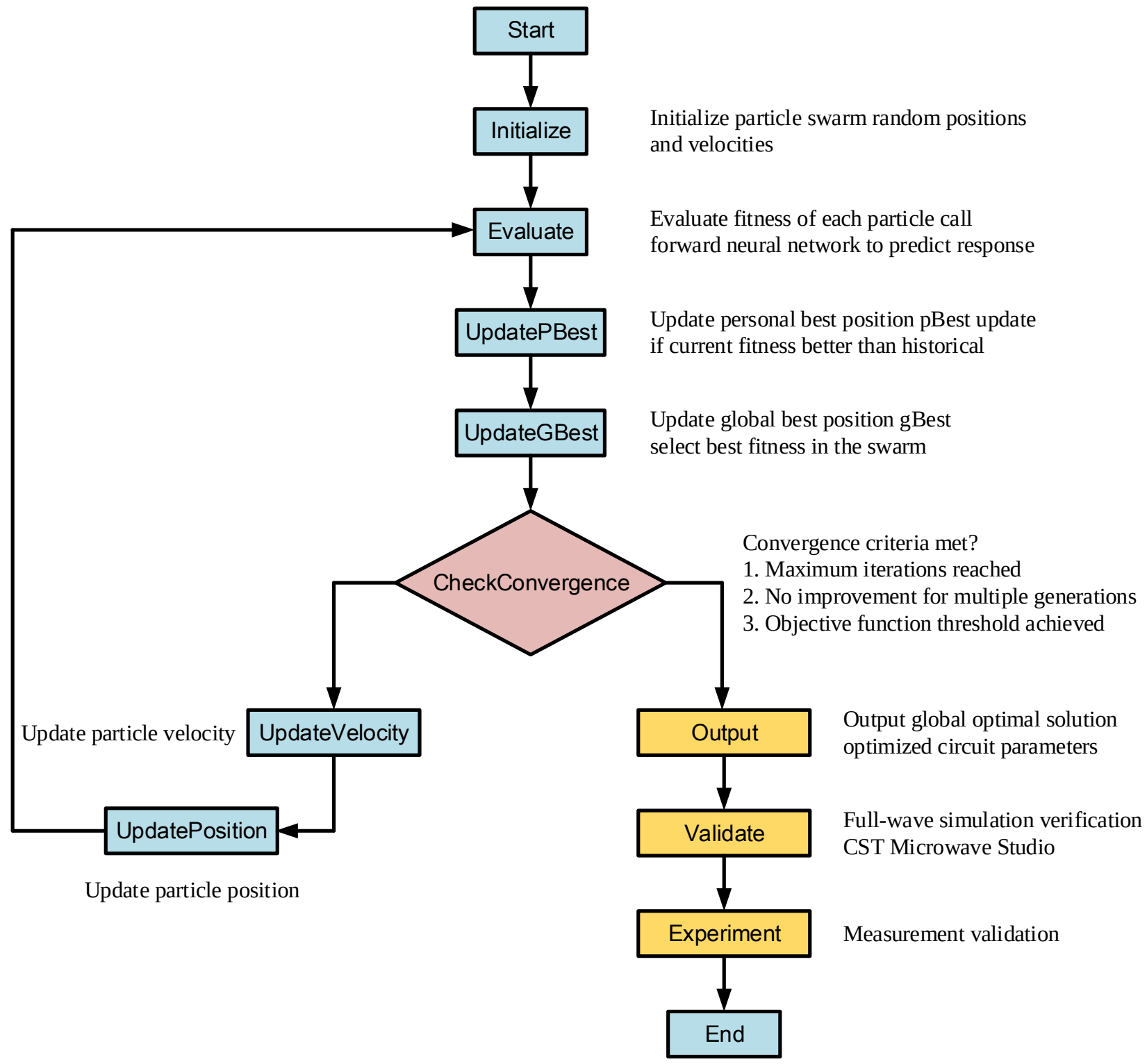

Fig. 5 Workflow of particle swarm optimization for metasurface inverse design.

Comment 10.2: *Furthermore, please explain why PSO was chosen over other popular global optimization methods (e.g., genetic algorithm (GA), differential evolution (DE), simulated annealing (SA)) and provide a comparative analysis. Performance metrics such as convergence speed and solution stability are strongly recommended to verify the advantages and rationality of PSO for this inverse design task.*

Response: We thank the reviewer for this valuable suggestion. Our initial choice of PSO was based on its common usage in EM design, without a rigorous justification. Following the reviewer's suggestion, we have now conducted a systematic comparison of PSO with genetic algorithm (GA), differential evolution (DE), and simulated annealing (SA).

We ran 30 independent optimizations for each algorithm with identical objective functions and termination criteria (50 iterations). Table III summarizes the results. All four algorithms achieved 100% success rates, consistently meeting the design specifications. Simulated annealing gave the best mean objective value (-2.00218) with the smallest standard deviation (0.000159). PSO ranked second (-2.00182), closely followed by GA (-2.00187). DE achieved the lowest mean objective (-2.00102) among the four algorithms.

Table III: COMPARATIVE PERFORMANCE OF GLOBAL OPTIMIZATION ALGORITHMS FOR METASURFACE INVERSE DESIGN (30 INDEPENDENT RUNS)

Algorithm	Best Objective	Mean Objective	Standard Deviation	Average Time (seconds)
PSO (this work)	-2.00266	-2.00182	0.000439	1.55
Genetic Algorithm	-2.00230	-2.00187	0.000335	2.75
Differential Evolution	-2.00166	-2.00102	0.000321	0.47
Simulated Annealing	-2.00252	-2.00218	0.000159	9.22

In terms of speed, DE was the fastest at 0.47 seconds per run, followed by PSO at 1.55 seconds, GA at 2.75 seconds, and SA at 9.22 seconds. GA achieves similar solution quality but requires nearly twice the computation time of PSO. DE converges fastest but yields the poorest solution quality among all algorithms tested. PSO offers the best balance between solution quality and computational efficiency, delivering results comparable to SA (-2.00182 vs -2.00218) while being approximately six times faster (1.55 seconds vs 9.22 seconds).

Most importantly, all four algorithms consistently found valid designs. This confirms that the MCES framework creates a well-conditioned design space where even simple optimization methods work reliably. The framework's strength lies in its data generation capability and unified design paradigm, not in algorithmic complexity.

Following the reviewer's suggestion, we have added details of the PSO implementation in the methods section (Page 11, Lines 354-358) and a full comparative analysis in the Supplementary Information (Page 24, Lines 422-451).

Revisions have been made as follows:

Manuscript:

Methods

Inverse Design Optimization

We have conducted a systematic comparison of particle swarm optimization (PSO) with genetic algorithm (GA), differential evolution (DE), and simulated annealing (SA). All typical algorithms are suitable for our task. Here we choose the PSO algorithm. See Supplementary Information Section 3 for implementation details, algorithm selection, and comparison with other methods.

Supplementary Information:

3.5 Optimization Algorithm for Inverse Design

This supplementary note provides detailed information about the optimization algorithm used for inverse design in the MCES framework, including implementation parameters, algorithm selection rationale, and comparative performance analysis.

Particle swarm optimization (PSO) is a population-based stochastic optimization algorithm inspired by the social behavior of bird flocks and fish schools. In PSO, a swarm of particles explores the search space, where each particle represents a candidate solution in the continuous parameter space defined by the MCES framework (including diode states, R/L/C values, and substrate thickness). For the metasurface inverse design problems in this work, the PSO algorithm was configured with a swarm size of 250 particles and a maximum of 100 iterations, determined empirically to balance exploration and convergence. Other optimization algorithms are also compatible with our method. To demonstrate this, we performed a systematic comparative analysis of PSO against three other global optimization algorithms: genetic algorithm (GA), differential evolution (DE), and simulated annealing (SA). The comparison was conducted on 30 independent runs for each algorithm, using identical objective functions and termination criteria. The results are summarized in Table S9.

All four algorithms achieved 100% success rates, consistently meeting the design specifications. Simulated annealing gave the best mean objective value (-2.00218) with the smallest standard deviation (0.000159). PSO ranked second (-2.00182), closely followed by GA (-2.00187). DE achieved the lowest mean objective (-2.00102) among the four algorithms.

In terms of speed, DE was the fastest at 0.47 seconds per run, followed by PSO at 1.55 seconds, GA at 2.75 seconds, and SA at 9.22 seconds. GA achieves similar solution quality but requires nearly twice the computation time of PSO. DE converges fastest but yields the poorest solution quality among all algorithms tested. PSO offers the best balance between solution quality and computational efficiency, delivering results comparable to SA (-2.00182 vs -2.00218) while being approximately six times faster (1.55 seconds vs 9.22 seconds).

Most importantly, all four algorithms consistently found valid designs. This confirms that the MCES framework creates a well-conditioned design space where even simple optimization methods work reliably. The framework's strength lies in its data generation capability and unified design paradigm, not in algorithmic complexity.

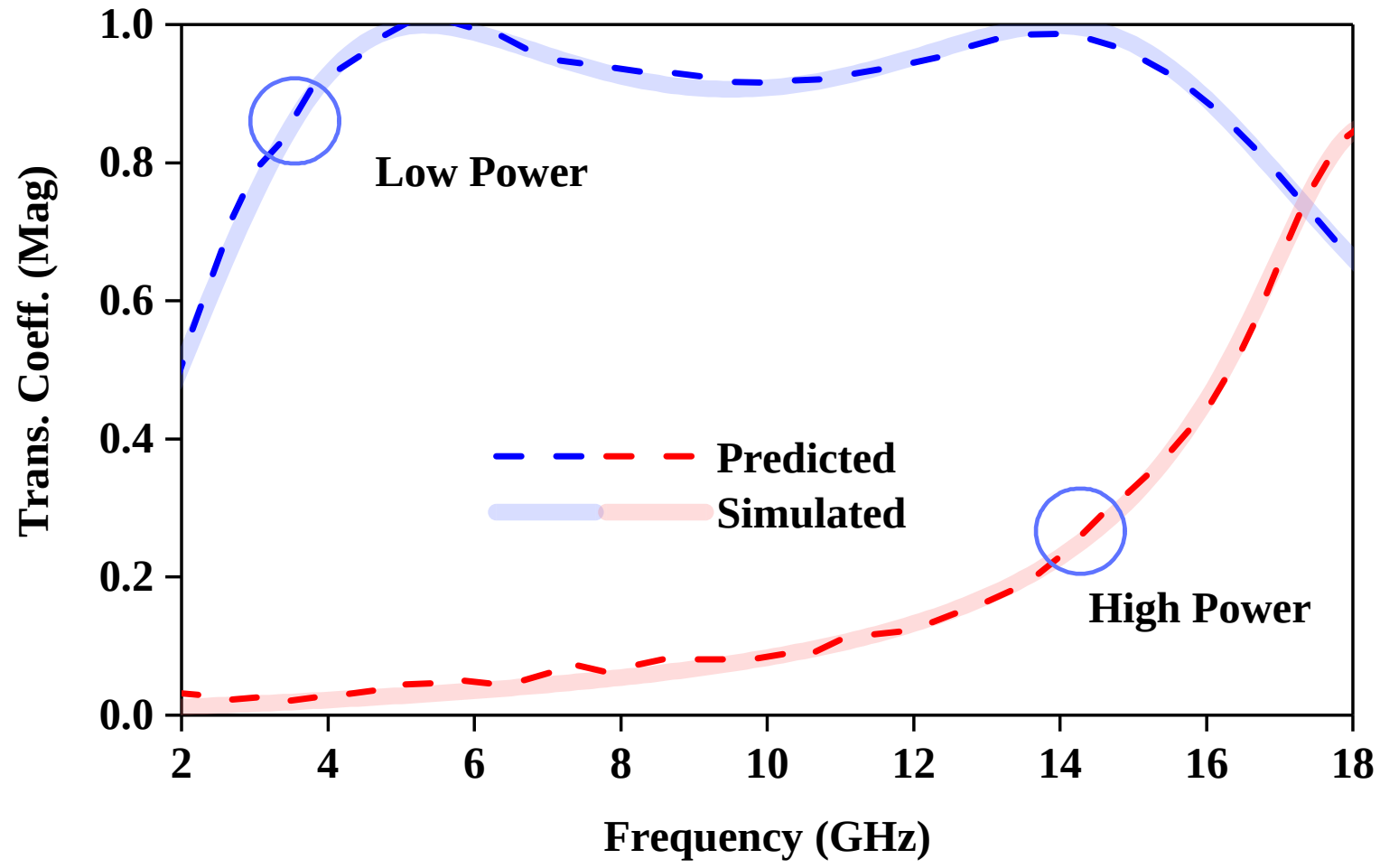

Fig. 6 Comparison of predicted and simulated S-parameters for the active metasurface (ESS) after dataset rebalancing.

Comment 11: *The authors attribute the frequency shift between ESS prediction and simulation to the use of a wider frequency band. The Interpolating Solver in HFSS sets meshes according to the highest simulated frequency; thus, a large difference in the upper frequency can lead to different meshes and discrepancies in EM responses. However, ADS circuit simulation is based on circuit equations (mainly dependent on RLC parameters), where the difference between narrowband and broadband simulation is often negligible. The authors should provide validation or further discussion on this point.*

Response: We appreciate the reviewer's valuable suggestion. It was inaccurate to attribute the discrepancy to the use of a wider frequency band in our initial statement in the Supplementary Information. We have removed this explanation from the revised manuscript. Upon deeper reflection, the observed frequency shift did not stem from bandwidth effects but rather from an imbalance in the proportion of active and passive metasurface samples in the training dataset. We examined the composition of the training data and found that active metasurface samples accounted for only 40% of the total. To address this, we augmented the dataset with additional active metasurface samples to achieve a balanced distribution of 50% active and 50% passive samples. We then retrained the model and recomputed the predictions for the active metasurface (ESS) shown in Fig. S12. Notably, the inverse-designed case for the ESS, shown in Fig. S12, was not included in the training dataset. As illustrated in Fig. 6, the predicted and simulated results are now in good agreement, with a mean squared error of 2.5×10^{-4}. Furthermore, no frequency shift was observed for the ESS in the low power conditions, confirming our conclusion.

Comment 12: *I have some minor issues listed below:*
a) Please verify the DOIs in the references. Certain links, such as references [30] and [31], are currently broken.
b) It is recommended to add scale bars to prototype images (e.g., Fig. 4) to clearly indicate physical dimensions.
Response: We thank the reviewer for the careful reading and for pointing out these minor issues. We have rechecked all DOIs and reference links. Specifically, references [30] and [31] are accessible via their DOIs. In addition, to avoid any potential access issues, all DOIs and links have been verified and confirmed functional. If the access issue still persists, we can provide reference files as supplementary attachments. We have also added clearly labeled scale bars to all prototype images, including Fig. 4, to show the actual device dimensions. We appreciate the reviewer's attention to these details, which has improved the clarity of the manuscript.

Revisions have been made as follows:

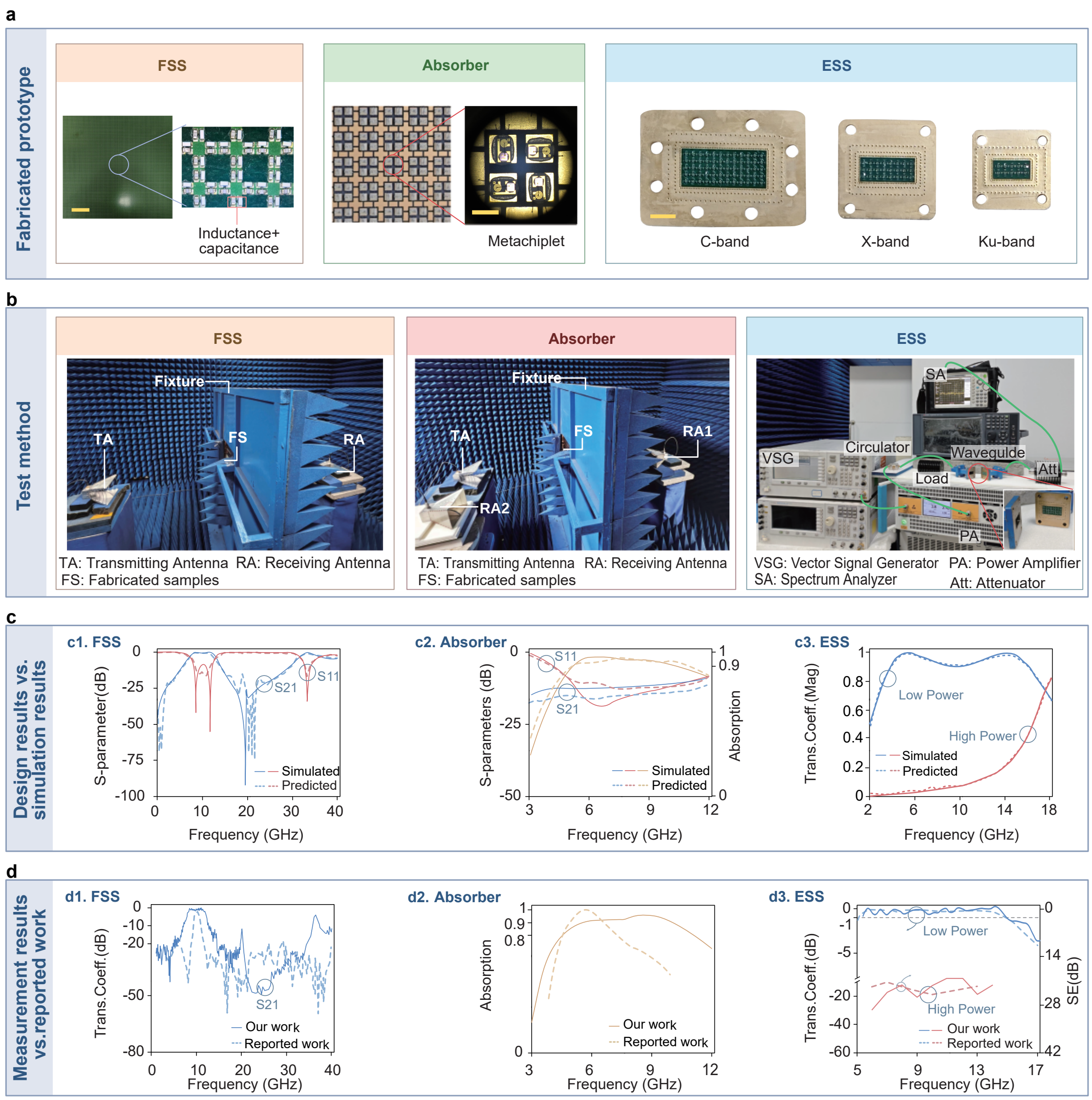

Fig. 4 EM metasurface design and measurement results. **a**, fabricated prototypes of the designed FSS, absorber, and ESS.... Scale bars, 5 cm (FSS), 1 mm (absorber), 1 cm (ESS).

Comment 13: *There are some typo error remains in the manuscript. such as in line 110, the title words "framework" is not correct.*
Response: We thank the reviewer for pointing out these typographical errors. The manuscript has been carefully checked and all errors have been corrected, including the misspelling of "framework" in line 110.

Nature Communications
Original Manuscript ID NCOMMS-26-005356-T
Authors' Response to Reviewer 2

We would like to thank the reviewer for the valuable and constructive comments. We have revised the paper in line with the reviewer's comments, thereby improving the contributions and the clarity of the paper accordingly.

Comment 1: *The manuscript is clearly written, and the presented results are of strong interest to the metasurface design community. The proposed framework represents an important step toward scalable and large-scale metasurface modeling.*
However, the core methodology relies on metacircuits composed of lumped elements to achieve the desired electromagnetic functionality. This raises a fundamental concern regarding high-frequency operation. In practice, lumped resistors, inductors, and capacitors exhibit significant non-idealities at microwave and higher frequencies, including parasitic inductance and capacitance, skin-effect losses, dielectric losses, and limited self-resonant frequencies. These effects can substantially degrade performance, especially when operating near or beyond the self-resonant frequency of the components.
Response: We thank the reviewer for raising this important concern about high-frequency operation. We fully acknowledge that lumped elements exhibit significant non-idealities at microwave and higher frequencies, including parasitic effects, frequency-dependent losses, and limited self-resonant frequencies.

To address these challenges, we adopt a two-fold strategy. First, in our lumped-element implementation, we carefully select high-quality components with self-resonant frequencies above our operating band. For example, Murata's LQP01HV1N1B02 inductor features a self-resonant frequency exceeding 17 GHz, ensuring reliable performance within our frequency range of interest. This component selection, combined with operating frequencies kept safely below device resonances, helps maintain good agreement between simulation and measurement.

Second, for applications requiring even higher frequencies, we have developed a micro-nano integration pathway. This approach minimizes packaging parasitics through several complementary mechanisms. Extreme miniaturization reduces the physical distances between circuit elements, directly lowering interconnect inductance and capacitance. Precise dimensional control via lithographic techniques minimizes process variations, ensuring consistent performance across large arrays. Monolithic integration completely eliminates the parasitic effects caused by discrete component packaging. Advanced materials such as high-resistivity substrates and thick metal layers further reduce substrate losses and dielectric absorption. As a result, integrated passive devices fabricated using this approach can achieve self-resonant frequencies exceeding the Ka-band. These combined strategies ensure that our MCES framework remains effective across a wide frequency range.

We also compare the cost between the lumped-element approach and the micro-nano integration approach. For a series RLC network, the total cost of discrete components is approximately 0.24 USD, consisting of a resistor (RT0201FRE07150RL, 0.023 USD), an inductor (Murata LQW03AW8N2J00D, 0.075 USD), and a capacitor (Murata GQM1875C2ER10BB12, 0.138 USD). In contrast, the fabrication cost per metachiplet using the micro-nano integration approach is approximately 0.23 USD, based on quotes from commercial foundries (1,390 USD for 6,000 units). These results indicate that the micro-nano integration approach achieves comparable cost while offering the advantages inherent to micro-nano fabrication.

We have added a comprehensive discussion of these limitations in the revised manuscript (Page 11, Lines 343-349) and Supplementary Information (Page 33, Lines 652-657, Page 34, Lines 697-713, and Page 35, Lines 731-751).

Revisions have been made as follows:

Supplementary Information:

4.4 MCES enables standardized design of metasurfaces via micro-nano fabrication processes.

....lumped components were employed during fabrication due to the simplicity of the integrated circuits. It is important to note that lumped elements exhibit significant non-idealities at microwave and higher frequencies, including parasitic effects, frequency dependent losses, and limited self-resonant frequencies. Therefore, we carefully select high quality components whose self-resonant frequencies exceed the operating band. This component selection, combined with operating frequencies kept safely below device resonance, helps maintain good agreement between simulation and measurement. However, this fabrication approach struggles to accommodate more complex circuit designs....

4.5 Micro-Nano Fabrication for Parasitic Effect Minimization.

....This supplementary note provides a detailed explanation of how micro-nano fabrication minimizes parasitic effects and offers a quantitative comparison with traditional PCB-based fabrication processes.
Micro-nano fabrication minimizes parasitic effects through extreme miniaturization, precise dimensional control, monolithic integration, and advanced materials. First, extreme miniaturization reduces the physical distances between circuit elements, which directly lowers the parasitic inductance and capacitance associated with interconnects. Additionally, precise dimensional control via lithographic techniques minimizes process induced variations, ensuring consistency between fabrication and design. Furthermore, monolithic integration

eliminates the need for discrete component packaging, removing parasitic contributions from bond wires, lead frames, and encapsulation materials. Finally, advanced materials such as high-resistivity substrates and thick metal layers further reduce substrate losses and dielectric absorption. These combined factors result in significantly reduced parasitic effects, which improves the accuracy of circuit models and enhances agreement between simulation and measurement, particularly at higher frequencies where parasitics play a dominant role. The self-resonant frequencies of integrated passive devices fabricated using this approach can exceed the Ka-band, making them well suited for high-frequency applications.....

4.6 Cost Comparison between Micro-Nano Fabrication and Conventional Methods

To better illustrate the cost, we obtained pricing information from commercial foundries. Based on quotes from commercial foundries, the fabrication cost is approximately 0.23 USD per metachiplet (calculated from 1,390 USD for 6,000 units). For comparison, the total cost of discrete components for a series RLC network is approximately 0.24 USD, including a resistor (RT0201FRE07150RL at 0.023 USD), an inductor (Murata LQW03AW8N2J00D at 0.075 USD), and a capacitor (Murata GQM1875C2ER10BB12 at 0.138 USD). This shows that the metachiplet cost is already slightly lower than the combined discrete component cost, without considering additional assembly expenses.

In the research and development phase, rapid iteration without mask costs offers significant time savings over conventional PCB processes. Traditional PCB approaches require weeks per iteration with new mask fabrication. In contrast, design errors in our workflow can be corrected by simply updating the design file and re-exposing the next day, cutting iteration time from weeks to days. When amortized across multiple design cycles and production runs, the overall cost becomes increasingly favorable.

At large-scale production, the cost advantage becomes increasingly clear. A single run produces tens of thousands of metachiplets, and the same design can be reused across multiple projects with no additional development cost. Considering the full workflow from design to production, the overall cost is acceptable, especially when performance and design flexibility are priorities. Rapid iteration during development combined with scalable production costs makes the MCES framework a practical solution for real-world engineering applications....

Manuscript:

Methods

Micro-nano fabrication considerations

....Micro-nano fabrication minimizes parasitic effects through extreme miniaturization, precise dimensional control, monolithic integration, and advanced materials. A detailed explanation of these mechanisms, along with quantitative comparisons with traditional PCB processes, is provided in Supplementary Information Section 4.....

Comment 2: *The reported agreement between simulation and measurement at high frequencies appears somewhat optimistic in this context. Since the experimental prototypes are small and waveguide-integrated, they contain only a limited number of meta-unit cells and are subject to controlled boundary conditions. This configuration may not fully capture the cumulative loss mechanisms, parasitic coupling, and fabrication tolerances that would arise in a larger, free-space metasurface implementation. It would therefore strengthen the manuscript to clarify how the proposed approach scales to electrically large apertures and whether distributed effects or increased loss would compromise performance.*

Response: We thank the reviewer for this suggestion. We agree that in experimental validation, it is important to consider larger apertures to better reflect practical applications.

Each sample was tested according to the standard method in its field. For the FSS and absorber, we fabricated and tested electrically large prototypes measuring 300 mm $\times$ 300 mm, following the standard testing methods commonly used for such devices [5-8]. These samples are relatively large in size and demonstrate the scalability to electrically large apertures.

The ESS sample size is relatively small. This is due to the requirements of high-intensity radiated field (HIRF) testing. Specifically, the waveguide structure confines electromagnetic energy within a closed environment, enabling safe and controlled high-power experiments without radiation or interference concerns for personnel and the surrounding environment. This is why waveguide-based measurements are the established method for ESS characterization [9–12]. In summary, the fabricated samples are primarily electrically large apertures, demonstrating a certain degree of scalability.

Following the reviewer's suggestion, we have added the following clarification in the Supplementary Information (Page 26, Lines 483-487).

Revisions have been made as follows:

Supplementary Information:

Section 4: Experimental verification of MCES:

To validate the functionality of metasurfaces designed using the MCES framework, we fabricated and tested FSS, ESS, and absorber prototypes. The experimental validation in this study employs two different test methods, each following standard measurement methods in their respective fields....

[5] Li, Z., Weng, X., Li, Y., Yi, X., Liao, M., Bi, M.: Reconfigurable active FSS with switchable passband/stopband response. IEEE Transactions on Antennas and Propagation **74**(3), 2566–2577 (2026). https://doi.org/10.1109/TAP.2025.3650638

[6] Liu, N., Sheng, X., Zhang, C., Guo, D.: Design and synthesis of band-pass frequency selective surface with wideband rejection and fast roll-off characteristics for radome applications. IEEE Transactions on Antennas and Propagation **68**(4), 2975–2983 (2020). https://doi.org/10.1109/TAP.2019.2955665

[7] Han, Y., Liang, M., Ji, Q., Li, Q., Che, W.: Low-profile and wideband paper-based honeycomb microwave absorber with capacitive loops. IEEE Transactions on Antennas and Propagation **73**(6), 3757–3766 (2025). https://doi.org/10.1109/TAP.2025.3542585

[8] Islam, K.M.R., Rahimian, A., Machado, G.G., Abbasi, M.A.B., Cheema, A.A., Meenan, B.J.: Design and experimental performance evaluation of a single-layer polarization-insensitive asymmetric microwave metasurface absorber. IEEE Transactions on Antennas and Propagation **72**(8), 6520–6529 (2024). https://doi.org/10.1109/TAP.2024.3424952

[9] Ning, Z., Li, M., Chen, L., Gu, J., Ding, D.: Circuit modeling and analysis of multiband energy-selective surfaces using characteristic modes. IEEE Transactions on Antennas and Propagation **73**(10), 7583–7595 (2025). https://doi.org/10.1109/TAP.2025.3575579

[10] Jiang, Y., Tian, L., Jiang, Z.H., Hong, W.: Analysis and design of ultra-thin filtering energy-selective surfaces with ultrawideband and high shielding effectiveness. IEEE Transactions on Antennas and Propagation **73**(1), 641–646 (2025). https://doi.org/10.1109/TAP.2024.3464876

[11] Zhou, L., Liu, L., Shen, Z.: High-performance energy selective surface based on the double-resonance concept. IEEE Transactions on Antennas and Propagation **69**(11), 7658–7666 (2021). https://doi.org/10.1109/TAP.2021.3075548

[12] Qu, M., He, Z., Su, J., Nayyeri, V.: A rasorber with energy-selective passband and ultrawideband absorptive out-of-band. IEEE Transactions on Microwaves Theory and Techniques **72**(10), 5643–5652 (2024). https://doi.org/10.1109/TMTT.2024.3387917

Comment 3: *Additionally, some of the optimized lumped element values appear relatively high (e.g., 150 Ω resistors). Realizing such values in micro-/nano-fabrication platforms—particularly in thin-film or integrated implementations—can be challenging due*

to sheet resistance limitations, area constraints, and process variability. The manuscript would benefit from a discussion of fabrication feasibility and tolerances, especially if the long-term goal is integration beyond standard PCB processes.

Response: We thank the reviewer for this suggestion. According to the information from domestic foundries, mature thin-film processes can realize 150 Ω resistors using common materials such as nichrome (NiCr) and tantalum nitride (TaN), which offer typical sheet resistances of 50 Ω/□ and 100 Ω/□, respectively. With a sheet resistance of 50 Ω/□, a 150 Ω resistor can be realized with a minimum size of 0.05 mm × 0.15 mm, while the maximum resistance achievable for a single resistor is 500 Ω with dimensions of 0.05 mm × 0.5 mm. Larger resistance values can be achieved by connecting multiple resistors in series. These results indicate that the 150 Ω value used in our design is feasible within current fabrication capabilities. Nevertheless, higher resistance values involve a trade-off between area and resistance, a practical consideration that future users of the MCES framework should take into account.

Following the reviewer's suggestion, we have added the following clarification in the Supplementary Information (Page 34, Lines 675-681).

Revisions have been made as follows:

> **Supplementary Information:**
>
> *4.4 MCES enables standardized design of metasurfaces via micro-nano fabrication processes.*
>
> *....This approach eliminates the need for endlessly modifying intricate metasurface structures—future researchers need only redesign the integrated circuits within the chip to realize diverse metasurface functionalities. It is important to note that the implementation of metachiplets must consider process limitations. For example, resistance value and area are interrelated. Nichrome (NiCr) and tantalum nitride (TaN), with sheet resistances of 50 and 100 Ω/□, are commonly used to produce resistors. The maximum resistance for a single resistor is 500 Ω with dimensions of 0.05 mm by 0.5 mm. Larger resistance values can be achieved by connecting multiple resistors in series. Therefore, during the design process, we typically require resistance values to be below 500 Ω.....*

Comment 4: *Fully PCB-based metasurfaces are attractive precisely because they are comparatively low-loss, scalable, and fabrication-friendly. Introducing lumped elements may undermine these advantages unless the associated loss mechanisms and practical implementation constraints are carefully addressed.*

A more detailed analysis of component non-idealities, Q-factors, parasitics, and scalability

would significantly strengthen the technical credibility of the proposed approach.
Response: We thank the reviewer for this suggestion. Actually, component non-idealities, including nonlinearity and parasitic effects, cannot be ignored in practical implementations. These factors are indeed critical for maintaining the low-loss and scalable advantages of PCB-based metasurfaces when lumped elements are introduced.

To account for component non-idealities, we select components from manufacturer catalogs that offer high Q-factors and self-resonant frequencies well above our operating band. The actual S-parameter models of these selected components, which include their full frequency-dependent behavior, parasitic effects, and nonlinear characteristics, are then imported into CST full-wave simulations for verification, ensuring that all non-idealities are incorporated into the final designs. The designs are fabricated and measured only after this validation step. Additionally, large-value inductors have lower Q-factors, so we limit the inductance range during optimization.

To further reduce nonlinearity and parasitic effects while supporting high-frequency operation, we also proposed the micro-nano fabrication approach. This method employs direct-write processing, which offers fast iteration, good consistency, and batch production. Flip-chip bonding can also be used to further reduce parasitic effects in future implementations.

This approach directly addresses the reviewer's concerns. High-quality components and standard PCB processes allow designs to be scaled directly to larger apertures. The micro-nano approach further enhances these benefits by supporting high-frequency operation, fast iteration, batch production, and scalability.

Following the reviewer's suggestion, we have added the following clarification in the manuscript (Page 11, Lines 343-349, and Page 11, Lines 359-365) and Supplementary Information (Page 34, Lines 697-730).

Manuscript:

Methods

Design and validation workflow for component modeling

....Real components with the closest available values are then selected based on manufacturer datasheets. Finally, S-parameter models of these components are imported into a full-wave simulator to validate the design under realistic conditions....

Micro-nano fabrication considerations

....Micro-nano fabrication minimizes parasitic effects through extreme miniaturization, precise dimensional control, monolithic integration, and advanced materials. A detailed analysis of these mechanisms, along with quantitative comparisons with conventional PCB processes, is provided in Supplementary Information Section 4....

Supplementary Information:

4.5 Micro-Nano Fabrication for Parasitic Effect Minimization.

....This supplementary note provides a detailed explanation of how micro-nano fabrication minimizes parasitic effects and offers a quantitative comparison with traditional PCB-based fabrication processes.

Micro-nano fabrication minimizes parasitic effects through extreme miniaturization, precise dimensional control, monolithic integration, and advanced materials. First, extreme miniaturization reduces the physical distances between circuit elements, which directly lowers the parasitic inductance and capacitance associated with interconnects. Additionally, precise dimensional control via lithographic techniques minimizes process induced variations, ensuring consistency between fabrication and design. Furthermore, monolithic integration eliminates the need for discrete component packaging, removing parasitic contributions from bond wires, lead frames, and encapsulation materials. Finally, advanced materials such as high-resistivity substrates and thick metal layers further reduce substrate losses and dielectric absorption. These combined factors result in significantly reduced parasitic effects, which improves the accuracy of circuit models and enhances agreement between simulation and measurement, particularly at higher frequencies where parasitics play a dominant role. The self-resonant frequencies of integrated passive devices fabricated using this approach can exceed the Ka-band, making them well suited for high-frequency applications.

To demonstrate these advantages quantitatively, we compare key parameters with conventional PCB processes. In PCB implementations using surface-mount components, each interconnect contributes parasitic inductance and capacitance arising from pads, traces, and packaging. For example, the Murata GQM1875C2ER10BB12 capacitor has an equivalent series inductance of 0.371 nH, while the Skyworks SMP1340-007LF PIN diode introduces approximately 0.4 nH of parasitic inductance from its lead frame and bond wires. Similarly, the Murata LQG15HH1N0B02 inductor shows a parasitic capacitance of approximately 0.1 pF from its internal winding structure. When combined with additional parasitics from PCB pads and interconnects, the total series inductance typically falls within 0.2–1 nH and the total shunt capacitance within 0.1–0.3 pF. In contrast, micro-nano integration reduces interconnect parasitics to negligible levels. Component self-resonant frequencies increase from around 20 GHz for discrete parts (e.g., the Murata LQP03TG0N1B02) to exceed the Ka-band for integrated passives. Moreover, micro-nano fabrication integrates RLC networks within a footprint below 1 mm^2, rather than the 1–2 mm^2 required by surface-mount processes, achieving a level of circuit complexity and

integration density that the latter cannot match. The use of packaged components at high frequencies often introduces significant performance degradation due to these parasitic effects. In contrast, micro-nano integration minimizes such parasitics, ensuring better agreement between simulated and measured responses....

Comment 5: *Some works missing from the references include:*
- "A Generative Machine Learning-Based Approach for Inverse Design of Multilayer Metasurfaces," in IEEE Transactions on Antennas and Propagation, vol. 69, no. 9, pp. 5725-5739, Sept. 2021, doi: 10.1109/TAP.2021.3060142
-"Synthesis of multi-band reflective polarizing metasurfaces using a generative adversarial network"

Response: We thank the reviewer for this suggestion. Both works have been cited in the revised introduction to provide broader context for AI-driven metasurface design.

Added references:

[26] P. Naseri and S. V. Hum: A generative machine learning-based approach for inverse design of multilayer metasurfaces. IEEE Trans. Antennas Propag. **69**(9), 5725–5739 (2021). https://doi.org/10.1109/TAP.2021.3060142

[27]Naseri, P., Goussetis, G., Fonseca, N.J.G. et al. Synthesis of multi-band reflective polarizing metasurfaces using a generative adversarial network. Sci Rep **12**, 17006 (2022). https://doi.org/10.1038/s41598-022-20851-y
